# Treatment of knee osteoarthritis with intra-articular injection of allogeneic adipose-derived stem cells (ADSCs) ELIXCYTE®: a phase I/II, randomized, active-control, single-blind, multiple-center clinical trial

**DOI:** 10.1186/s13287-021-02631-z

**Published:** 2021-10-30

**Authors:** Cheng-Fong Chen, Chih-Chien Hu, Chen-Te Wu, Hung-Ta H. Wu, Chun-Shin Chang, Yi-Pei Hung, Chia-Chu Tsai, Yuhan Chang

**Affiliations:** 1 Division of Joint Reconstruction, Department of Orthopedics and Traumatology, Taipei Veterans General Hospital, Taipei, Taiwan; 2 Department of Surgery, School of Medicine, National Yang Ming Chiao Tung University, Taipei, Taiwan; 3 Division of Joint Reconstruction, Department of Orthopedic Surgery, Chang Gung Memorial Hospital, Linkou, Taiwan; 4 Bone and Joint Research Center, Chang Gung Memorial Hospital, Linkou, Taiwan; 5 College of Medicine, Chang Gung University, Taoyuan, Taiwan; 6 Department of Medical Imaging and Intervention, Chang Gung Memorial Hospital, Linkou, Taiwan; 7 Department of Radiology, Taipei Veterans General Hospital, Taipei, Taiwan; 8 Department of Radiology, School of Medicine, National Yang Ming Chiao Tung University, Taipei, Taiwan; 9 Craniofacial Research Center, Department of Medical Research, Department of Plastic and Reconstructive Surgery and Department of Craniofacial Orthodontics, Chang Gung Memorial Hospital, Taoyuan, Taiwan; 10 Department of Chemical and Materials Engineering, College of Engineering, Chang Gung University, Taoyuan, Taiwan; 11 UnicoCell BioMed Co. Ltd., Taipei, Taiwan

**Keywords:** Knee osteoarthritis, Adipose tissue-derived stem cells, ADSCs, ELIXCYTE®, Hyaluronic acid, HA, WOMAC, VAS, KSCRS

## Abstract

**Objective:**

To evaluate the safety and efficacy of intra-articular (IA) injection of allogeneic adipose-derived stem cells (ADSCs) ELIXCYTE® for knee osteoarthritis.

**Methods:**

This was a patient-blind, randomized, active-control trial consisted of 4 arms including hyaluronic acid (HA) control and 3 ELIXCYTE® doses. A total of 64 subjects were screened, and 57 subjects were randomized. The primary endpoints included the changes from baseline to post-treatment visit of Western Ontario and McMaster Universities Osteoarthritis Index (WOMAC) pain score at Week 24 and the incidence of adverse events (AEs) and serious adverse events (SAEs).

**Results:**

No ELIXCYTE®-related serious adverse events were reported during 96 weeks of follow-up and no suspected unexpected serious adverse reaction (SUSAR) or death was reported. The changes of the primary endpoint, WOMAC pain score at Week 24, showed significant differences in all ELIXCYTE® groups, as well as in HA groups between post-treatment visit and baseline. The ELIXCYTE® groups revealed significant decreases at Week 4 compared to HA group in WOMAC total scores, stiffness scores, functional limitation scores suggested the potential of ELIXCYTE® in earlier onset compared to those from HA. The significant differences of visual analog scale (VAS) pain score and Knee Society Clinical Rating System (KSCRS) functional activities score at Week 48 after ELIXCYTE® administration suggested the potential of ELIXCYTE® in the longer duration of the effectiveness compared to HA group.

**Conclusions:**

ELIXCYTE® for knee osteoarthritis treatment was effective, safe, and well-tolerated. The efficacy results were showed that ELIXCYTE® conferred the earlier onset of reductions in pain scores and improvements in functional scores than HA group.

*Trial registration*: ClinicalTrials.gov Identifier: NCT02784964. Registered 16 May, 2016—Retrospectively registered, https://clinicaltrials.gov/ct2/show/NCT02784964

**Supplementary Information:**

The online version contains supplementary material available at 10.1186/s13287-021-02631-z.

## Background

Knee osteoarthritis (KOA) is a chronic musculoskeletal disease that affects 7% of the global population, more than 500 million people, and has been recognized as the 15th highest cause of years lived with disability (YLDs) worldwide [1]. Current managements of KOA are aimed at pain reduction and symptom control rather than disease modification. Nonsteroidal anti-inflammatory drugs (NSAIDs), acetaminophen, and corticosteroids are generally used, and patients are unsatisfied with their limited analgesic, short-term efficacy, and potential risk of gastrointestinal and cardiovascular disorder [2, 3]. Hyaluronic acid (HA) is a natural component that seems to be attributable to KOA therapy via its viscosupplementation properties. Even though the use of intra-articular HA has raised no major safety concerns so far, the efficacy is still controversial [4].

Novel findings on the mechanisms underlying the development of KOA promote the research of potential disease-modifying osteoarthritis drugs (DMOADs) which targeting osteoarthritis pathogenesis, such as cartilage destruction, subchondral bone remodeling, and synovial inflammation. These DMOADs are pursued not only to relief pain and improve joint function, but also inhibit the progression of structural disorder [5]. Even if no DMOADs have been licensed, several potential candidates are under development [2, 5].

Mesenchymal stem cells (MSCs) have been shown to possess broad immunoregulatory and anti-inflammatory abilities and are capable to suppress all immune cells that play an important pathogenic role in the development and progression of OA [6]. In addition, MSCs promote endogenous proliferation of chondrocyte and inhibit chondrocyte apoptosis or cartilage degeneration by paracrine signaling, achieving cartilage regeneration and cartilage protection [7, 8]. These mechanism of actions of MSCs may not only provide the effective improvement of pain and symptom control, but also provide the potential disease-modifying effects on KOA.

Preclinical research has successfully shown the benefit of intra-articular MSC therapy for OA in pain relief and functional improvement [9–12]. Moreover, the initial clinical trials have an encouraging outcome on pain relief and functional improvement in symptomatic knee OA following MSC therapy [13–16]. While most studies have used autologous MSCs, allogeneic MSCs appear to have an acceptable safety profile which is similar to autologous cells. It is worth mentioning that allogeneic MSCs allow the manufacturing of large batches of off-the-shelf MSC products, which would enhance the consistency and decrease the costs of cell therapy, and solve the problem of limited proliferation capability of MSCs from elder donors. So far, the potential concern of using allogeneic adipose-derived stem cells (ADSCs) and the data of allogenic MSC for the KOA treatment is relatively limited as compared with the autologous MSCs [14, 17–22]. Therefore, we conducted a randomized Phase I/II clinical trial to assess the safety and therapeutic potential of allogeneic ADSCs in patients with KOA.

## Methods

### Preparation of ELIXCYTE®

The donor who signed the informed consent for the preparation of ELIXCYTE was recruited under the supervision of an Institutional Review Board (CGMF-IRB Number: 104-1790A3) and was screened and tested in compliance with eligibility determination guidance issued by Taiwan Food and Drug Administration. The adipose tissue was collected from eligible donor via ultrasonic-assisted liposuction and was transferred to UnicoCell BioMed Company under hypothermal condition within 6 h. The stromal vascular fraction (SVF) was isolated by digesting the adipose tissue with type I collagenase, centrifuged, and cultured at 37 °C in a humidified carbon dioxide incubator for primary culture. The adherent and highly proliferated cells termed ADSCs were detached and propagated to the passage 7 and within predefined population doubling level. The ADSCs were then harvested and formulated at a density of 8 × 10^6^ cells/mL with cryoprotectant CryoStor® CS10 (BioLife Solutions). Release testing of ELIXCYTE® for microbiological evaluation (including mycoplasma, sterility, and endotoxin tests) was employed to ensure safety. The product characteristics such as identity (including MSC markers, viable cell count, and cell viability), and tri-lineage differentiation properties were also assessed.

### Trial design

This phase I/II study, named as adipose-derived stem cells (ADSCs) for knee osteoarthritis, was planned as a randomized, single-blind, active-control study to evaluate the safety and efficacy of allogeneic ADSCs (ELIXCYTE®) intra-articular injection to patients with KOA. The study was registered on clinicaltrail.gov (NCT02784964) and was approved by Institutional Review Board to the tenets of the Declaration of Helsinki. There were two study sites in this trial, including Linkou Chang Gung Memorial Hospital and Taipei Veterans General Hospital in Taiwan. Permuted block randomization stratified by the center method was applied to generate randomization codes before study start, statistician from contract research organization (CRO) generated the random allocation sequence, investigators of this study enrolled participants, and the sponsor who assigned participants to interventions. Phase I of this study schemed to recruit 6 evaluable patients, and the permute block size was 6. Eligible patients would be randomly assigned in 2:1 ratio to investigational product treatment group (ELIXCYTE® 64 × 10^6^ cells, 64 M) or active-control group (Hya Joint Plus synovial fluid supplement 3 mL, SciVision Biotech Inc).

Phase II of this study schemed to recruit 36 evaluable patients, and the permute block size was 9. Eligible patients would be randomly assigned in 2:3:3:1 ratio to investigational product treatment group (ELIXCYTE® 64 × 10^6^ cells, 64 M; ELIXCYTE® 32 × 10^6^ cells, 32 M; ELIXCYTE® 16 × 10^6^ cells, 16 M) or active-control group. Each eligible patient would have exactly one “target knee” for efficacy evaluation for this study. The target knee would be the knee meeting entry criteria and be administered on day 1 with the treatment upon the patient was randomly assigned. Subjects were followed for 96 weeks including scheduled visits at Weeks 2, 4, 12, 24 (main study), 36, 48 (extension study), 72, and 96 (structural study) after administration.

### Participants

This study was planned to enroll about 56 subjects for 42 evaluable subjects. All subjects gave written informed consent to participate in the study prior to enrollment. Eligible participants included patients aged 40–80 years (inclusive) with Kellgren–Lawrence (KL) grading I–III, as determined by American College of Rheumatology (ACR) criteria for KOA and WOMAC pain score 7–17. We excluded those with surgery history on the target knee joint, previous intra-articular intervention on the target knee joint within past 3 months, hypersensitivity to any component used in the study, inadequate hematologic and hepatic function, human immunodeficiency virus (HIV) infection or body mass index (BMI) greater than 35 kg/m^2^. Patients already participated in any other interventional study within 4 weeks of entering the study were also excluded. Treatment applied to target knee area, or analgesics except for acetaminophen and NSAIDs would be prohibited from patients.

### Efficacy outcome measures

The primary endpoint was the change in WOMAC pain score from baseline to week 24 after treatment. Secondary outcomes included change from baseline to post-treatment visit in WOMAC stiffness, physical function, and total score; VAS for pain and KSCRS score.

### Safety assessment

The safety assessment was the incidence of AEs and SAEs.

### Clinical laboratory evaluation

Laboratory assessments including hematology and biochemistry were performed at the Screening, Week 24, 36, 48, 72, and 96 visits. The measured items were listed as below: for hematology: white blood cells (WBC), neutrophils, lymphocytes, monocytes, eosinophils, basophils, hemoglobin, hematocrit, platelet, and red blood cells (RBC); for biochemistry: aspartate aminotransferase, alanine aminotransferase, serum creatinine, blood urea nitrogen (BUN), and albumin; for immunogenicity, tumor necrosis factor alpha (TNF-α), CD4, and CD8 were measured at screening, Week 4, and 24 visits.

### Statistical analysis

All phase I and phase II data were pooled for statistical analysis. Efficacy endpoints and safety endpoints were analyzed on the intent-to-treat (ITT) population. Demographics and baseline characteristics were analyzed by using a two-sample t-test, Wilcoxon rank-sum test or Fisher’s exact test as appropriate to ensure comparability between treatments. A two-sample t-test was applied for *p*-value (pairwise) in the statistic table, Wilcoxon rank-sum test was applied if the normality assumption was not valid (*p*-value of Shapiro–Wilk normality test ≤ 0.05). The efficacy endpoints, changes in WOMAC, VAS and KSCRS were analyzed by using ANCOVA incorporating treatment effect and baseline as covariate or by Wilcoxon rank-sum test. For safety analyses, the incidence of adverse events was tabulated by treatment groups and by physiological systems as appropriate.

Pairwise treatment group comparisons were conducted with a significance level of 0.05 without alpha adjustment. For efficacy endpoints, a 95% two-sided confidence interval on the difference of each treatment was provided as appropriate.

## Results

### Patient disposition and baseline characteristics

**Fig. 1:**
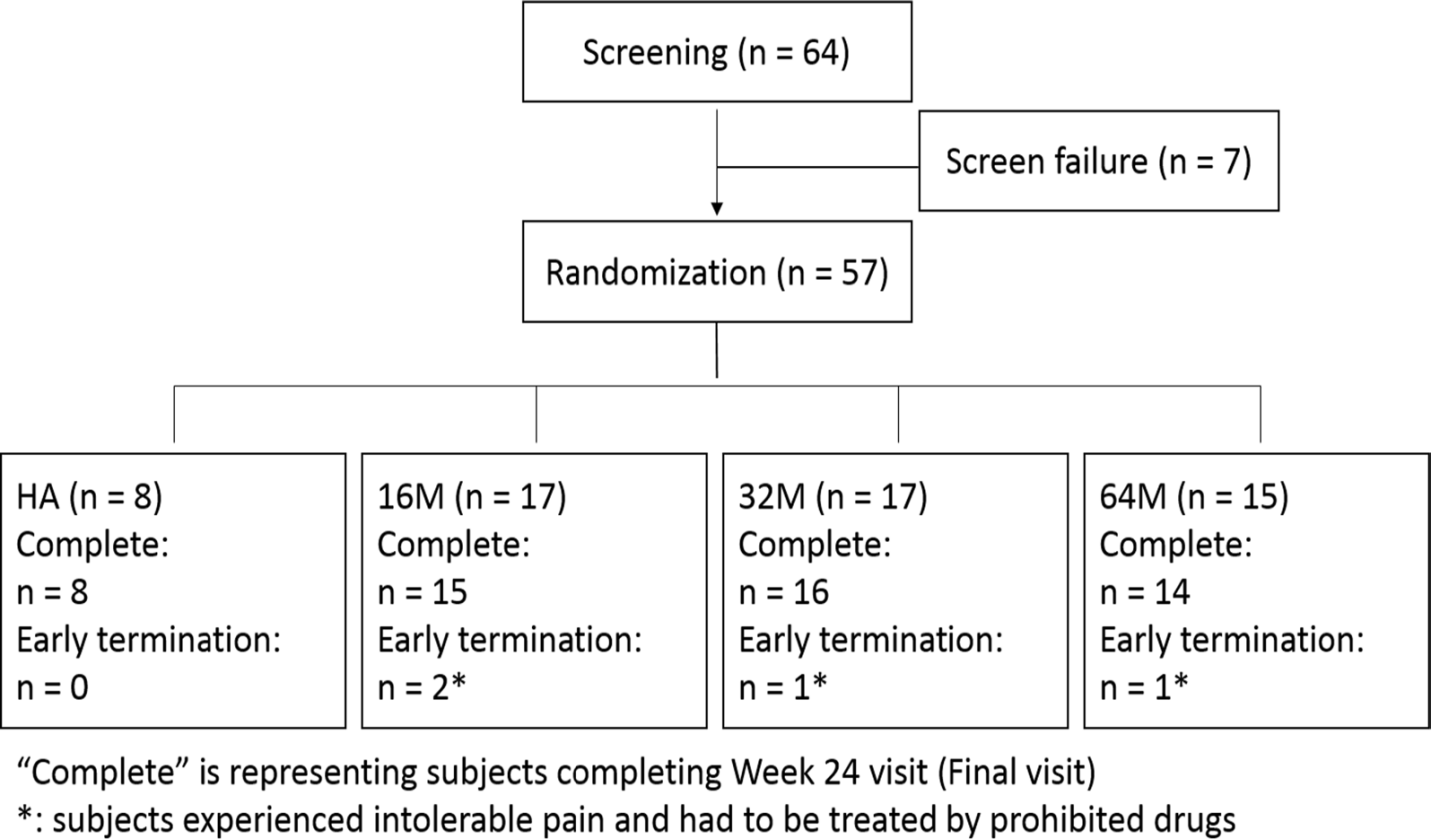
Patient flow diagram showing subject numbers for screening, randomization, and treatment assignment

Total 64 subjects were screened, where 7 were screen failures, resulted in 57 (89%) subjects being eligible and randomized. Among them, 8 (14%) subjects received HA and 17 (29.8%) subjects received ELIXCYTE® 16 M, 17 (29.8%) subjects received ELIXCYTE® 32 M, and 15 (26.3%) subjects received ELIXCYTE® 64 M within the study period (Fig. 1). The ITT population consisted of the 57 randomized patients who received at least one dose of single-blind treatment.

Table 1 shows the demographic and baseline characteristics of the ITT patient population. Among 57 enrolled study subjects, 11 (19.3%) were male and 46 (80.7%) were female. The 57 eligible subjects were (mean ± SD) 67.6 ± 6.60 years of age, 155.2 ± 7.92 cm in height, 63.9 ± 9.64 kg in weight, and 26.54 ± 3.673 kg/m^2^ in BMI. The baseline KL grade of the target knees was either grade II (37/57, 64.9%) or grade III (20/57, 35.1%). The average duration of KOA since diagnosed was 2.96 years. No particular differences between individual ELIXCYTE® dose and HA groups were observed in demographic data and KOA history.

**Table 1:** Summary of demographic data—ITT population at the screening visit

Demographic data	ITT population				
	HA	16 M	32 M	64 M	Total
N (Missing)	8 (0)	17 (0)	17 (0)	15 (0)	57 (0)
*Age (year)*					
Mean (SD)	70.5 (8.37)	67.7 (6.84)	68.6 (6.45)	64.9 (4.91)	67.6 (6.60)
Median (IQR)	73.5 (13.0)	66.0 (5.0)	68.0 (9.0)	65.0 (8.0)	67.0 (10.0)
Q1 ~ Q3	64.0 ~ 77.0	65.0 ~ 70.0	65.0 ~ 74.0	60.0 ~ 68.0	63.0 ~ 73.0
Min ~ Max	56.0 ~ 79.0	51.0 ~ 78.0	55.0 ~ 78.0	59.0 ~ 75.0	51.0 ~ 79.0
*p*-value (T test)	–	0.3835	0.5356	0.0531	–
*Gender*					
Male	3 (37.5%)	3 (17.6%)	2 (11.8%)	3 (20.0%)	11 (19.3%)
Female	5 (62.5%)	14 (82.4%)	15 (88.2%)	12 (80.0%)	46 (80.7%)
*p*-value (FISHER)	–	0.3442	0.2833	0.6214	–
*Body height (cm)*					
Mean (SD)	154.0 (12.42)	154.7 (7.51)	155.0 (6.75)	156.5 (7.36)	155.2 (7.92)
Median (IQR)	148.8 (23.9)	155.0 (7.5)	153.5 (6.9)	155.4 (8.5)	154.2 (9.1)
Q1 ~ Q3	143.8 ~ 167.7	149.5 ~ 157.0	151.0 ~ 157.9	152.0 ~ 160.5	149.5 ~ 158.6
Min ~ Max	141.0 ~ 170.3	142.0 ~ 177.2	147.7 ~ 176.0	145.3 ~ 168.3	141.0 ~ 177.2
*p*-value (Wilcox_t)	–	0.5275	0.3907	0.4656	–
*Body weight (Kg)*					
Mean (SD)	60.5 (10.80)	66.0 (6.80)	64.2 (10.97)	62.9 (10.47)	63.9 (9.64)
Median (IQR)	64.3 (18.5)	65.0 (6.9)	63.2 (13.1)	63.8 (16.6)	63.8 (12.9)
Q1 ~ Q3	49.4 ~ 67.8	62.0 ~ 68.9	56.4 ~ 69.5	55.7 ~ 72.3	56.6 ~ 69.5
Min ~ Max	46.2 ~ 74.6	55.6 ~ 77.4	48.0 ~ 87.2	42.3 ~ 80.2	42.3 ~ 87.2
*p*-value (T test)	–	0.1321	0.4293	0.6015	–
*BMI (Kg/m*^*2*^*)*					
Mean (SD)	25.47 (3.494)	27.65 (3.026)	26.72 (4.192)	25.66 (3.782)	26.54 (3.673)
Median (IQR)	24.64 (3.89)	28.28 (4.68)	25.98 (3.68)	25.67 (5.78)	25.98 (5.22)
Q1 ~ Q3	23.33 ~ 27.21	25.03 ~ 29.70	24.56 ~ 28.24	22.90 ~ 28.68	23.83 ~ 29.05
Min ~ Max	21.09 ~ 32.29	23.14 ~ 32.26	20.37 ~ 34.59	19.39 ~ 31.33	19.39 ~ 34.59
*p*-value (T test)	–	0.1232	0.4724	0.9088	–
*Kellgren–Lawrence grade on target knee*					
Grade II	5 (62.5%)	10 (58.8%)	10 (58.8%)	12 (80.0%)	37 (64.9%)
Grade III	3 (37.5%)	7 (41.2%)	7 (41.2%)	3 (20.0%)	20 (35.1%)
*p*-value (FISHER)	–	1.0000	1.0000	0.6214	–
*Disease duration (years)*					
Mean (SD)	2.44 (2.367)	2.97 (1.755)	3.68 (7.198)	2.39 (1.634)	2.96 (4.164)
Median (IQR)	2.05 (2.24)	2.97 (2.13)	2.04 (2.48)	2.08 (2.48)	2.26 (2.24)
Q1 ~ Q3	0.81 ~ 3.05	2.06 ~ 4.20	0.20 ~ 2.68	1.12 ~ 3.59	1.12 ~ 3.36
Min ~ Max	0.10 ~ 7.58	0.00 ~ 5.87	0.00 ~ 29.68	0.16 ~ 5.86	0.00 ~ 29.68
*p*-value (Wilcox_t)	–	0.3755	0.7514	0.8733	–

For CI and *p*-value (between groups), FISHER denotes Fisher's exact test, T denotes two-sample t-test, Wilcox_t denotes Wilcoxon rank-sum test (Mann–Whitney U Test) in *t* approximation

### Safety

The summary of all treatment-emergent adverse events (TEAEs) during Week 0–24 and Week 24–96 is listed in Table 2, and all the treatment-related AEs were observed during week 0–24. Forty-four subjects (77.2%) reported at least one TEAE and 25 subjects (43.9%) experiencing at least one treatment-related AEs during Week 0–24. There were 3 subjects (5.3%) reported AEs with grade ≥ 3, including 2 subjects (11.8%) in middle-dose group and 1 subject (6.7%) in high-dose group, which was judged as treatment-related AE. During Week 24–96, 31 subjects (54.4%) reported at least one TEAEs. There were 6 SAEs recorded during the whole study period (Week 0–96), and none of them were judged as treatment-related SAE. Moreover, no SUSAR or death was reported in this study.

**Table 2:** Summary of treatment-emergent adverse events (TEAEs)

Treatment-emergent adverse events	HA N = 8		16 M N = 17		32 M N = 17		64 M N = 15		Total N = 57	
[Event # (E)/subject # (S) (%)]	E	S (%)	E	S (%)	E	S (%)	E	S (%)	E	S (%)
*Week 0–24 (early TEAEs)*										
Subjects with AEs	11	4 (50.0%)	40	15 (88.2%)	41	14 (82.4%)	25	11 (73.3%)	117	44 (77.2%)
Subjects with treatment-related AEs	1	1 (12.5%)	12	6 (35.3%)	16	10 (58.8%)	17	8 (53.3%)	46	25 (43.9%)
Subjects with grade ≥ 3 AEs	0	0 (0.0%)	0	0 (0.0%)	2	2 (11.8%)	1	1 (6.7%)	3	3 (5.3%)
Subjects with grade ≥ 3 treatment-related AEs	0	0 (0.0%)	0	0 (0.0%)	0	0 (0.0%)	1	1 (6.7%)	1	1 (1.8%)
Subjects with treatment-modified AE	0	0 (0.0%)	0	0 (0.0%)	0	0 (0.0%)	0	0 (0.0%)	0	0 (0.0%)
Subjects with SAEs	0	0 (0.0%)	0	0 (0.0%)	1	1 (5.9%)	0	0 (0.0%)	1	1 (1.8%)
Subjects with SUSARs	0	0 (0.0%)	0	0 (0.0%)	0	0 (0.0%)	0	0 (0.0%)	0	0 (0.0%)
*Week 24–96 (Long-term TEAEs)*										
Subjects with AEs	16	7 (87.5%)	19	7 (41.2%)	23	8 (47.1%)	25	9 (60.0%)	83	31 (54.4%)
Subjects with SAEs	0	0 (0.0%)	0	0 (0.0%)	3	2 (11.8%)	2	2 (13.3%)	5	4 (7.0%)
Subjects with SUSARs	0	0 (0.0%)	0	0 (0.0%)	0	0 (0.0%)	0	0 (0.0%)	0	0 (0.0%)

Treatment-related TEAEs included possible, probable, and definite in relation to study treatment

AE, adverse event; TEAE, treatment emergent adverse event; SAE, serious adverse event; SUSAR, suspected unexpected serious adverse reaction

Table 3 shows the TEAEs during Week 0–24 by system organ class (SOC). The most frequent TEAEs during Week 0–24 in total population were “Musculoskeletal and connective tissue disorders” (40.4%), followed by “General disorders and administration site conditions” (29.8%), “Infections and infestation” (21.1%), and “Gastrointestinal disorders” (12.3%). The frequency of other TEAEs was lower than 10.0%. TEAEs observed during Week 24–96 are presented in Additional file 2: Table S1.

**Table 3:** Summary of adverse events during week 0–24 (early TEAEs)

Adverse event (Week 0–24)	HA *N* = 8		16 M *N* = 17		32 M *N* = 17		64 M *N* = 15		Total *N* = 57	
[Event # (E)/Subject # (S) (%)]	E	S (%)	E	S (%)	E	S (%)	E	S (%)	E	S (%)
At least one below	**11**	**4 (50.0%)**	**40**	**15 (88.2%)**	**41**	**14 (82.4%)**	**25**	**11 (73.3%)**	**117**	**44 (77.2%)**
Cardiac disorders	1	1 (12.5%)	0	0 (0.0%)	1	1 (5.9%)	0	0 (0.0%)	2	2 (3.5%)
Eye disorders	1	1 (12.5%)	2	2 (11.8%)	0	0 (0.0%)	0	0 (0.0%)	3	3 (5.3%)
Gastrointestinal disorders	1	1 (12.5%)	3	2 (11.8%)	4	3 (17.6%)	1	1 (6.7%)	9	7 (12.3%)
General disorders and administration site conditions	1	1 (12.5%)	4	3 (17.6%)	7	7 (41.2%)	7	6 (40.0%)	19	17 (29.8%)
Hepatobiliary disorders	0	0 (0.0%)	0	0 (0.0%)	1	1 (5.9%)	0	0 (0.0%)	1	1 (1.8%)
Infections and infestations	1	1 (12.5%)	6	6 (35.3%)	3	2 (11.8%)	4	3 (20.0%)	14	12 (21.1%)
Injury, poisoning, and procedural complications	0	0 (0.0%)	2	1 (5.9%)	1	1 (5.9%)	0	0 (0.0%)	3	2 (3.5%)
Investigations	0	0 (0.0%)	0	0 (0.0%)	1	1 (5.9%)	0	0 (0.0%)	1	1 (1.8%)
Metabolism and nutrition disorders	0	0 (0.0%)	2	1 (5.9%)	0	0 (0.0%)	0	0 (0.0%)	2	1 (1.8%)
Musculoskeletal and connective tissue disorders	5	2 (25.0%)	15	6 (35.3%)	13	10 (58.8%)	11	5 (33.3%)	44	23 (40.4%)
Neoplasms benign, malignant, and unspecified (including cysts and polyps)	0	0 (0.0%)	2	2 (11.8%)	1	1 (5.9%)	0	0 (0.0%)	3	3 (5.3%)
Nervous system disorders	1	1 (12.5%)	1	1 (5.9%)	2	2 (11.8%)	1	1 (6.7%)	5	5 (8.8%)
Psychiatric disorders	0	0 (0.0%)	0	0 (0.0%)	1	1 (5.9%)	0	0 (0.0%)	1	1 (1.8%)
Renal and urinary disorders	0	0 (0.0%)	0	0 (0.0%)	1	1 (5.9%)	0	0 (0.0%)	1	1 (1.8%)
Respiratory, thoracic, and mediastinal disorders	0	0 (0.0%)	1	1 (5.9%)	2	2 (11.8%)	1	1 (6.7%)	4	4 (7.0%)
Skin and subcutaneous tissue disorders	0	0 (0.0%)	1	1 (5.9%)	1	1 (5.9%)	0	0 (0.0%)	2	2 (3.5%)
Surgical and medical procedures	0	0 (0.0%)	1	1 (5.9%)	2	1 (5.9%)	0	0 (0.0%)	3	2 (3.5%)

Dictionary: MedDRA version 19.0

As for treatment-related AEs, the most frequent AEs were “Injection site joint pain” (15.8%), followed by “Arthralgia” (14.0%), “Injection site joint swelling” (12.3%), and “Joint swelling” (10.5%). The frequency of other treatment-related AEs was lower than 5%. There was no treatment-related AE reported during Week 24–96. The summary of treatment-related AEs is presented in Table 4.

**Table 4:** Summary of treatment-related adverse events during week 0–24

Treatment-relative adverse event (week 0–24)	HA *N* = 8		16M *N* = 17		32M *N* = 17		64M *N* = 15		Total *N* = 57	
[Event # (E)/subject # (S) (%)]	E	S (%)	E	S (%)	E	S (%)	E	S (%)	E	S (%)
At least one below	**1**	**1 (12.5%)**	**12**	**6 (35.3%)**	**16**	**10 (58.8%)**	**17**	**8 (53.3%)**	**46**	**25 (43.9%)**
General disorders and administration site conditions	1	1 (12.5%)	4	3 (17.6%)	7	7 (41.2%)	7	6 (40.0%)	19	17 (29.8%)
~ Injection site erythema	0	0 (0.0%)	0	0 (0.0%)	1	1 (5.9%)	0	0 (0.0%)	1	1 (1.8%)
~ Injection site joint effusion	0	0 (0.0%)	0	0 (0.0%)	0	0 (0.0%)	1	1 (6.7%)	1	1 (1.8%)
~ Injection site joint inflammation	0	0 (0.0%)	1	1 (5.9%)	0	0 (0.0%)	0	0 (0.0%)	1	1 (1.8%)
~ Injection site joint pain	1	1 (12.5%)	1	1 (5.9%)	3	3 (17.6%)	4	4 (26.7%)	9	9 (15.8%)
~ Injection site joint swelling	0	0 (0.0%)	2	2 (11.8%)	3	3 (17.6%)	2	2 (13.3%)	7	7 (12.3%)
Infections and infestations	0	0 (0.0%)	1	1 (5.9%)	0	0 (0.0%)	0	0 (0.0%)	1	1 (1.8%)
~ Pharyngitis	0	0 (0.0%)	1	1 (5.9%)	0	0 (0.0%)	0	0 (0.0%)	1	1 (1.8%)
Injury, poisoning, and procedural complications	0	0 (0.0%)	0	0 (0.0%)	1	1 (5.9%)	0	0 (0.0%)	1	1 (1.8%)
~ Procedural vomiting	0	0 (0.0%)	0	0 (0.0%)	1	1 (5.9%)	0	0 (0.0%)	1	1 (1.8%)
Investigations	0	0 (0.0%)	0	0 (0.0%)	1	1 (5.9%)	0	0 (0.0%)	1	1 (1.8%)
~ Alpha tumor necrosis factor increased	0	0 (0.0%)	0	0 (0.0%)	1	1 (5.9%)	0	0 (0.0%)	1	1 (1.8%)
Musculoskeletal and connective tissue disorders	0	0 (0.0%)	4	2 (11.8%)	6	5 (29.4%)	10	4 (26.7%)	20	11 (19.3%)
~ Arthralgia	0	0 (0.0%)	3	2 (11.8%)	2	2 (11.8%)	6	4 (26.7%)	11	8 (14.0%)
~ Joint stiffness	0	0 (0.0%)	0	0 (0.0%)	1	1 (5.9%)	2	1 (6.7%)	3	2 (3.5%)
~ Joint swelling	0	0 (0.0%)	1	1 (5.9%)	3	3 (17.6%)	2	2 (13.3%)	6	6 (10.5%)
Neoplasms benign, malignant, and unspecified (including cysts and polyps)	0	0 (0.0%)	1	1 (5.9%)	0	0 (0.0%)	0	0 (0.0%)	1	1 (1.8%)
~ Pharyngeal neoplasm benign	0	0 (0.0%)	1	1 (5.9%)	0	0 (0.0%)	0	0 (0.0%)	1	1 (1.8%)
Nervous system disorders	0	0 (0.0%)	1	1 (5.9%)	1	1 (5.9%)	0	0 (0.0%)	2	2 (3.5%)
~ Dizziness	0	0 (0.0%)	1	1 (5.9%)	1	1 (5.9%)	0	0 (0.0%)	2	2 (3.5%)
Skin and subcutaneous tissue disorders	0	0 (0.0%)	1	1 (5.9%)	0	0 (0.0%)	0	0 (0.0%)	1	1 (1.8%)
~ Cold sweat	0	0 (0.0%)	1	1 (5.9%)	0	0 (0.0%)	0	0 (0.0%)	1	1 (1.8%)

*Dictionary: MedDRA version 19.0*

Additional file 1 showed that there was no clinically significant (NCS) abnormality in hematology observed in the subjects during the study, and most biochemistry values were normal or abnormal but NCS. However, the abnormal and clinically significant laboratory values of aspartate aminotransferase (AST) were observed in 1 (7.7%) subject in 32M and 1 (8.3%) subject in the 64M at Week 48 which were not treatment related. The CS values of ALT were also observed in 1 (7.7%) subject in the 32M and 1 (8.3%) subject in the 64M at Week 48. Regarding immunogenicity, most biochemistry values were normal or abnormal but NCS, except 1 (5.9%) abnormal and clinically significant TNF-α result was found in the 32M group at Week 4. These abnormal values were mainly related to medical history or adverse events of the subjects (Additional file 1, Table 18).

### Efficacy: primary outcomes

**Fig. 2:**
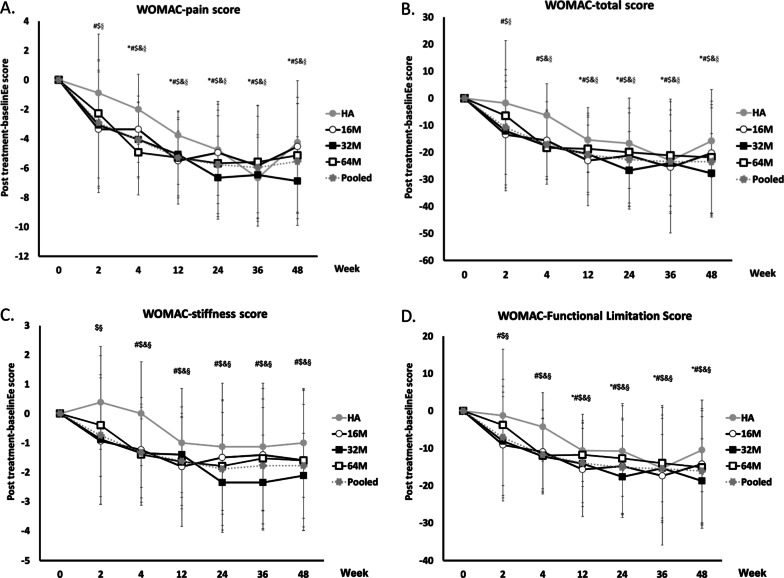
Changes in WOMAC after intra-articular injection of ELIXCYTE® or HA at each time point. WOMAC pain (**a**), total (**b**), stiffness (**c**), and function limitation (**d**) scores are presented as mean ± SD. 16 M, 32 M, 64 M, and HA = 16, 32, 64 million of ELIXCYTE®, and hyaluronic acid, respectively. The signs of significance, *, #, $, &, § representing a *p*-value < 0.05 between baseline and posttreatment score comparison for HA, 16 M, 32 M, 64 M, and Pooled group

The WOMAC pain score at week 24 was used to evaluate the primary endpoint. Under no significant difference between baseline values of HA and all ELIXCYTE® groups, all groups had significant reductions of pain score from baseline to post-treatment through Week 4 to 48 (Additional file 2: Table S2 for detail *p*-value information)**.** In addition, the earlier significant reductions of pain score at Week 2 were observed in 16 M, 32 M, and Pooled groups compared to those in HA group (16 M: *P* = 0.0024; 32 M: *P* = 0.0040; Pooled *P* < 0.0001)**.** The significant difference in mean WOMAC pain scores was observed at Week 4 in 64 M and Pooled groups in comparison with the HA group (64 M: *P* = 0.0026; Pooled: *P* = 0.0381) **(**Fig. 2a, Additional file 2: Table S2).

### Efficacy: secondary outcomes

The baseline values of HA and all ELIXCYTE® groups were similar in WOMAC score (total, stiffness, and functional limitation), VAS score for pain, and KSCRS score (objective knee indicators, symptoms, and functional activities), except KSCRS score-patient satisfaction for the 16 M (*P* = 0.0253). (Individual *p* values are shown in supplement tables.)

All ELIXCYTE® groups had a significant reduction in WOMAC total score, stiffness, and functional limitation score from baseline to post-treatment at Week 12 to 48 (Additional file 2: Table S3, S4, and S5 for detailed *p*-value information). The earlier significant decreases of WOMAC total score from baseline to post-treatment were observed at Week 2 in 16 M, 32 M, and Pooled groups (16 M: *P* = 0.0129; 32 M: *P* = 0.0088; Pooled *P* < 0.0001), and at Week 4 in 16 M, 32 M, 64 M, and Pooled groups (16 M: *P* = 0.0004; 32 M: *P* < 0.0001; 64 M: *P* = 0.0001; Pooled *P* < 0.0001). A significant difference in mean WOMAC total scores was observed at Week 4 in 16 M, 32 M, 64 M, Pooled groups (16 M: *P* = 0.0481; 32 M: *P* = 0.0390; 64 M: *P* = 0.0014; Pooled *P* = 0.0075), and Week 48 in 64 M group in comparison with HA group (*P* = 0.0113) (Fig. 2b, Additional file 2: Table S3). A significant difference of mean WOMAC stiffness scores was observed at Week 4 in 16 M, 32 M, 64 M, and Pooled groups (16 M: *P* = 0.0369; 32 M: *P* = 0.0360; 64 M: *P* = 0.0027; Pooled *P* = 0.0073), and at Week 48 in 64 M in comparison with the HA group (*P* = 0.0402) (Fig. 2C, Additional file 2: Table S4). In WOMAC functional limitation score, the earlier improvement from baseline to post-treatment was found at Week 2 in 16 M, 32 M, and Pooled groups (16 M: *P* = 0.0173; 32 M: *P* = 0.0155; Pooled *P* < 0.0001), and at Week 4 in 16 M, 32 M, 64 M, and Pooled groups (16 M: *P* = 0.0010; 32 M: *P* < 0.0001; 64 M: *P* = 0.0003; Pooled *P* < 0.0001). In the comparison of HA and 64 M group at Week 48, a significant difference in the mean of functional limitation scores of WOMAC was observed (*P* = 0.0070) (Fig. 2d, Additional file 2: Table S5).

**Fig. 3:**
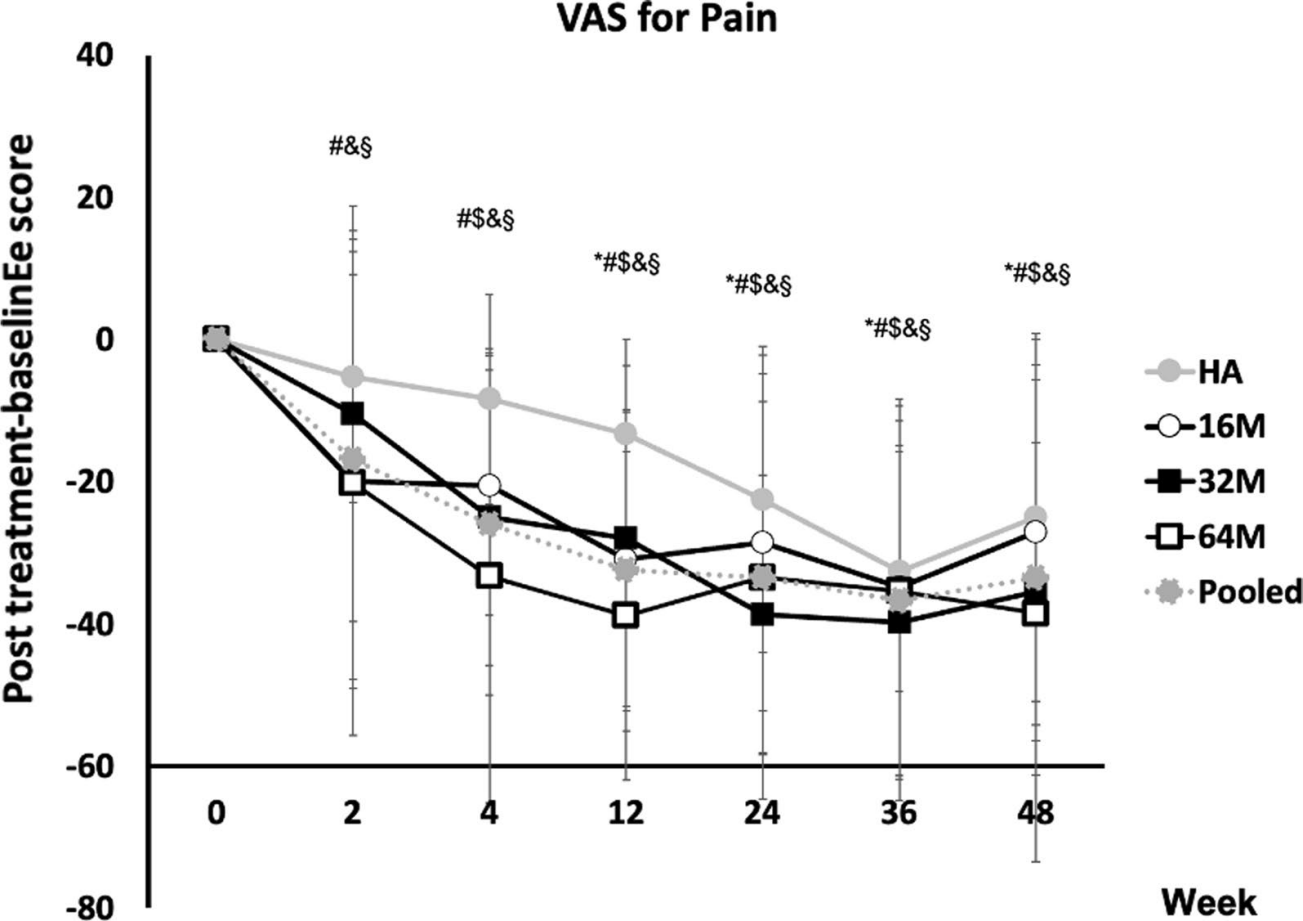
Changes in VAS pain after intra-articular injection of ELIXCYTE® or HA at each time point. VAS pain scores are presented as mean ± SD. 16 M, 32 M, 64 M, and HA = 16, 32, 64 million of ELIXCYTE®, and hyaluronic acid, respectively. The signs of significance = *, #, $, &, § representing a *p*-value < 0.05 between baseline and posttreatment score comparison for HA, 16 M, 32 M, 64 M, and Pooled group

The time to observe significant reductions of VAS pain scores from baseline to post-treatment for HA and individual ELIXCYTE® groups was Week 12 to 36, and Week 4 to 48 (Additional file 2: Table S6 for detail *p*-value information), respectively. Moreover, the earlier pain improvements were found at Week 2 in 16 M, 64 M, and Pooled groups (16 M: *P* = 0.0121; 64 M: *P* = 0.0447; Pooled: *P* = 0.0002). A significant difference in mean VAS scores was observed at Week 4 in 64 M and Pooled groups (64 M: *P* = 0.0021; Pooled: *P* = 0.0127), at Week 12 in 16 M, 32 M, 64 M, and Pooled groups (16 M: *P* = 0.0026; 32 M: *P* = 0.0439; 64 M: *P* = 0.0003; Pooled: *P* = 0.0014) in comparison with the HA group (Fig. 3, Additional file 2: Table S6).

**Fig. 4:**
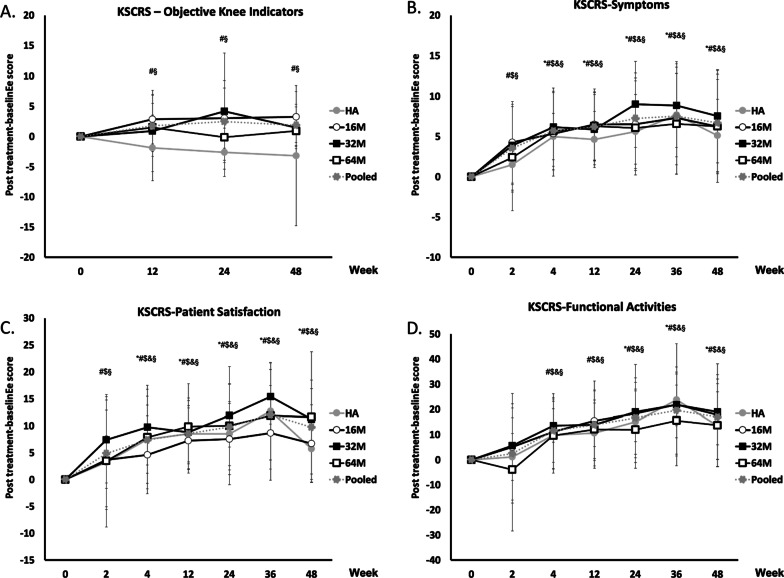
Changes in KSCRS after intra-articular injection of ELIXCYTE® or HA at each time point. KSCRS objective knee indicators (**a**), symptoms (**b**), patient satisfaction (**c**), and functional activities (**d**) scores are presented as mean ± SD. 16 M, 32 M, 64 M, and HA = 16, 32, 64 million of ELIXCYTE®, and hyaluronic acid, respectively; The signs of significance = *, #, $, &, § representing a *p*-value < 0.05 between baseline and posttreatment score comparison for HA, 16 M, 32 M, 64 M, and Pooled group

There were significant increases of KSCRS objective knee indicator scores from baseline to post-treatment in 16 M and Pooled groups at Weeks 12 to 48 (16 M: *P* = 0.0078, 0.0264, 0.0234; Pooled: *P* = 0.0021, 0.0105, 0.0002) compared to a decrease of score in HA group. A significant increase of mean KSCRS scores was observed at Week 12 in 16 M, 64 M, and Pooled groups (16 M: *P* = 0.0113; 64 M: *P* = 0.0370; Pooled: *P* = 0.0226), at Week 24 in 16 M, 32 M, and Pooled groups (16 M: *P* = 0.0017; 32 M: *P* = 0.0039; Pooled: *P* = 0.0039), and at Week 48 in 16 M (16 M: *P* = 0.0392) in comparison with the HA group. (Fig. 4a, Additional file 2: Table S7). All groups had a significant increase in KSCRS symptoms score, patient satisfaction score, and functional activities from baseline to post-treatment at Week 4 to 48 (Additional file 2: Table S8, S9, S10 for detail *p*-value information). Moreover, the earlier significant symptoms relief were found at Week 2 in 16 M, 32 M, and Pooled groups (16 M: *P* = 0.0030; 32 M: *P* = 0.0061; Pooled: *P* < 0.0001). A significant difference of mean KSCRS Symptoms scores was observed at Week 12 in 16 M, 64 M, and Pooled group (16 M: *P* = 0.0341; 64 M: *P* = 0.0080; Pooled: *P* = 0.0300), and at Week 48 in 64 M group in comparison with HA group (*P* = 0.0322) (Fig. 4b, Additional file 2: Table S8). The earlier significant improvements of patient satisfaction were found at Week 2 in 16 M, 32 M, and Pooled groups (Additional file 2: Table S9**,** 16 M: *P* = 0.0359; 32 M: *P* = 0.0022; Pooled: *P* = 0.0014). The significant increases of mean KSCRS patient satisfaction scores were observed at Week 24 in 64 M and Pooled groups (64 M: *P* = 0.0437; Pooled: *P* = 0.0397), and at Week 48 in 32 M, 64 M, and Pooled groups in comparison with HA group (32 M: *P* = 0.0472; 64 M: *P* = 0.0038; Pooled: *P* = 0.0044) (Fig. 4c, Additional file 2: Table S9). The HA group had significant increase in KSCRS functional activities score from baseline to post-treatment at Week 24 to 48 (Fig. 4d, Additional file 2: Table S10, *P* = 0.0133, 0.0023, 0.0252), while the earlier changes in the individual ELIXCYTE® and Pooled groups were observed at Week 4 and continued to Week 48 (Additional file 2: Table S10 for detail *p*-value information).

## Discussion

This present study aimed at evaluating the safety and efficacy of intra-articular ELIXCYTE® injection in patients with KOA. ELIXCYTE® for KOA treatment was shown to be effective, safe, and well-tolerated. The earlier onset and longer duration of effect mediated by ELIXCYTE® were observed compared to HA treatment and seem to show better outcomes in WOMAC scores, VAS score, and KSCRS scores.

ELIXCYTE® was shown to be safe, well-tolerated, and exhibited a comparable safety profile to the HA control. Although the allogeneic MSCs with poorly matched HLA had been reported to induce both innate and humoral responses, however, the response extent appears to be correlated with the expression level and the balance of both immune-activating antigens, such as major histocompatibility complex (MHC), and the immune-modifying cytokines, molecules and metabolites, like tumor necrosis factor-inducible gene 6 (TSG-6), galectin-1, prostaglandin E2 (PGE2), and indoleamine 2,3-dioxygenase (IDO) [23–27]. Regarding the immunogenicity results of our studies, the values of TNF-α, CD4, and CD8 values were normal or abnormal but NCS. Therefore, the safety profile in present studies was similar to other human trials used adipose-derived stem cells for KOA treatment [28].

Compared to the HA group and 16 M group, 32 M and 64 M groups seemed to have a larger percentage of general disorder and administration site conditions including pain and swelling at the injection site of the joint. The limited joint space, higher volume of injection (4 or 8 ml) may be the reason for those phenomena. Meanwhile, it could be assumed that a proportion of the cells injected into the joint space have not survived and this phenomenon was more pronounced with higher cell doses. Probably, such dead cells might trigger the inflammatory reaction in knee joint and thus cause pain [29]. Similar observation was found in Gupta’s study, the adverse events, such as knee pain and swelling, were predominant in their higher-dose groups (50, 75, and 150 million cells) [30]. In another recent study, Vega et al. have conducted a study using IA injection of allogeneic bone marrow-derived MSCs (BMSC) (40 M cells suspended in 8 ml of Ringer-Lactate). And the post-implantation pain was reported in 53% to 60% of patients in both the experimental and control groups [31]. Hence, pain and local swelling are the most common post-injection complication in patients after IA injection of MSCs.

The earlier onset and longer duration of pain and symptom control mediated by ELIXCYTE® were observed in this study compared to HA treatment. Allogeneic MSCs derived from adipose tissue and bone marrow have been described with the potential for more logistic convenience than autologous options. For example, the Stempeucel® which is an off-the-shelf ex vivo expanded bone marrow-derived allogeneic MSCs, was studied in phase II randomized trial with 60 patients. A non-statistically significant trend toward improvement in VAS and WOMAC scores compared with placebo was observed at 6 and 12 months [30]. However, MSCs from bone marrow appear to have high propensity to undergo chondrocyte hypertrophy and bone formation and thus may not be ideal for the repair of articular cartilage [32–34]. Adipose tissue is an attractive source of therapeutic MSCs not only owing to less invasive procurement for harvest, but also due to higher MSC concentration than in bone marrow, lesser expression of MHC class I antigens, and greater replicative and secretory potential of MSC. Additionally, ADSCs has higher immunomodulatory capability that is an important property for treating of OA than BMSC [35, 36].

Although adipose tissue-derived stem cells have been reported to aid in cartilage repair as evidenced by arthroscopy and histological indications of hyaline-like cartilage regeneration [14]. It is still challenging to demonstrate a significant therapeutic improvement in cartilage structure by radiographic measures in clinical trials. Moreover, the relationship between the structural recovery of cartilage and the symptom relief of OA remains unclear. The effect of ELIXCYTE® on cartilage regeneration has also been evaluated by magnetic resonance imaging (MRI) in the present study. No cartilage regeneration or structural modification have been identified and that might be due to the small sample size, the insensitivity and variation of radiographic outcomes, and the effects covered by natural disease progression (Additional file 2: Table S11, S12). In addition, the efficacy of ELIXCYTE® was not in a dosage-dependent manner and the optimal dosage and application volume still need to be further explored. For achieving effective and durable therapeutic effect, administration of sufficient amount of MSCs is critical. Repeated dosing of MSCs has demonstrated a superior clinical outcome compared to single dose in previous study [37]. Reaching sufficient cell numbers by giving smaller volumes of repeated doses may be one of the strategies for designing treatments in the further development of ELIXCYTE®.

## Conclusions

In summary, the safety and therapeutic efficacy of allogenic ADSC therapy for treating KOA patients was reported. All ELIXCYTE® groups presented a comparable safety profile to HA along with an earlier onset and longer duration of the effectiveness compared to the HA. The administration of ELIXCYTE® is low risk and these results support the continued development of ELIXCYTE®.

## Supplementary Information

**Additional file 1**: Hematology and biochemistry data from patients in trial.

**Additional file 2**: Supplementary safety and efficacy data of ELIXCYTE® including Table S1-S12.

## Data Availability

The datasets used or analyzed during the current study are available from the corresponding author on reasonable request.

## Abbreviations

ADSCs: Adipose-derived stem cells
IA: Intra-articular
HA: Hyaluronic acid
WOMAC: Western Ontario and McMaster Universities Osteoarthritis Index
AEs: Adverse events
SAEs: Serious adverse events
SUSAR: Suspected unexpected serious adverse reaction
VAS: Visual analog scale
KSCRS: Knee Society Clinical Rating System
KOA: Knee osteoarthritis
YLDs: Years lived with disability
NSAID: Nonsteroidal anti-inflammatory drug
DMOAD: Disease-modifying osteoarthritis drug
MSCs: Mesenchymal stem cells
SVF: Stromal vascular fraction
CRO: Contract research organization
M: Million
ACR: American College of Rheumatology
HIV: Human immunodeficiency virus
BMI: Body mass index
WBC: White blood cells
RBC: Red blood cells
BUN: Blood urea nitrogen
TNF-α: Tumor necrosis factor alpha
ITT: Intent-to-treat
KL: Kellgren–Lawrence
TEAE: Treatment-emergent adverse event
SOC: System organ class
NCS: No clinically significant
AST: Aspartate aminotransferase
MHC: Major histocompatibility complex
TSG-6: Tumor necrosis factor-inducible gene 6, galectin-1
PGE2: Prostaglandin E2
IDO: Indoleamine 2,3-dioxygenase
BMSCs: Bone marrow-derived MSCs
MRI: Magnetic resonance imaging
