# Supplementary material for: Treatment of knee osteoarthritis with intra-articular injection of allogeneic adipose-derived stem cells (ADSCs) ELIXCYTE®: a phase I/II, randomized, active-control, single-blind, multiple-center clinical trial
Source: Stem Cell Res Ther. 2021 Oct 30;12:562. doi: 10.1186/s13287-021-02631-z (PMC8557559; doi:10.1186/s13287-021-02631-z)
Supplement: Supplementary file 1 — Additional file 1: Hematology and biochemistry data from patients in trial. [file 13287_2021_2631_MOESM1_ESM.docx]

**Hematology and biochemistry data from patients in trial.**

**Table 1 Summary of White Blood Cells [10^9^/L] - Safety Population**

| **White Blood Cells (Mean)** | | **HA** | **16M** | **32M** | **64M** | **Pooled** |
| --- | --- | --- | --- | --- | --- | --- |
| **Screening** | N (Missing) | 8 (0) | 17 (0) | 17 (0) | 15 (0) | 49 (0) |
|  | Mean (SD) | 6.20 (2.318) | 7.24 (3.098) | 6.53 (1.848) | 7.00 (1.398) | 6.92 (2.236) |
|  | Groups Diff.  P-value (Wilcox_t) | -- | 0.2020 | 0.3602 | 0.1138 | 0.1408 |
|  | Normal | 7 (87.5%) | 16 (94.1%) | 15 (88.2%) | 15 (100.0%) | 46 (93.9%) |
|  | NCS | 1 (12.5%) | 1 (5.9%) | 2 (11.8%) | 0 (0.0%) | 3 (6.1%) |
|  | CS (MH) | 0 (0.0%) | 0 (0.0%) | 0 (0.0%) | 0 (0.0%) | 0 (0.0%) |
|  | CS (AE) | 0 (0.0%) | 0 (0.0%) | 0 (0.0%) | 0 (0.0%) | 0 (0.0%) |
| **Week 24** | N (Missing) | 8 (0) | 17 (0) | 17 (0) | 15 (0) | 49 (0) |
|  | Mean (SD) | 6.03 (1.187) | 6.29 (1.414) | 6.51 (1.548) | 6.36 (2.066) | 6.39 (1.649) |
|  | Normal | 8 (100.0%) | 16 (94.1%) | 16 (94.1%) | 13 (86.7%) | 45 (91.8%) |
|  | NCS | 0 (0.0%) | 1 (5.9%) | 1 (5.9%) | 2 (13.3%) | 4 (8.2%) |
|  | CS (MH) | 0 (0.0%) | 0 (0.0%) | 0 (0.0%) | 0 (0.0%) | 0 (0.0%) |
|  | CS (AE) | 0 (0.0%) | 0 (0.0%) | 0 (0.0%) | 0 (0.0%) | 0 (0.0%) |
| Transition from baseline | Relieved | 0 (0.0%) | 0 (0.0%) | 0 (0.0%) | 0 (0.0%) | 0 (0.0%) |
|  | Unchanged | 8 (100.0%) | 17 (100.0%) | 17 (100.0%) | 15 (100.0%) | 49 (100.0%) |
|  | Worsened (MH) | 0 (0.0%) | 0 (0.0%) | 0 (0.0%) | 0 (0.0%) | 0 (0.0%) |
|  | Worsened (AE) | 0 (0.0%) | 0 (0.0%) | 0 (0.0%) | 0 (0.0%) | 0 (0.0%) |
| **Week 36** | N (Missing) | 6 (0) | 12 (0) | 13 (0) | 14 (0) | 39 (0) |
|  | Mean (SD) | 6.00 (1.225) | 5.87 (1.052) | 6.98 (1.873) | 6.18 (1.754) | 6.35 (1.644) |
|  | Normal | 6 (100.0%) | 12 (100.0%) | 13 (100.0%) | 13 (92.9%) | 38 (97.4%) |
|  | NCS | 0 (0.0%) | 0 (0.0%) | 0 (0.0%) | 1 (7.1%) | 1 (2.6%) |
|  | CS (MH) | 0 (0.0%) | 0 (0.0%) | 0 (0.0%) | 0 (0.0%) | 0 (0.0%) |
|  | CS (AE) | 0 (0.0%) | 0 (0.0%) | 0 (0.0%) | 0 (0.0%) | 0 (0.0%) |
| Transition from baseline | Relieved | 0 (0.0%) | 0 (0.0%) | 0 (0.0%) | 0 (0.0%) | 0 (0.0%) |
|  | Unchanged | 6 (100.0%) | 12 (100.0%) | 13 (100.0%) | 14 (100.0%) | 39 (100.0%) |
|  | Worsened (MH) | 0 (0.0%) | 0 (0.0%) | 0 (0.0%) | 0 (0.0%) | 0 (0.0%) |
|  | Worsened (AE) | 0 (0.0%) | 0 (0.0%) | 0 (0.0%) | 0 (0.0%) | 0 (0.0%) |
| **Week 48** | N (Missing) | 6 (0) | 12 (0) | 13 (0) | 12 (0) | 37 (0) |
|  | Mean (SD) | 6.33 (0.983) | 6.35 (1.525) | 7.11 (1.493) | 6.33 (1.381) | 6.61 (1.475) |
|  | Normal | 6 (100.0%) | 11 (91.7%) | 13 (100.0%) | 12 (100.0%) | 36 (97.3%) |
|  | NCS | 0 (0.0%) | 1 (8.3%) | 0 (0.0%) | 0 (0.0%) | 1 (2.7%) |
|  | CS (MH) | 0 (0.0%) | 0 (0.0%) | 0 (0.0%) | 0 (0.0%) | 0 (0.0%) |
|  | CS (AE) | 0 (0.0%) | 0 (0.0%) | 0 (0.0%) | 0 (0.0%) | 0 (0.0%) |
| Transition from baseline | Relieved | 0 (0.0%) | 0 (0.0%) | 0 (0.0%) | 0 (0.0%) | 0 (0.0%) |
|  | Unchanged | 6 (100.0%) | 12 (100.0%) | 13 (100.0%) | 12 (100.0%) | 37 (100.0%) |
|  | Worsened (MH) | 0 (0.0%) | 0 (0.0%) | 0 (0.0%) | 0 (0.0%) | 0 (0.0%) |
|  | Worsened (AE) | 0 (0.0%) | 0 (0.0%) | 0 (0.0%) | 0 (0.0%) | 0 (0.0%) |
| **Week 72** | N (Missing) | 3 (0) | 6 (0) | 7 (0) | 5 (0) | 18 (0) |
|  | Mean (SD) | 5.13 (1.286) | 5.65 (0.957) | 5.97 (1.293) | 6.14 (2.284) | 5.91 (1.459) |
|  | Normal | 3 (100.0%) | 6 (100.0%) | 7 (100.0%) | 3 (60.0%) | 16 (88.9%) |
|  | NCS | 0 (0.0%) | 0 (0.0%) | 0 (0.0%) | 2 (40.0%) | 2 (11.1%) |
|  | CS (MH) | 0 (0.0%) | 0 (0.0%) | 0 (0.0%) | 0 (0.0%) | 0 (0.0%) |
|  | CS (AE) | 0 (0.0%) | 0 (0.0%) | 0 (0.0%) | 0 (0.0%) | 0 (0.0%) |
| Transition from baseline | Relieved | 0 (0.0%) | 0 (0.0%) | 0 (0.0%) | 0 (0.0%) | 0 (0.0%) |
|  | Unchanged | 3 (100.0%) | 6 (100.0%) | 7 (100.0%) | 5 (100.0%) | 18 (100.0%) |
|  | Worsened (MH) | 0 (0.0%) | 0 (0.0%) | 0 (0.0%) | 0 (0.0%) | 0 (0.0%) |
|  | Worsened (AE) | 0 (0.0%) | 0 (0.0%) | 0 (0.0%) | 0 (0.0%) | 0 (0.0%) |
| **Week 96** | N (Missing) | 3 (0) | 7 (0) | 10 (0) | 7 (0) | 24 (0) |
|  | Mean (SD) | 5.20 (0.624) | 6.03 (1.176) | 7.22 (2.559) | 5.96 (1.288) | 6.50 (1.934) |
|  | Normal | 3 (100.0%) | 6 (85.7%) | 9 (90.0%) | 6 (85.7%) | 21 (87.5%) |
|  | NCS | 0 (0.0%) | 1 (14.3%) | 1 (10.0%) | 1 (14.3%) | 3 (12.5%) |
|  | CS (MH) | 0 (0.0%) | 0 (0.0%) | 0 (0.0%) | 0 (0.0%) | 0 (0.0%) |
|  | CS (AE) | 0 (0.0%) | 0 (0.0%) | 0 (0.0%) | 0 (0.0%) | 0 (0.0%) |
| Transition from baseline | Relieved | 0 (0.0%) | 0 (0.0%) | 0 (0.0%) | 0 (0.0%) | 0 (0.0%) |
|  | Unchanged | 3 (100.0%) | 7 (100.0%) | 10 (100.0%) | 7 (100.0%) | 24 (100.0%) |
|  | Worsened (MH) | 0 (0.0%) | 0 (0.0%) | 0 (0.0%) | 0 (0.0%) | 0 (0.0%) |
|  | Worsened (AE) | 0 (0.0%) | 0 (0.0%) | 0 (0.0%) | 0 (0.0%) | 0 (0.0%) |

*NCS: abnormal but not clinically significant, MH: Medical history, AE: Adverse event. Relieved: CS (Medical History or Adverse Event) at baseline to Normal / NCS at visit*

*Unchanged: including Normal at baseline to NCS at visit, NCS at baseline to Normal at visit. Worsened (Medical History): Normal / NCS at baseline to CS (Medical History) at visit. Worsened (Adverse Event): Normal / NCS / CS (Medical History) at baseline to CS (Adverse Event) at visit*

**Table 2 Summary of Neutrophils [%] - Safety Population**

| **Neutrophils** | | **HA** | **16M** | **32M** | **64M** | **Pooled** |
| --- | --- | --- | --- | --- | --- | --- |
| **Screening** | N (Missing) | 8 (0) | 17 (0) | 17 (0) | 15 (0) | 49 (0) |
|  | Mean (SD) | 61.14 (7.394) | 61.66 (10.786) | 57.26 (6.971) | 57.33 (8.524) | 58.81 (8.978) |
|  | Groups Diff.  P-value (Wilcox_t) | -- | 0.8177 | 0.3046 | 0.3927 | 0.4052 |
|  | Normal | 8 (100.0%) | 15 (88.2%) | 17 (100.0%) | 15 (100.0%) | 47 (95.9%) |
|  | NCS | 0 (0.0%) | 2 (11.8%) | 0 (0.0%) | 0 (0.0%) | 2 (4.1%) |
|  | CS (MH) | 0 (0.0%) | 0 (0.0%) | 0 (0.0%) | 0 (0.0%) | 0 (0.0%) |
|  | CS (AE) | 0 (0.0%) | 0 (0.0%) | 0 (0.0%) | 0 (0.0%) | 0 (0.0%) |
| **Week 24** | N (Missing) | 8 (0) | 17 (0) | 17 (0) | 15 (0) | 49 (0) |
|  | Mean (SD) | 60.11 (9.746) | 59.69 (8.086) | 55.39 (7.844) | 54.13 (6.834) | 56.50 (7.856) |
|  | Normal | 7 (87.5%) | 16 (94.1%) | 16 (94.1%) | 14 (93.3%) | 46 (93.9%) |
|  | NCS | 1 (12.5%) | 1 (5.9%) | 1 (5.9%) | 1 (6.7%) | 3 (6.1%) |
|  | CS (MH) | 0 (0.0%) | 0 (0.0%) | 0 (0.0%) | 0 (0.0%) | 0 (0.0%) |
|  | CS (AE) | 0 (0.0%) | 0 (0.0%) | 0 (0.0%) | 0 (0.0%) | 0 (0.0%) |
| Transition from baseline | Relieved | 0 (0.0%) | 0 (0.0%) | 0 (0.0%) | 0 (0.0%) | 0 (0.0%) |
|  | Unchanged | 8 (100.0%) | 17 (100.0%) | 17 (100.0%) | 15 (100.0%) | 49 (100.0%) |
|  | Worsened (MH) | 0 (0.0%) | 0 (0.0%) | 0 (0.0%) | 0 (0.0%) | 0 (0.0%) |
|  | Worsened (AE) | 0 (0.0%) | 0 (0.0%) | 0 (0.0%) | 0 (0.0%) | 0 (0.0%) |
| **Week 36** | N (Missing) | 6 (0) | 12 (0) | 13 (0) | 14 (0) | 39 (0) |
|  | Mean (SD) | 59.07 (8.778) | 54.56 (11.406) | 51.62 (6.923) | 54.18 (8.625) | 53.44 (8.943) |
|  | Normal | 6 (100.0%) | 11 (91.7%) | 12 (92.3%) | 12 (85.7%) | 35 (89.7%) |
|  | NCS | 0 (0.0%) | 1 (8.3%) | 1 (7.7%) | 2 (14.3%) | 4 (10.3%) |
|  | CS (MH) | 0 (0.0%) | 0 (0.0%) | 0 (0.0%) | 0 (0.0%) | 0 (0.0%) |
|  | CS (AE) | 0 (0.0%) | 0 (0.0%) | 0 (0.0%) | 0 (0.0%) | 0 (0.0%) |
| Transition from baseline | Relieved | 0 (0.0%) | 0 (0.0%) | 0 (0.0%) | 0 (0.0%) | 0 (0.0%) |
|  | Unchanged | 6 (100.0%) | 12 (100.0%) | 13 (100.0%) | 14 (100.0%) | 39 (100.0%) |
|  | Worsened (MH) | 0 (0.0%) | 0 (0.0%) | 0 (0.0%) | 0 (0.0%) | 0 (0.0%) |
|  | Worsened (AE) | 0 (0.0%) | 0 (0.0%) | 0 (0.0%) | 0 (0.0%) | 0 (0.0%) |
| **Week 48** | N (Missing) | 6 (0) | 12 (0) | 13 (0) | 12 (0) | 37 (0) |
|  | Mean (SD) | 61.83 (7.630) | 59.46 (6.188) | 56.88 (6.343) | 55.72 (7.686) | 57.34 (6.753) |
|  | Normal | 6 (100.0%) | 12 (100.0%) | 13 (100.0%) | 11 (91.7%) | 36 (97.3%) |
|  | NCS | 0 (0.0%) | 0 (0.0%) | 0 (0.0%) | 1 (8.3%) | 1 (2.7%) |
|  | CS (MH) | 0 (0.0%) | 0 (0.0%) | 0 (0.0%) | 0 (0.0%) | 0 (0.0%) |
|  | CS (AE) | 0 (0.0%) | 0 (0.0%) | 0 (0.0%) | 0 (0.0%) | 0 (0.0%) |
| Transition from baseline | Relieved | 0 (0.0%) | 0 (0.0%) | 0 (0.0%) | 0 (0.0%) | 0 (0.0%) |
|  | Unchanged | 6 (100.0%) | 12 (100.0%) | 13 (100.0%) | 12 (100.0%) | 37 (100.0%) |
|  | Worsened (MH) | 0 (0.0%) | 0 (0.0%) | 0 (0.0%) | 0 (0.0%) | 0 (0.0%) |
|  | Worsened (AE) | 0 (0.0%) | 0 (0.0%) | 0 (0.0%) | 0 (0.0%) | 0 (0.0%) |
| **Week 72** | N (Missing) | 3 (0) | 6 (0) | 7 (0) | 5 (0) | 18 (0) |
|  | Mean (SD) | 60.77 (3.182) | 57.10 (7.340) | 56.63 (5.364) | 47.18 (10.975) | 54.16 (8.616) |
|  | Normal | 3 (100.0%) | 6 (100.0%) | 7 (100.0%) | 4 (80.0%) | 17 (94.4%) |
|  | NCS | 0 (0.0%) | 0 (0.0%) | 0 (0.0%) | 1 (20.0%) | 1 (5.6%) |
|  | CS (MH) | 0 (0.0%) | 0 (0.0%) | 0 (0.0%) | 0 (0.0%) | 0 (0.0%) |
|  | CS (AE) | 0 (0.0%) | 0 (0.0%) | 0 (0.0%) | 0 (0.0%) | 0 (0.0%) |
| Transition from baseline | Relieved | 0 (0.0%) | 0 (0.0%) | 0 (0.0%) | 0 (0.0%) | 0 (0.0%) |
|  | Unchanged | 3 (100.0%) | 6 (100.0%) | 7 (100.0%) | 5 (100.0%) | 18 (100.0%) |
|  | Worsened (MH) | 0 (0.0%) | 0 (0.0%) | 0 (0.0%) | 0 (0.0%) | 0 (0.0%) |
|  | Worsened (AE) | 0 (0.0%) | 0 (0.0%) | 0 (0.0%) | 0 (0.0%) | 0 (0.0%) |
| **Week 96** | N (Missing) | 3 (0) | 7 (0) | 10 (0) | 7 (0) | 24 (0) |
|  | Mean (SD) | 61.67 (4.826) | 55.37 (3.845) | 57.92 (9.171) | 53.54 (7.829) | 55.90 (7.504) |
|  | Normal | 3 (100.0%) | 7 (100.0%) | 9 (90.0%) | 6 (85.7%) | 22 (91.7%) |
|  | NCS | 0 (0.0%) | 0 (0.0%) | 1 (10.0%) | 1 (14.3%) | 2 (8.3%) |
|  | CS (MH) | 0 (0.0%) | 0 (0.0%) | 0 (0.0%) | 0 (0.0%) | 0 (0.0%) |
|  | CS (AE) | 0 (0.0%) | 0 (0.0%) | 0 (0.0%) | 0 (0.0%) | 0 (0.0%) |
| Transition from baseline | Relieved | 0 (0.0%) | 0 (0.0%) | 0 (0.0%) | 0 (0.0%) | 0 (0.0%) |
|  | Unchanged | 3 (100.0%) | 7 (100.0%) | 10 (100.0%) | 7 (100.0%) | 24 (100.0%) |
|  | Worsened (MH) | 0 (0.0%) | 0 (0.0%) | 0 (0.0%) | 0 (0.0%) | 0 (0.0%) |
|  | Worsened (AE) | 0 (0.0%) | 0 (0.0%) | 0 (0.0%) | 0 (0.0%) | 0 (0.0%) |

*NCS: abnormal but not clinically significant, MH: Medical history, AE: Adverse event. Relieved: CS (Medical History or Adverse Event) at baseline to Normal / NCS at visit*

*Unchanged: including Normal at baseline to NCS at visit, NCS at baseline to Normal at visit. Worsened (Medical History): Normal / NCS at baseline to CS (Medical History) at visit. Worsened (Adverse Event): Normal / NCS / CS (Medical History) at baseline to CS (Adverse Event) at visit.*

**Table 3 Summary of Lymphocytes [%] - Safety Population**

| **Lymphocytes** | | **HA** | **16M** | **32M** | **64M** | **Pooled** |
| --- | --- | --- | --- | --- | --- | --- |
| **Screening** | N (Missing) | 8 (0) | 17 (0) | 17 (0) | 15(0) | 49 (0) |
|  | Mean (SD) | 30.10 (6.477) | 29.61 (10.233) | 32.26 (6.862) | 34.18 (8.009) | 31.93 (8.535) |
|  | Groups Diff.  P-value(T test) | -- | 0.9020 | 0.4619 | 0.2297 | 0.5658 |
|  | Normal | 8 (100.0%) | 14 (82.4%) | 17 (100.0%) | 14 (93.3%) | 45 (91.8%) |
|  | NCS | 0 (0.0%) | 3 (17.6%) | 0 (0.0%) | 1 (6.7%) | 4 (8.2%) |
|  | CS (MH) | 0 (0.0%) | 0 (0.0%) | 0 (0.0%) | 0 (0.0%) | 0 (0.0%) |
|  | CS (AE) | 0 (0.0%) | 0 (0.0%) | 0 (0.0%) | 0 (0.0%) | 0 (0.0%) |
| **Week 24** | N (Missing) | 8 (0) | 17 (0) | 17 (0) | 15(0) | 49 (0) |
|  | Mean (SD) | 31.69 (9.229) | 30.42 (8.093) | 34.65 (7.717) | 36.23 (6.602) | 33.67 (7.779) |
|  | Normal | 7 (87.5%) | 16 (94.1%) | 17 (100.0%) | 15 (100.0%) | 48 (98.0%) |
|  | NCS | 1 (12.5%) | 1 (5.9%) | 0 (0.0%) | 0 (0.0%) | 1 (2.0%) |
|  | CS (MH) | 0 (0.0%) | 0 (0.0%) | 0 (0.0%) | 0 (0.0%) | 0 (0.0%) |
|  | CS (AE) | 0 (0.0%) | 0 (0.0%) | 0 (0.0%) | 0 (0.0%) | 0 (0.0%) |
| Transition from baseline | Relieved | 0 (0.0%) | 0 (0.0%) | 0 (0.0%) | 0 (0.0%) | 0 (0.0%) |
|  | Unchanged | 8 (100.0%) | 17 (100.0%) | 17 (100.0%) | 15 (100.0%) | 49 (100.0%) |
|  | Worsened (MH) | 0 (0.0%) | 0 (0.0%) | 0 (0.0%) | 0 (0.0%) | 0 (0.0%) |
|  | Worsened (AE) | 0 (0.0%) | 0 (0.0%) | 0 (0.0%) | 0 (0.0%) | 0 (0.0%) |
| **Week 36** | N (Missing) | 6 (0) | 12 (0) | 13 (0) | 14(0) | 39 (0) |
|  | Mean (SD) | 31.63 (8.545) | 33.68 (7.206) | 38.43 (7.595) | 35.46 (7.094) | 35.90 (7.368) |
|  | Normal | 5 (83.3%) | 11 (91.7%) | 13 (100.0%) | 14 (100.0%) | 38 (97.4%) |
|  | NCS | 1 (16.7%) | 1 (8.3%) | 0 (0.0%) | 0 (0.0%) | 1 (2.6%) |
|  | CS (MH) | 0 (0.0%) | 0 (0.0%) | 0 (0.0%) | 0 (0.0%) | 0 (0.0%) |
|  | CS (AE) | 0 (0.0%) | 0 (0.0%) | 0 (0.0%) | 0 (0.0%) | 0 (0.0%) |
| Transition from baseline | Relieved | 0 (0.0%) | 0 (0.0%) | 0 (0.0%) | 0 (0.0%) | 0 (0.0%) |
|  | Unchanged | 6 (100.0%) | 12 (100.0%) | 13 (100.0%) | 14 (100.0%) | 39 (100.0%) |
|  | Worsened (MH) | 0 (0.0%) | 0 (0.0%) | 0 (0.0%) | 0 (0.0%) | 0 (0.0%) |
|  | Worsened (AE) | 0 (0.0%) | 0 (0.0%) | 0 (0.0%) | 0 (0.0%) | 0 (0.0%) |
| **Week 48** | N (Missing) | 6 (0) | 12 (0) | 13 (0) | 12(0) | 37 (0) |
|  | Mean (SD) | 28.77 (8.302) | 31.36 (6.252) | 33.89 (5.989) | 34.30 (6.272) | 33.20 (6.134) |
|  | Normal | 5 (83.3%) | 12 (100.0%) | 13 (100.0%) | 11 (91.7%) | 36 (97.3%) |
|  | NCS | 1 (16.7%) | 0 (0.0%) | 0 (0.0%) | 1 (8.3%) | 1 (2.7%) |
|  | CS (MH) | 0 (0.0%) | 0 (0.0%) | 0 (0.0%) | 0 (0.0%) | 0 (0.0%) |
|  | CS (AE) | 0 (0.0%) | 0 (0.0%) | 0 (0.0%) | 0 (0.0%) | 0 (0.0%) |
| Transition from baseline | Relieved | 0 (0.0%) | 0 (0.0%) | 0 (0.0%) | 0 (0.0%) | 0 (0.0%) |
|  | Unchanged | 6 (100.0%) | 12 (100.0%) | 13 (100.0%) | 12 (100.0%) | 37 (100.0%) |
|  | Worsened (MH) | 0 (0.0%) | 0 (0.0%) | 0 (0.0%) | 0 (0.0%) | 0 (0.0%) |
|  | Worsened (AE) | 0 (0.0%) | 0 (0.0%) | 0 (0.0%) | 0 (0.0%) | 0 (0.0%) |
| **Week 72** | N (Missing) | 3 (0) | 6 (0) | 7 (0) | 5 (0) | 18 (0) |
|  | Mean (SD) | 28.30 (1.664) | 31.65 (8.799) | 32.44 (5.782) | 42.86 (10.498) | 35.07 (9.237) |
|  | Normal | 3 (100.0%) | 5 (83.3%) | 7 (100.0%) | 4 (80.0%) | 16 (88.9%) |
|  | NCS | 0 (0.0%) | 1 (16.7%) | 0 (0.0%) | 1 (20.0%) | 2 (11.1%) |
|  | CS (MH) | 0 (0.0%) | 0 (0.0%) | 0 (0.0%) | 0 (0.0%) | 0 (0.0%) |
|  | CS (AE) | 0 (0.0%) | 0 (0.0%) | 0 (0.0%) | 0 (0.0%) | 0 (0.0%) |
| Transition from baseline | Relieved | 0 (0.0%) | 0 (0.0%) | 0 (0.0%) | 0 (0.0%) | 0 (0.0%) |
|  | Unchanged | 3 (100.0%) | 6 (100.0%) | 7 (100.0%) | 5 (100.0%) | 18 (100.0%) |
|  | Worsened (MH) | 0 (0.0%) | 0 (0.0%) | 0 (0.0%) | 0 (0.0%) | 0 (0.0%) |
|  | Worsened (AE) | 0 (0.0%) | 0 (0.0%) | 0 (0.0%) | 0 (0.0%) | 0 (0.0%) |
| **Week 96** | N (Missing) | 3 (0) | 7 (0) | 10 (0) | 7 (0) | 24 (0) |
|  | Mean (SD) | 28.60 (3.700) | 31.91 (7.894) | 32.62 (9.096) | 37.63 (5.839) | 33.88 (7.980) |
|  | Normal | 3 (100.0%) | 6 (85.7%) | 9 (90.0%) | 6 (85.7%) | 21 (87.5%) |
|  | NCS | 0 (0.0%) | 1 (14.3%) | 1 (10.0%) | 1 (14.3%) | 3 (12.5%) |
|  | CS (MH) | 0 (0.0%) | 0 (0.0%) | 0 (0.0%) | 0 (0.0%) | 0 (0.0%) |
|  | CS (AE) | 0 (0.0%) | 0 (0.0%) | 0 (0.0%) | 0 (0.0%) | 0 (0.0%) |
| Transition from baseline | Relieved | 0 (0.0%) | 0 (0.0%) | 0 (0.0%) | 0 (0.0%) | 0 (0.0%) |
|  | Unchanged | 3 (100.0%) | 7 (100.0%) | 10 (100.0%) | 7 (100.0%) | 24 (100.0%) |
|  | Worsened (MH) | 0 (0.0%) | 0 (0.0%) | 0 (0.0%) | 0 (0.0%) | 0 (0.0%) |
|  | Worsened (AE) | 0 (0.0%) | 0 (0.0%) | 0 (0.0%) | 0 (0.0%) | 0 (0.0%) |

*NCS: abnormal but not clinically significant, MH: Medical history, AE: Adverse event. Relieved: CS (Medical History or Adverse Event) at baseline to Normal / NCS at visit*

*Unchanged: including Normal at baseline to NCS at visit, NCS at baseline to Normal at visit. Worsened (Medical History): Normal / NCS at baseline to CS (Medical History) at visit. Worsened (Adverse Event): Normal / NCS / CS (Medical History) at baseline to CS (Adverse Event) at visit.*

**Table 4 Summary of Monocytes [%] - Safety Population**

| **Monocytes** | | **HA** | **16M** | **32M** | **64M** | **Pooled** |
| --- | --- | --- | --- | --- | --- | --- |
| **Screening** | N (Missing) | 8 (0) | 17 (0) | 17 (0) | 15 (0) | 49 (0) |
|  | Mean (SD) | 6.33 (2.310) | 6.18 (2.677) | 7.52 (1.883) | 5.89 (1.869) | 6.56 (2.259) |
|  | Group Diff.  P-value (T test) | -- | 0.8982 | 0.1822 | 0.6316 | 0.7892 |
|  | Normal | 6 (75.0%) | 15 (88.2%) | 13 (76.5%) | 14 (93.3%) | 42 (85.7%) |
|  | NCS | 2 (25.0%) | 2 (11.8%) | 4 (23.5%) | 1 (6.7%) | 7 (14.3%) |
|  | CS (MH) | 0 (0.0%) | 0 (0.0%) | 0 (0.0%) | 0 (0.0%) | 0 (0.0%) |
|  | CS (AE) | 0 (0.0%) | 0 (0.0%) | 0 (0.0%) | 0 (0.0%) | 0 (0.0%) |
| **Week 24** | N (Missing) | 8 (0) | 17 (0) | 17 (0) | 15 (0) | 49 (0) |
|  | Mean (SD) | 5.45 (1.426) | 6.78 (2.301) | 6.94 (2.157) | 6.64 (1.707) | 6.79 (2.044) |
|  | Normal | 8 (100.0%) | 13 (76.5%) | 15 (88.2%) | 13 (86.7%) | 41 (83.7%) |
|  | NCS | 0 (0.0%) | 4 (23.5%) | 2 (11.8%) | 2 (13.3%) | 8 (16.3%) |
|  | CS (MH) | 0 (0.0%) | 0 (0.0%) | 0 (0.0%) | 0 (0.0%) | 0 (0.0%) |
|  | CS (AE) | 0 (0.0%) | 0 (0.0%) | 0 (0.0%) | 0 (0.0%) | 0 (0.0%) |
| Transition from baseline | Relieved | 0 (0.0%) | 0 (0.0%) | 0 (0.0%) | 0 (0.0%) | 0 (0.0%) |
|  | Unchanged | 8 (100.0%) | 17 (100.0%) | 17 (100.0%) | 15 (100.0%) | 49 (100.0%) |
|  | Worsened (MH) | 0 (0.0%) | 0 (0.0%) | 0 (0.0%) | 0 (0.0%) | 0 (0.0%) |
|  | Worsened (AE) | 0 (0.0%) | 0 (0.0%) | 0 (0.0%) | 0 (0.0%) | 0 (0.0%) |
| **Week 36** | N (Missing) | 6 (0) | 12 (0) | 13 (0) | 14 (0) | 39 (0) |
|  | Mean (SD) | 6.90 (0.932) | 7.25 (2.462) | 7.01 (2.233) | 7.53 (2.731) | 7.27 (2.435) |
|  | Normal | 6 (100.0%) | 10 (83.3%) | 11 (84.6%) | 13 (92.9%) | 34 (87.2%) |
|  | NCS | 0 (0.0%) | 2 (16.7%) | 2 (15.4%) | 1 (7.1%) | 5 (12.8%) |
|  | CS (MH) | 0 (0.0%) | 0 (0.0%) | 0 (0.0%) | 0 (0.0%) | 0 (0.0%) |
|  | CS (AE) | 0 (0.0%) | 0 (0.0%) | 0 (0.0%) | 0 (0.0%) | 0 (0.0%) |
| Transition from baseline | Relieved | 0 (0.0%) | 0 (0.0%) | 0 (0.0%) | 0 (0.0%) | 0 (0.0%) |
|  | Unchanged | 6 (100.0%) | 12 (100.0%) | 13 (100.0%) | 14 (100.0%) | 39 (100.0%) |
|  | Worsened (MH) | 0 (0.0%) | 0 (0.0%) | 0 (0.0%) | 0 (0.0%) | 0 (0.0%) |
|  | Worsened (AE) | 0 (0.0%) | 0 (0.0%) | 0 (0.0%) | 0 (0.0%) | 0 (0.0%) |
| **Week 48** | N (Missing) | 6 (0) | 12 (0) | 13 (0) | 12 (0) | 37 (0) |
|  | Mean (SD) | 7.03 (1.089) | 6.67 (1.680) | 6.68 (2.585) | 7.00 (2.373) | 6.78 (2.198) |
|  | Normal | 6 (100.0%) | 11 (91.7%) | 9 (69.2%) | 10 (83.3%) | 30 (81.1%) |
|  | NCS | 0 (0.0%) | 1 (8.3%) | 4 (30.8%) | 2 (16.7%) | 7 (18.9%) |
|  | CS (MH) | 0 (0.0%) | 0 (0.0%) | 0 (0.0%) | 0 (0.0%) | 0 (0.0%) |
|  | CS (AE) | 0 (0.0%) | 0 (0.0%) | 0 (0.0%) | 0 (0.0%) | 0 (0.0%) |
| Transition from baseline | Relieved | 0 (0.0%) | 0 (0.0%) | 0 (0.0%) | 0 (0.0%) | 0 (0.0%) |
|  | Unchanged | 6 (100.0%) | 12 (100.0%) | 13 (100.0%) | 12 (100.0%) | 37 (100.0%) |
|  | Worsened (MH) | 0 (0.0%) | 0 (0.0%) | 0 (0.0%) | 0 (0.0%) | 0 (0.0%) |
|  | Worsened (AE) | 0 (0.0%) | 0 (0.0%) | 0 (0.0%) | 0 (0.0%) | 0 (0.0%) |
| **Week 72** | N (Missing) | 3 (0) | 6 (0) | 7 (0) | 5 (0) | 18 (0) |
|  | Mean (SD) | 7.63 (0.321) | 8.18 (2.478) | 8.39 (2.909) | 7.44 (2.008) | 8.06 (2.430) |
|  | Normal | 3 (100.0%) | 5 (83.3%) | 5 (71.4%) | 4 (80.0%) | 14 (77.8%) |
|  | NCS | 0 (0.0%) | 1 (16.7%) | 2 (28.6%) | 1 (20.0%) | 4 (22.2%) |
|  | CS (MH) | 0 (0.0%) | 0 (0.0%) | 0 (0.0%) | 0 (0.0%) | 0 (0.0%) |
|  | CS (AE) | 0 (0.0%) | 0 (0.0%) | 0 (0.0%) | 0 (0.0%) | 0 (0.0%) |
| Transition from baseline | Relieved | 0 (0.0%) | 0 (0.0%) | 0 (0.0%) | 0 (0.0%) | 0 (0.0%) |
|  | Unchanged | 3 (100.0%) | 6 (100.0%) | 7 (100.0%) | 5 (100.0%) | 18 (100.0%) |
|  | Worsened (MH) | 0 (0.0%) | 0 (0.0%) | 0 (0.0%) | 0 (0.0%) | 0 (0.0%) |
|  | Worsened (AE) | 0 (0.0%) | 0 (0.0%) | 0 (0.0%) | 0 (0.0%) | 0 (0.0%) |
| **Week 96** | N (Missing) | 3 (0) | 7 (0) | 10 (0) | 7 (0) | 24 (0) |
|  | Mean (SD) | 7.17 (0.777) | 8.09 (2.170) | 6.98 (1.935) | 6.71 (3.075) | 7.22 (2.343) |
|  | Normal | 3 (100.0%) | 5 (71.4%) | 8 (80.0%) | 6 (85.7%) | 19 (79.2%) |
|  | NCS | 0 (0.0%) | 2 (28.6%) | 2 (20.0%) | 1 (14.3%) | 5 (20.8%) |
|  | CS (MH) | 0 (0.0%) | 0 (0.0%) | 0 (0.0%) | 0 (0.0%) | 0 (0.0%) |
|  | CS (AE) | 0 (0.0%) | 0 (0.0%) | 0 (0.0%) | 0 (0.0%) | 0 (0.0%) |
| Transition from baseline | Relieved | 0 (0.0%) | 0 (0.0%) | 0 (0.0%) | 0 (0.0%) | 0 (0.0%) |
|  | Unchanged | 3 (100.0%) | 7 (100.0%) | 10 (100.0%) | 7 (100.0%) | 24 (100.0%) |
|  | Worsened (MH) | 0 (0.0%) | 0 (0.0%) | 0 (0.0%) | 0 (0.0%) | 0 (0.0%) |
|  | Worsened (AE) | 0 (0.0%) | 0 (0.0%) | 0 (0.0%) | 0 (0.0%) | 0 (0.0%) |

*NCS: abnormal but not clinically significant, MH: Medical history, AE: Adverse event. Relieved: CS (Medical History or Adverse Event) at baseline to Normal / NCS at visit*

*Unchanged: including Normal at baseline to NCS at visit, NCS at baseline to Normal at visit. Worsened (Medical History): Normal / NCS at baseline to CS (Medical History) at visit. Worsened (Adverse Event): Normal / NCS / CS (Medical History) at baseline to CS (Adverse Event) at visit.*

**Table 5 Summary of Eosinophil [%] - Safety Population**

| **Eosinophils** | | **HA** | **16M** | **32M** | **64M** | **Pooled** |
| --- | --- | --- | --- | --- | --- | --- |
| **Screening** | N (Missing) | 8 (0) | 17 (0) | 17 (0) | 15 (0) | 49 (0) |
|  | Mean (SD) | 1.91 (0.851) | 2.04 (1.236) | 2.29 (1.501) | 2.19 (1.407) | 2.18 (1.360) |
|  | Group Diff.  P-value (Wilcox_t) | -- | 0.8175 | 0.6043 | 0.7492 | 0.6639 |
|  | Normal | 8 (100.0%) | 17 (100.0%) | 16 (94.1%) | 14 (93.3%) | 47 (95.9%) |
|  | NCS | 0 (0.0%) | 0 (0.0%) | 1 (5.9%) | 1 (6.7%) | 2 (4.1%) |
|  | CS (MH) | 0 (0.0%) | 0 (0.0%) | 0 (0.0%) | 0 (0.0%) | 0 (0.0%) |
|  | CS (AE) | 0 (0.0%) | 0 (0.0%) | 0 (0.0%) | 0 (0.0%) | 0 (0.0%) |
| **Week 24** | N (Missing) | 8 (0) | 17 (0) | 17 (0) | 15 (0) | 49 (0) |
|  | Mean (SD) | 2.15 (1.744) | 2.52 (1.052) | 2.32 (1.069) | 2.42 (1.997) | 2.42 (1.386) |
|  | Normal | 8 (100.0%) | 17 (100.0%) | 17 (100.0%) | 14 (93.3%) | 48 (98.0%) |
|  | NCS | 0 (0.0%) | 0 (0.0%) | 0 (0.0%) | 1 (6.7%) | 1 (2.0%) |
|  | CS (MH) | 0 (0.0%) | 0 (0.0%) | 0 (0.0%) | 0 (0.0%) | 0 (0.0%) |
|  | CS (AE) | 0 (0.0%) | 0 (0.0%) | 0 (0.0%) | 0 (0.0%) | 0 (0.0%) |
| Transition from baseline | Relieved | 0 (0.0%) | 0 (0.0%) | 0 (0.0%) | 0 (0.0%) | 0 (0.0%) |
|  | Unchanged | 8 (100.0%) | 17 (100.0%) | 17 (100.0%) | 15 (100.0%) | 49 (100.0%) |
|  | Worsened (MH) | 0 (0.0%) | 0 (0.0%) | 0 (0.0%) | 0 (0.0%) | 0 (0.0%) |
|  | Worsened (AE) | 0 (0.0%) | 0 (0.0%) | 0 (0.0%) | 0 (0.0%) | 0 (0.0%) |
| **Week 36** | N (Missing) | 6 (0) | 12 (0) | 13 (0) | 14 (0) | 39 (0) |
|  | Mean (SD) | 1.72 (0.733) | 2.86 (1.568) | 2.18 (1.260) | 2.23 (1.615) | 2.41 (1.483) |
|  | Normal | 6 (100.0%) | 11 (91.7%) | 13 (100.0%) | 13 (92.9%) | 37 (94.9%) |
|  | NCS | 0 (0.0%) | 1 (8.3%) | 0 (0.0%) | 1 (7.1%) | 2 (5.1%) |
|  | CS (MH) | 0 (0.0%) | 0 (0.0%) | 0 (0.0%) | 0 (0.0%) | 0 (0.0%) |
|  | CS (AE) | 0 (0.0%) | 0 (0.0%) | 0 (0.0%) | 0 (0.0%) | 0 (0.0%) |
| Transition from baseline | Relieved | 0 (0.0%) | 0 (0.0%) | 0 (0.0%) | 0 (0.0%) | 0 (0.0%) |
|  | Unchanged | 6 (100.0%) | 12 (100.0%) | 13 (100.0%) | 14 (100.0%) | 39 (100.0%) |
|  | Worsened (MH) | 0 (0.0%) | 0 (0.0%) | 0 (0.0%) | 0 (0.0%) | 0 (0.0%) |
|  | Worsened (AE) | 0 (0.0%) | 0 (0.0%) | 0 (0.0%) | 0 (0.0%) | 0 (0.0%) |
| **Week 48** | N (Missing) | 6 (0) | 12 (0) | 13 (0) | 12 (0) | 37 (0) |
|  | Mean (SD) | 1.82 (1.057) | 2.00 (0.906) | 1.98 (1.152) | 2.33 (1.457) | 2.10 (1.170) |
|  | Normal | 6 (100.0%) | 12 (100.0%) | 13 (100.0%) | 11 (91.7%) | 36 (97.3%) |
|  | NCS | 0 (0.0%) | 0 (0.0%) | 0 (0.0%) | 1 (8.3%) | 1 (2.7%) |
|  | CS (MH) | 0 (0.0%) | 0 (0.0%) | 0 (0.0%) | 0 (0.0%) | 0 (0.0%) |
|  | CS (AE) | 0 (0.0%) | 0 (0.0%) | 0 (0.0%) | 0 (0.0%) | 0 (0.0%) |
| Transition from baseline | Relieved | 0 (0.0%) | 0 (0.0%) | 0 (0.0%) | 0 (0.0%) | 0 (0.0%) |
|  | Unchanged | 6 (100.0%) | 12 (100.0%) | 13 (100.0%) | 12 (100.0%) | 37 (100.0%) |
|  | Worsened (MH) | 0 (0.0%) | 0 (0.0%) | 0 (0.0%) | 0 (0.0%) | 0 (0.0%) |
|  | Worsened (AE) | 0 (0.0%) | 0 (0.0%) | 0 (0.0%) | 0 (0.0%) | 0 (0.0%) |
| **Week 72** | N (Missing) | 3 (0) | 6 (0) | 7 (0) | 5 (0) | 18 (0) |
|  | Mean (SD) | 2.47 (0.907) | 2.47 (1.213) | 1.86 (1.020) | 1.98 (0.850) | 2.09 (1.022) |
|  | Normal | 3 (100.0%) | 6 (100.0%) | 7 (100.0%) | 5 (100.0%) | 18 (100.0%) |
|  | NCS | 0 (0.0%) | 0 (0.0%) | 0 (0.0%) | 0 (0.0%) | 0 (0.0%) |
|  | CS (MH) | 0 (0.0%) | 0 (0.0%) | 0 (0.0%) | 0 (0.0%) | 0 (0.0%) |
|  | CS (AE) | 0 (0.0%) | 0 (0.0%) | 0 (0.0%) | 0 (0.0%) | 0 (0.0%) |
| Transition from baseline | Relieved | 0 (0.0%) | 0 (0.0%) | 0 (0.0%) | 0 (0.0%) | 0 (0.0%) |
|  | Unchanged | 3 (100.0%) | 6 (100.0%) | 7 (100.0%) | 5 (100.0%) | 18 (100.0%) |
|  | Worsened (MH) | 0 (0.0%) | 0 (0.0%) | 0 (0.0%) | 0 (0.0%) | 0 (0.0%) |
|  | Worsened (AE) | 0 (0.0%) | 0 (0.0%) | 0 (0.0%) | 0 (0.0%) | 0 (0.0%) |
| **Week 96** | N (Missing) | 3 (0) | 7 (0) | 10 (0) | 7 (0) | 24 (0) |
|  | Mean (SD) | 1.77 (0.666) | 4.01 (4.313) | 1.92 (1.032) | 1.67 (0.695) | 2.46 (2.539) |
|  | Normal | 3 (100.0%) | 6 (85.7%) | 10 (100.0%) | 7 (100.0%) | 23 (95.8%) |
|  | NCS | 0 (0.0%) | 1 (14.3%) | 0 (0.0%) | 0 (0.0%) | 1 (4.2%) |
|  | CS (MH) | 0 (0.0%) | 0 (0.0%) | 0 (0.0%) | 0 (0.0%) | 0 (0.0%) |
|  | CS (AE) | 0 (0.0%) | 0 (0.0%) | 0 (0.0%) | 0 (0.0%) | 0 (0.0%) |
| Transition from baseline | Relieved | 0 (0.0%) | 0 (0.0%) | 0 (0.0%) | 0 (0.0%) | 0 (0.0%) |
|  | Unchanged | 3 (100.0%) | 7 (100.0%) | 10 (100.0%) | 7 (100.0%) | 24 (100.0%) |
|  | Worsened (MH) | 0 (0.0%) | 0 (0.0%) | 0 (0.0%) | 0 (0.0%) | 0 (0.0%) |
|  | Worsened (AE) | 0 (0.0%) | 0 (0.0%) | 0 (0.0%) | 0 (0.0%) | 0 (0.0%) |

*NCS: abnormal but not clinically significant, MH: Medical history, AE: Adverse event. Relieved: CS (Medical History or Adverse Event) at baseline to Normal / NCS at visit*

*Unchanged: including Normal at baseline to NCS at visit, NCS at baseline to Normal at visit. Worsened (Medical History): Normal / NCS at baseline to CS (Medical History) at visit. Worsened (Adverse Event): Normal / NCS / CS (Medical History) at baseline to CS (Adverse Event) at visit.*

**Table 6 Summary of Basophil [%] - Safety Population**

| **Basophils** | | **HA** | **16M** | **32M** | **64M** | **Pooled** |
| --- | --- | --- | --- | --- | --- | --- |
| **Screening** | N (Missing) | 8 (0) | 17 (0) | 17 (0) | 15 (0) | 49 (0) |
|  | Mean (SD) | 0.53 (0.440) | 0.51 (0.391) | 0.55 (0.322) | 0.41 (0.191) | 0.49 (0.316) |
|  | Group Diff.  P-value (Wilcox_t) | -- | 1.0000 | 0.7282 | 0.8218 | 0.9724 |
|  | Normal | 7 (87.5%) | 16 (94.1%) | 16 (94.1%) | 15 (100.0%) | 47 (95.9%) |
|  | NCS | 1 (12.5%) | 1 (5.9%) | 1 (5.9%) | 0 (0.0%) | 2 (4.1%) |
|  | CS (MH) | 0 (0.0%) | 0 (0.0%) | 0 (0.0%) | 0 (0.0%) | 0 (0.0%) |
|  | CS (AE) | 0 (0.0%) | 0 (0.0%) | 0 (0.0%) | 0 (0.0%) | 0 (0.0%) |
| **Week 24** | N (Missing) | 8 (0) | 17 (0) | 17 (0) | 15 (0) | 49 (0) |
|  | Mean (SD) | 0.60 (0.499) | 0.58 (0.414) | 0.58 (0.300) | 0.45 (0.267) | 0.54 (0.334) |
|  | Normal | 6 (75.0%) | 14 (82.4%) | 16 (94.1%) | 15 (100.0%) | 45 (91.8%) |
|  | NCS | 2 (25.0%) | 3 (17.6%) | 1 (5.9%) | 0 (0.0%) | 4 (8.2%) |
|  | CS (MH) | 0 (0.0%) | 0 (0.0%) | 0 (0.0%) | 0 (0.0%) | 0 (0.0%) |
|  | CS (AE) | 0 (0.0%) | 0 (0.0%) | 0 (0.0%) | 0 (0.0%) | 0 (0.0%) |
| Transition from baseline | Relieved | 0 (0.0%) | 0 (0.0%) | 0 (0.0%) | 0 (0.0%) | 0 (0.0%) |
|  | Unchanged | 8 (100.0%) | 17 (100.0%) | 17 (100.0%) | 15 (100.0%) | 49 (100.0%) |
|  | Worsened (MH) | 0 (0.0%) | 0 (0.0%) | 0 (0.0%) | 0 (0.0%) | 0 (0.0%) |
|  | Worsened (AE) | 0 (0.0%) | 0 (0.0%) | 0 (0.0%) | 0 (0.0%) | 0 (0.0%) |
| **Week 36** | N (Missing) | 6 (0) | 12 (0) | 13 (0) | 14 (0) | 39 (0) |
|  | Mean (SD) | 0.68 (0.371) | 0.48 (0.327) | 0.75 (0.474) | 0.61 (0.281) | 0.62 (0.375) |
|  | Normal | 4 (66.7%) | 11 (91.7%) | 11 (84.6%) | 13 (92.9%) | 35 (89.7%) |
|  | NCS | 2 (33.3%) | 1 (8.3%) | 2 (15.4%) | 1 (7.1%) | 4 (10.3%) |
|  | CS (MH) | 0 (0.0%) | 0 (0.0%) | 0 (0.0%) | 0 (0.0%) | 0 (0.0%) |
|  | CS (AE) | 0 (0.0%) | 0 (0.0%) | 0 (0.0%) | 0 (0.0%) | 0 (0.0%) |
| Transition from baseline | Relieved | 0 (0.0%) | 0 (0.0%) | 0 (0.0%) | 0 (0.0%) | 0 (0.0%) |
|  | Unchanged | 6 (100.0%) | 12 (100.0%) | 13 (100.0%) | 14 (100.0%) | 39 (100.0%) |
|  | Worsened (MH) | 0 (0.0%) | 0 (0.0%) | 0 (0.0%) | 0 (0.0%) | 0 (0.0%) |
|  | Worsened (AE) | 0 (0.0%) | 0 (0.0%) | 0 (0.0%) | 0 (0.0%) | 0 (0.0%) |
| **Week 48** | N (Missing) | 6 (0) | 12 (0) | 13 (0) | 12 (0) | 37 (0) |
|  | Mean (SD) | 0.55 (0.259) | 0.52 (0.229) | 0.55 (0.357) | 0.65 (0.329) | 0.57 (0.308) |
|  | Normal | 6 (100.0%) | 12 (100.0%) | 12 (92.3%) | 10 (83.3%) | 34 (91.9%) |
|  | NCS | 0 (0.0%) | 0 (0.0%) | 1 (7.7%) | 2 (16.7%) | 3 (8.1%) |
|  | CS (MH) | 0 (0.0%) | 0 (0.0%) | 0 (0.0%) | 0 (0.0%) | 0 (0.0%) |
|  | CS (AE) | 0 (0.0%) | 0 (0.0%) | 0 (0.0%) | 0 (0.0%) | 0 (0.0%) |
| Transition from baseline | Relieved | 0 (0.0%) | 0 (0.0%) | 0 (0.0%) | 0 (0.0%) | 0 (0.0%) |
|  | Unchanged | 6 (100.0%) | 12 (100.0%) | 13 (100.0%) | 12 (100.0%) | 37 (100.0%) |
|  | Worsened (MH) | 0 (0.0%) | 0 (0.0%) | 0 (0.0%) | 0 (0.0%) | 0 (0.0%) |
|  | Worsened (AE) | 0 (0.0%) | 0 (0.0%) | 0 (0.0%) | 0 (0.0%) | 0 (0.0%) |
| **Week 72** | N (Missing) | 3 (0) | 6 (0) | 7 (0) | 5 (0) | 18 (0) |
|  | Mean (SD) | 0.83 (0.351) | 0.60 (0.363) | 0.69 (0.267) | 0.54 (0.251) | 0.62 (0.287) |
|  | Normal | 2 (66.7%) | 5 (83.3%) | 6 (85.7%) | 5 (100.0%) | 16 (88.9%) |
|  | NCS | 1 (33.3%) | 1 (16.7%) | 1 (14.3%) | 0 (0.0%) | 2 (11.1%) |
|  | CS (MH) | 0 (0.0%) | 0 (0.0%) | 0 (0.0%) | 0 (0.0%) | 0 (0.0%) |
|  | CS (AE) | 0 (0.0%) | 0 (0.0%) | 0 (0.0%) | 0 (0.0%) | 0 (0.0%) |
| Transition from baseline | Relieved | 0 (0.0%) | 0 (0.0%) | 0 (0.0%) | 0 (0.0%) | 0 (0.0%) |
|  | Unchanged | 3 (100.0%) | 6 (100.0%) | 7 (100.0%) | 5 (100.0%) | 18 (100.0%) |
|  | Worsened (MH) | 0 (0.0%) | 0 (0.0%) | 0 (0.0%) | 0 (0.0%) | 0 (0.0%) |
|  | Worsened (AE) | 0 (0.0%) | 0 (0.0%) | 0 (0.0%) | 0 (0.0%) | 0 (0.0%) |
| **Week 96** | N (Missing) | 3 (0) | 7 (0) | 10 (0) | 7 (0) | 24 (0) |
|  | Mean (SD) | 0.80 (0.600) | 0.61 (0.285) | 0.56 (0.317) | 0.44 (0.237) | 0.54 (0.283) |
|  | Normal | 2 (66.7%) | 6 (85.7%) | 9 (90.0%) | 7 (100.0%) | 22 (91.7%) |
|  | NCS | 1 (33.3%) | 1 (14.3%) | 1 (10.0%) | 0 (0.0%) | 2 (8.3%) |
|  | CS (MH) | 0 (0.0%) | 0 (0.0%) | 0 (0.0%) | 0 (0.0%) | 0 (0.0%) |
|  | CS (AE) | 0 (0.0%) | 0 (0.0%) | 0 (0.0%) | 0 (0.0%) | 0 (0.0%) |
| Transition from baseline | Relieved | 0 (0.0%) | 0 (0.0%) | 0 (0.0%) | 0 (0.0%) | 0 (0.0%) |
|  | Unchanged | 3 (100.0%) | 7 (100.0%) | 10 (100.0%) | 7 (100.0%) | 24 (100.0%) |
|  | Worsened (MH) | 0 (0.0%) | 0 (0.0%) | 0 (0.0%) | 0 (0.0%) | 0 (0.0%) |
|  | Worsened (AE) | 0 (0.0%) | 0 (0.0%) | 0 (0.0%) | 0 (0.0%) | 0 (0.0%) |

*NCS: abnormal but not clinically significant, MH: Medical history, AE: Adverse event. Relieved: CS (Medical History or Adverse Event) at baseline to Normal / NCS at visit*

*Unchanged: including Normal at baseline to NCS at visit, NCS at baseline to Normal at visit. Worsened (Medical History): Normal / NCS at baseline to CS (Medical History) at visit. Worsened (Adverse Event): Normal / NCS / CS (Medical History) at baseline to CS (Adverse Event) at visit.*

**Table 7 Summary of Hemoglobin [g/dL] - Safety Population**

| **Hemoglobin** | | **HA** | **16M** | **32M** | **64M** | **Pooled** |
| --- | --- | --- | --- | --- | --- | --- |
| **Screening** | N (Missing) | 8 (0) | 17 (0) | 17 (0) | 15 (0) | 49 (0) |
|  | Mean (SD) | 12.25 (0.912) | 13.86 (0.898) | 12.98 (1.239) | 13.35 (1.237) | 13.40 (1.169) |
|  | Groups Diff.  P-value (T test) | -- | 0.0004 | 0.1508 | 0.0382 | 0.0104 |
|  | Normal | 2 (25.0%) | 16 (94.1%) | 14 (82.4%) | 13 (86.7%) | 43 (87.8%) |
|  | NCS | 6 (75.0%) | 1 (5.9%) | 3 (17.6%) | 2 (13.3%) | 6 (12.2%) |
|  | CS (MH) | 0 (0.0%) | 0 (0.0%) | 0 (0.0%) | 0 (0.0%) | 0 (0.0%) |
|  | CS (AE) | 0 (0.0%) | 0 (0.0%) | 0 (0.0%) | 0 (0.0%) | 0 (0.0%) |
| **Week 24** | N (Missing) | 8 (0) | 17 (0) | 17 (0) | 15 (0) | 49 (0) |
|  | Mean (SD) | 12.46 (1.426) | 13.75 (1.131) | 13.19 (1.338) | 13.35 (1.121) | 13.43 (1.204) |
|  | Normal | 3 (37.5%) | 17 (100.0%) | 14 (82.4%) | 14 (93.3%) | 45 (91.8%) |
|  | NCS | 5 (62.5%) | 0 (0.0%) | 3 (17.6%) | 1 (6.7%) | 4 (8.2%) |
|  | CS (MH) | 0 (0.0%) | 0 (0.0%) | 0 (0.0%) | 0 (0.0%) | 0 (0.0%) |
|  | CS (AE) | 0 (0.0%) | 0 (0.0%) | 0 (0.0%) | 0 (0.0%) | 0 (0.0%) |
| Transition from baseline | Relieved | 0 (0.0%) | 0 (0.0%) | 0 (0.0%) | 0 (0.0%) | 0 (0.0%) |
|  | Unchanged | 8 (100.0%) | 17 (100.0%) | 17 (100.0%) | 15 (100.0%) | 49 (100.0%) |
|  | Worsened (MH) | 0 (0.0%) | 0 (0.0%) | 0 (0.0%) | 0 (0.0%) | 0 (0.0%) |
|  | Worsened (AE) | 0 (0.0%) | 0 (0.0%) | 0 (0.0%) | 0 (0.0%) | 0 (0.0%) |
| **Week 36** | N (Missing) | 6 (0) | 12 (0) | 13 (0) | 14 (0) | 39 (0) |
|  | Mean (SD) | 12.60 (1.200) | 13.53 (0.770) | 13.58 (1.006) | 13.24 (1.115) | 13.44 (0.969) |
|  | Normal | 4 (66.7%) | 12 (100.0%) | 12 (92.3%) | 13 (92.9%) | 37 (94.9%) |
|  | NCS | 2 (33.3%) | 0 (0.0%) | 1 (7.7%) | 1 (7.1%) | 2 (5.1%) |
|  | CS (MH) | 0 (0.0%) | 0 (0.0%) | 0 (0.0%) | 0 (0.0%) | 0 (0.0%) |
|  | CS (AE) | 0 (0.0%) | 0 (0.0%) | 0 (0.0%) | 0 (0.0%) | 0 (0.0%) |
| Transition from baseline | Relieved | 0 (0.0%) | 0 (0.0%) | 0 (0.0%) | 0 (0.0%) | 0 (0.0%) |
|  | Unchanged | 6 (100.0%) | 12 (100.0%) | 13 (100.0%) | 14 (100.0%) | 39 (100.0%) |
|  | Worsened (MH) | 0 (0.0%) | 0 (0.0%) | 0 (0.0%) | 0 (0.0%) | 0 (0.0%) |
|  | Worsened (AE) | 0 (0.0%) | 0 (0.0%) | 0 (0.0%) | 0 (0.0%) | 0 (0.0%) |
| **Week 48** | N (Missing) | 6 (0) | 12 (0) | 13 (0) | 12 (0) | 37 (0) |
|  | Mean (SD) | 12.70 (1.203) | 13.65 (0.839) | 13.58 (0.960) | 13.35 (1.079) | 13.53 (0.946) |
|  | Normal | 3 (50.0%) | 12 (100.0%) | 12 (92.3%) | 11 (91.7%) | 35 (94.6%) |
|  | NCS | 3 (50.0%) | 0 (0.0%) | 1 (7.7%) | 1 (8.3%) | 2 (5.4%) |
|  | CS (MH) | 0 (0.0%) | 0 (0.0%) | 0 (0.0%) | 0 (0.0%) | 0 (0.0%) |
|  | CS (AE) | 0 (0.0%) | 0 (0.0%) | 0 (0.0%) | 0 (0.0%) | 0 (0.0%) |
| Transition from baseline | Relieved | 0 (0.0%) | 0 (0.0%) | 0 (0.0%) | 0 (0.0%) | 0 (0.0%) |
|  | Unchanged | 6 (100.0%) | 12 (100.0%) | 13 (100.0%) | 12 (100.0%) | 37 (100.0%) |
|  | Worsened (MH) | 0 (0.0%) | 0 (0.0%) | 0 (0.0%) | 0 (0.0%) | 0 (0.0%) |
|  | Worsened (AE) | 0 (0.0%) | 0 (0.0%) | 0 (0.0%) | 0 (0.0%) | 0 (0.0%) |
| **Week 72** | N (Missing) | 3 (0) | 6 (0) | 7 (0) | 5 (0) | 18 (0) |
|  | Mean (SD) | 12.37 (1.168) | 14.05 (1.249) | 13.19 (1.390) | 13.38 (1.117) | 13.53 (1.259) |
|  | Normal | 0 (0.0%) | 6 (100.0%) | 6 (85.7%) | 5 (100.0%) | 17 (94.4%) |
|  | NCS | 3 (100.0%) | 0 (0.0%) | 1 (14.3%) | 0 (0.0%) | 1 (5.6%) |
|  | CS (MH) | 0 (0.0%) | 0 (0.0%) | 0 (0.0%) | 0 (0.0%) | 0 (0.0%) |
|  | CS (AE) | 0 (0.0%) | 0 (0.0%) | 0 (0.0%) | 0 (0.0%) | 0 (0.0%) |
| Transition from baseline | Relieved | 0 (0.0%) | 0 (0.0%) | 0 (0.0%) | 0 (0.0%) | 0 (0.0%) |
|  | Unchanged | 3 (100.0%) | 6 (100.0%) | 7 (100.0%) | 5 (100.0%) | 18 (100.0%) |
|  | Worsened (MH) | 0 (0.0%) | 0 (0.0%) | 0 (0.0%) | 0 (0.0%) | 0 (0.0%) |
|  | Worsened (AE) | 0 (0.0%) | 0 (0.0%) | 0 (0.0%) | 0 (0.0%) | 0 (0.0%) |
| **Week 96** | N (Missing) | 3 (0) | 7 (0) | 10 (0) | 7 (0) | 24 (0) |
|  | Mean (SD) | 12.40 (1.353) | 14.03 (0.960) | 13.34 (1.297) | 12.60 (1.488) | 13.33 (1.337) |
|  | Normal | 1 (33.3%) | 7 (100.0%) | 9 (90.0%) | 5 (71.4%) | 21 (87.5%) |
|  | NCS | 2 (66.7%) | 0 (0.0%) | 1 (10.0%) | 2 (28.6%) | 3 (12.5%) |
|  | CS (MH) | 0 (0.0%) | 0 (0.0%) | 0 (0.0%) | 0 (0.0%) | 0 (0.0%) |
|  | CS (AE) | 0 (0.0%) | 0 (0.0%) | 0 (0.0%) | 0 (0.0%) | 0 (0.0%) |
| Transition from baseline | Relieved | 0 (0.0%) | 0 (0.0%) | 0 (0.0%) | 0 (0.0%) | 0 (0.0%) |
|  | Unchanged | 3 (100.0%) | 7 (100.0%) | 10 (100.0%) | 7 (100.0%) | 24 (100.0%) |
|  | Worsened (MH) | 0 (0.0%) | 0 (0.0%) | 0 (0.0%) | 0 (0.0%) | 0 (0.0%) |
|  | Worsened (AE) | 0 (0.0%) | 0 (0.0%) | 0 (0.0%) | 0 (0.0%) | 0 (0.0%) |

*NCS: abnormal but not clinically significant, MH: Medical history, AE: Adverse event. Relieved: CS (Medical History or Adverse Event) at baseline to Normal / NCS at visit*

*Unchanged: including Normal at baseline to NCS at visit, NCS at baseline to Normal at visit. Worsened (Medical History): Normal / NCS at baseline to CS (Medical History) at visit. Worsened (Adverse Event): Normal / NCS / CS (Medical History) at baseline to CS (Adverse Event) at visit.*

**Table 8 Summary of Hematocrit [%] - Safety Population**

| **Hematocrit** | | **HA** | **16M** | **32M** | **64M** | **Pooled** |
| --- | --- | --- | --- | --- | --- | --- |
| **Screening** | N (Missing) | 8 (0) | 17 (0) | 17 (0) | 15 (0) | 49 (0) |
|  | Mean (SD) | 36.93 (2.711) | 41.56 (2.257) | 38.99 (3.559) | 39.97 (3.372) | 40.18 (3.229) |
|  | Groups Diff.  P-value (Wilcox_t) | -- | 0.0027***** | 0.1357 | 0.0616 | 0.0103***** |
|  | Normal | 2 (25.0%) | 16 (94.1%) | 13 (76.5%) | 12 (80.0%) | 41 (83.7%) |
|  | NCS | 6 (75.0%) | 1 (5.9%) | 4 (23.5%) | 3 (20.0%) | 8 (16.3%) |
|  | CS (MH) | 0 (0.0%) | 0 (0.0%) | 0 (0.0%) | 0 (0.0%) | 0 (0.0%) |
|  | CS (AE) | 0 (0.0%) | 0 (0.0%) | 0 (0.0%) | 0 (0.0%) | 0 (0.0%) |
| **Week 24** | N (Missing) | 8 (0) | 17 (0) | 17 (0) | 15 (0) | 49 (0) |
|  | Mean (SD) | 37.65 (3.636) | 41.24 (3.162) | 39.55 (3.927) | 40.27 (3.051) | 40.36 (3.420) |
|  | Normal | 2 (25.0%) | 16 (94.1%) | 13 (76.5%) | 13 (86.7%) | 42 (85.7%) |
|  | NCS | 6 (75.0%) | 1 (5.9%) | 4 (23.5%) | 2 (13.3%) | 7 (14.3%) |
|  | CS (MH) | 0 (0.0%) | 0 (0.0%) | 0 (0.0%) | 0 (0.0%) | 0 (0.0%) |
|  | CS (AE) | 0 (0.0%) | 0 (0.0%) | 0 (0.0%) | 0 (0.0%) | 0 (0.0%) |
| Transition from baseline | Relieved | 0 (0.0%) | 0 (0.0%) | 0 (0.0%) | 0 (0.0%) | 0 (0.0%) |
|  | Unchanged | 8 (100.0%) | 17 (100.0%) | 17 (100.0%) | 15 (100.0%) | 49 (100.0%) |
|  | Worsened (MH) | 0 (0.0%) | 0 (0.0%) | 0 (0.0%) | 0 (0.0%) | 0 (0.0%) |
|  | Worsened (AE) | 0 (0.0%) | 0 (0.0%) | 0 (0.0%) | 0 (0.0%) | 0 (0.0%) |
| **Week 36** | N (Missing) | 6 (0) | 12 (0) | 13 (0) | 14 (0) | 39 (0) |
|  | Mean (SD) | 38.22 (3.350) | 41.27 (2.235) | 40.77 (3.198) | 40.01 (3.074) | 40.65 (2.861) |
|  | Normal | 4 (66.7%) | 12 (100%) | 11 (84.6 %) | 11 (78.6 %) | 34 (87.2 %) |
|  | NCS | 2 (33.3%) | 0 (0.0%) | 2 (15.4 %) | 3 (21.4 %) | 5 (12.8 %) |
|  | CS (MH) | 0 (0.0%) | 0 (0.0%) | 0 (0.0%) | 0 (0.0%) | 0 (0.0%) |
|  | CS (AE) | 0 (0.0%) | 0 (0.0%) | 0 (0.0%) | 0 (0.0%) | 0 (0.0%) |
| Transition from baseline | Relieved | 0 (0.0%) | 0 (0.0%) | 0 (0.0%) | 0 (0.0%) | 0 (0.0%) |
|  | Unchanged | 6 (100.0%) | 12 (100.0%) | 13 (100.0%) | 14 (100.0%) | 39 (100.0%) |
|  | Worsened (MH) | 0 (0.0%) | 0 (0.0%) | 0 (0.0%) | 0 (0.0%) | 0 (0.0%) |
|  | Worsened (AE) | 0 (0.0%) | 0 (0.0%) | 0 (0.0%) | 0 (0.0%) | 0 (0.0%) |
| **Week 48** | N (Missing) | 6 (0) | 12 (0) | 13 (0) | 12 (0) | 37 (0) |
|  | Mean (SD) | 38.45 (3.846) | 41.20 (2.436) | 40.64 (2.914) | 40.39 (2.737) | 40.74 (2.655) |
|  | Normal | 2 (33.3%) | 12 (100.0%) | 12 (92.3%) | 11 (91.7%) | 35 (94.6%) |
|  | NCS | 4 (66.7%) | 0 (0.0%) | 1 (7.7%) | 1 (8.3%) | 2 (5.4%) |
|  | CS (MH) | 0 (0.0%) | 0 (0.0%) | 0 (0.0%) | 0 (0.0%) | 0 (0.0%) |
|  | CS (AE) | 0 (0.0%) | 0 (0.0%) | 0 (0.0%) | 0 (0.0%) | 0 (0.0%) |
| Transition from baseline | Relieved | 0 (0.0%) | 0 (0.0%) | 0 (0.0%) | 0 (0.0%) | 0 (0.0%) |
|  | Unchanged | 6 (100.0%) | 12 (100.0%) | 13 (100.0%) | 12 (100.0%) | 37 (100.0%) |
|  | Worsened (MH) | 0 (0.0%) | 0 (0.0%) | 0 (0.0%) | 0 (0.0%) | 0 (0.0%) |
|  | Worsened (AE) | 0 (0.0%) | 0 (0.0%) | 0 (0.0%) | 0 (0.0%) | 0 (0.0%) |
| **Week 72** | N (Missing) | 3 (0) | 6 (0) | 7 (0) | 5 (0) | 18 (0) |
|  | Mean (SD) | 38.47 (2.593) | 41.80 (3.511) | 39.54 (3.976) | 39.18 (3.464) | 40.19 (3.663) |
|  | Normal | 1 (33.3%) | 6 (100.0%) | 6 (85.7%) | 4 (80.0%) | 16 (88.9%) |
|  | NCS | 2 (66.7%) | 0 (0.0%) | 1 (14.3%) | 1 (20.0%) | 2 (11.1%) |
|  | CS (MH) | 0 (0.0%) | 0 (0.0%) | 0 (0.0%) | 0 (0.0%) | 0 (0.0%) |
|  | CS (AE) | 0 (0.0%) | 0 (0.0%) | 0 (0.0%) | 0 (0.0%) | 0 (0.0%) |
| Transition from baseline | Relieved | 0 (0.0%) | 0 (0.0%) | 0 (0.0%) | 0 (0.0%) | 0 (0.0%) |
|  | Unchanged | 3 (100.0%) | 6 (100.0%) | 7 (100.0%) | 5 (100.0%) | 18 (100.0%) |
|  | Worsened (MH) | 0 (0.0%) | 0 (0.0%) | 0 (0.0%) | 0 (0.0%) | 0 (0.0%) |
|  | Worsened (AE) | 0 (0.0%) | 0 (0.0%) | 0 (0.0%) | 0 (0.0%) | 0 (0.0%) |
| **Week 96** | N (Missing) | 3 (0) | 7 (0) | 10 (0) | 7 (0) | 24 (0) |
|  | Mean (SD) | 38.67 (3.623) | 41.90 (2.476) | 40.12 (3.745) | 38.01 (4.093) | 40.03 (3.710) |
|  | Normal | 1 (33.3%) | 7 (100.0%) | 8 (80.0%) | 4 (57.1%) | 19 (79.2%) |
|  | NCS | 2 (66.7%) | 0 (0.0%) | 2 (20.0%) | 3 (42.9%) | 5 (20.8%) |
|  | CS (MH) | 0 (0.0%) | 0 (0.0%) | 0 (0.0%) | 0 (0.0%) | 0 (0.0%) |
|  | CS (AE) | 0 (0.0%) | 0 (0.0%) | 0 (0.0%) | 0 (0.0%) | 0 (0.0%) |
| Transition from baseline | Relieved | 0 (0.0%) | 0 (0.0%) | 0 (0.0%) | 0 (0.0%) | 0 (0.0%) |
|  | Unchanged | 3 (100.0%) | 7 (100.0%) | 10 (100.0%) | 7 (100.0%) | 24 (100.0%) |
|  | Worsened (MH) | 0 (0.0%) | 0 (0.0%) | 0 (0.0%) | 0 (0.0%) | 0 (0.0%) |
|  | Worsened (AE) | 0 (0.0%) | 0 (0.0%) | 0 (0.0%) | 0 (0.0%) | 0 (0.0%) |

*NCS: abnormal but not clinically significant, MH: Medical history, AE: Adverse event. Relieved: CS (Medical History or Adverse Event) at baseline to Normal / NCS at visit*

*Unchanged: including Normal at baseline to NCS at visit, NCS at baseline to Normal at visit. Worsened (Medical History): Normal / NCS at baseline to CS (Medical History) at visit. Worsened (Adverse Event): Normal / NCS / CS (Medical History) at baseline to CS (Adverse Event) at visit.*

**Table 9 Summary of Platelets [10^9^/L] - Safety Population**

| **Platelets** | | **HA** | **16M** | **32M** | **64M** | **Pooled** |
| --- | --- | --- | --- | --- | --- | --- |
| **Screening** | N (Missing) | 8 (0) | 17 (0) | 17 (0) | 15 (0) | 49 (0) |
|  | Mean (SD) | 213.63 (49.897) | 260.12 (74.936) | 273.82 (145.835) | 246.53 (52.923) | 260.71 (99.508) |
|  | Groups Diff.  P-value (Wilcox_t) | -- | 0.1503 | 0.2672 | 0.1791 | 0.1266 |
|  | Normal | 7 (87.5%) | 15 (88.2%) | 14 (82.4%) | 14 (93.3%) | 43 (87.8%) |
|  | NCS | 1 (12.5%) | 2 (11.8%) | 2 (11.8%) | 1 (6.7%) | 5 (10.2%) |
|  | CS (MH) | 0 (0.0%) | 0 (0.0%) | 1 (5.9%) | 0 (0.0%) | 1 (2.0%) |
|  | CS (AE) | 0 (0.0%) | 0 (0.0%) | 0 (0.0%) | 0 (0.0%) | 0 (0.0%) |
| **Week 24** | N (Missing) | 8 (0) | 17 (0) | 17 (0) | 15 (0) | 49 (0) |
|  | Mean (SD) | 202.13 (54.956) | 248.53 (76.291) | 263.12 (126.116) | 244.00 (54.913) | 252.20 (90.495) |
|  | Normal | 7 (87.5%) | 14 (82.4%) | 14 (82.4%) | 14 (93.3%) | 42 (85.7%) |
|  | NCS | 1 (12.5%) | 3 (17.6%) | 2 (11.8%) | 1 (6.7%) | 6 (12.2%) |
|  | CS (MH) | 0 (0.0%) | 0 (0.0%) | 1 (5.9%) | 0 (0.0%) | 1 (2.0%) |
|  | CS (AE) | 0 (0.0%) | 0 (0.0%) | 0 (0.0%) | 0 (0.0%) | 0 (0.0%) |
| Transition from baseline | Relieved | 0 (0.0%) | 0 (0.0%) | 0 (0.0%) | 0 (0.0%) | 0 (0.0%) |
|  | Unchanged | 8 (100.0%) | 17 (100.0%) | 17 (100.0%) | 15 (100.0%) | 49 (100.0%) |
|  | Worsened (MH) | 0 (0.0%) | 0 (0.0%) | 0 (0.0%) | 0 (0.0%) | 0 (0.0%) |
|  | Worsened (AE) | 0 (0.0%) | 0 (0.0%) | 0 (0.0%) | 0 (0.0%) | 0 (0.0%) |
| **Week 36** | N (Missing) | 6 (0) | 12 (0) | 13 (0) | 14 (0) | 39 (0) |
|  | Mean (SD) | 210.83 (57.087) | 239.58 (66.894) | 290.38 (155.269) | 235.07 (37.333) | 254.90 (100.175) |
|  | Normal | 6 (100.0 %) | 11 (91.7 %) | 11 (84.6 %) | 14 (100.0 %) | 36 (92.3%) |
|  | NCS | 0 (0.0% | 1 (8.3 %) | 1 (7.7 %) | 0 (0.0%) | 2 (5.1%) |
|  | CS (MH) | 0 (0.0%) | 0 (0.0%) | 1 (7.7 %) | 0 (0.0%) | 1 (2.6%) |
|  | CS (AE) | 0 (0.0%) | 0 (0.0%) | 0 (0.0%) | 0 (0.0%) | 0 (0.0%) |
| Transition from baseline | Relieved | 0 (0.0%) | 0 (0.0%) | 0 (0.0%) | 0 (0.0%) | 0 (0.0%) |
|  | Unchanged | 6 (100.0%) | 12 (100.0%) | 13 (100.0%) | 14 (100.0%) | 39 (100.0%) |
|  | Worsened (MH) | 0 (0.0%) | 0 (0.0%) | 0 (0.0%) | 0 (0.0%) | 0 (0.0%) |
|  | Worsened (AE) | 0 (0.0%) | 0 (0.0%) | 0 (0.0%) | 0 (0.0%) | 0 (0.0%) |
| **Week 48** | N (Missing) | 6 (0) | 12 (0) | 13 (0) | 12 (0) | 37 (0) |
|  | Mean (SD) | 223.33 (83.092) | 238.92 (63.559) | 330.38 (248.257) | 241.58 (46.738) | 271.92 (156.044) |
|  | Normal | 4 (66.7%) | 11 (91.7%) | 11 (84.6%) | 11 (91.7%) | 33 (89.2%) |
|  | NCS | 2 (33/3%) | 1 (8.3%) | 1 (7.7%) | 1 (8.3%) | 3 (8.1%) |
|  | CS (MH) | 0 (0.0%) | 0 (0.0%) | 1 (7.7%) | 0 (0.0%) | 1 (2.7%) |
|  | CS (AE) | 0 (0.0%) | 0 (0.0%) | 0 (0.0%) | 0 (0.0%) | 0 (0.0%) |
| Transition from baseline | Relieved | 0 (0.0%) | 0 (0.0%) | 0 (0.0%) | 0 (0.0%) | 0 (0.0%) |
|  | Unchanged | 6 (100.0%) | 12 (100.0%) | 13 (100.0%) | 12 (100.0%) | 37 (100.0%) |
|  | Worsened (MH) | 0 (0.0%) | 0 (0.0%) | 0 (0.0%) | 0 (0.0%) | 0 (0.0%) |
|  | Worsened (AE) | 0 (0.0%) | 0 (0.0%) | 0 (0.0%) | 0 (0.0%) | 0 (0.0%) |
| **Week 72** | N (Missing) | 3 (0) | 6 (0) | 7 (0) | 5 (0) | 18 (0) |
|  | Mean (SD) | 211.00 (55.073) | 222.67 (82.788) | 263.00 (136.060) | 239.40 (36.046) | 243.00 (95.759) |
|  | Normal | 3 (100.0%) | 5 (83.3%) | 6 (85.7%) | 5 (100.0%) | 16 (88.9%) |
|  | NCS | 0 (0.0%) | 1 (16.7%) | 0 (0.0%) | 0 (0.0%) | 1 (5.6%) |
|  | CS (MH) | 0 (0.0%) | 0 (0.0%) | 1 (14.3%) | 0 (0.0%) | 1 (5.6%) |
|  | CS (AE) | 0 (0.0%) | 0 (0.0%) | 0 (0.0%) | 0 (0.0%) | 0 (0.0%) |
| Transition from baseline | Relieved | 0 (0.0%) | 0 (0.0%) | 0 (0.0%) | 0 (0.0%) | 0 (0.0%) |
|  | Unchanged | 3 (100.0%) | 6 (100.0%) | 7 (100.0%) | 5 (100.0%) | 18 (100.0%) |
|  | Worsened (MH) | 0 (0.0%) | 0 (0.0%) | 0 (0.0%) | 0 (0.0%) | 0 (0.0%) |
|  | Worsened (AE) | 0 (0.0%) | 0 (0.0%) | 0 (0.0%) | 0 (0.0%) | 0 (0.0%) |
| **Week 96** | N (Missing) | 3 (0) | 7 (0) | 10 (0) | 7 (0) | 24 (0) |
|  | Mean (SD) | 192.00 (44.238) | 220.71 (58.128) | 319.80 (215.420) | 243.71 (30.543) | 268.71 (145.978) |
|  | Normal | 3 (100.0%) | 6 (85.7%) | 9 (90.0%) | 7 (100.0%) | 22 (91.7%) |
|  | NCS | 0 (0.0%) | 1 (14.3%) | 0 (0.0%) | 0 (0.0%) | 1 (4.2%) |
|  | CS (MH) | 0 (0.0%) | 0 (0.0%) | 1 (10.0%) | 0 (0.0%) | 1 (4.2%) |
|  | CS (AE) | 0 (0.0%) | 0 (0.0%) | 0 (0.0%) | 0 (0.0%) | 0 (0.0%) |
| Transition from baseline | Relieved | 0 (0.0%) | 0 (0.0%) | 0 (0.0%) | 0 (0.0%) | 0 (0.0%) |
|  | Unchanged | 3 (100.0%) | 7 (100.0%) | 10 (100.0%) | 7 (100.0%) | 24 (100.0%) |
|  | Worsened (MH) | 0 (0.0%) | 0 (0.0%) | 0 (0.0%) | 0 (0.0%) | 0 (0.0%) |
|  | Worsened (AE) | 0 (0.0%) | 0 (0.0%) | 0 (0.0%) | 0 (0.0%) | 0 (0.0%) |

*NCS: abnormal but not clinically significant, MH: Medical history, AE: Adverse event. Relieved: CS (Medical History or Adverse Event) at baseline to Normal / NCS at visit*

*Unchanged: including Normal at baseline to NCS at visit, NCS at baseline to Normal at visit. Worsened (Medical History): Normal / NCS at baseline to CS (Medical History) at visit. Worsened (Adverse Event): Normal / NCS / CS (Medical History) at baseline to CS (Adverse Event) at visit.*

**Table 10 Summary of Red Blood Cells [10^12^/L] - Safety Population**

| **Red Blood Cells** | | **HA** | **16M** | **32M** | **64M** | **Pooled** |
| --- | --- | --- | --- | --- | --- | --- |
| **Screening** | N (Missing) | 8 (0) | 17 (0) | 17 (0) | 15 (0) | 49 (0) |
|  | Mean (SD) | 4.36 (0.850) | 4.67 (0.288) | 4.26 (0.575) | 4.60 (0.401) | 4.51 (0.469) |
|  | Groups Diff.  P-value (Wilcox_t) | -- | 0.0788 | 0.8627 | 0.1434 | 0.1806 |
|  | Normal | 2 (25.0%) | 15 (88.2%) | 10 (58.8%) | 14 (93.3%) | 39 (79.6%) |
|  | NCS | 6 (75.0%) | 2 (11.8%) | 7 (41.2%) | 1 (6.7%) | 10 (20.4%) |
|  | CS (MH) | 0 (0.0%) | 0 (0.0%) | 0 (0.0%) | 0 (0.0%) | 0 (0.0%) |
|  | CS (AE) | 0 (0.0%) | 0 (0.0%) | 0 (0.0%) | 0 (0.0%) | 0 (0.0%) |
| **Week 24** | N (Missing) | 8 (0) | 17 (0) | 17 (0) | 15 (0) | 49 (0) |
|  | Mean (SD) | 4.40 (0.753) | 4.62 (0.371) | 4.32 (0.582) | 4.58 (0.423) | 4.51 (0.479) |
|  | Normal | 3 (37.5%) | 14 (82.4%) | 10 (58.8%) | 12 (80.0%) | 36 (73.5%) |
|  | NCS | 5 (62.5%) | 3 (17.6%) | 7 (41.2%) | 3 (20.0%) | 13 (26.5%) |
|  | CS (MH) | 0 (0.0%) | 0 (0.0%) | 0 (0.0%) | 0 (0.0%) | 0 (0.0%) |
|  | CS (AE) | 0 (0.0%) | 0 (0.0%) | 0 (0.0%) | 0 (0.0%) | 0 (0.0%) |
| Transition from baseline | Relieved | 0 (0.0%) | 0 (0.0%) | 0 (0.0%) | 0 (0.0%) | 0 (0.0%) |
|  | Unchanged | 8 (100.0%) | 17 (100.0%) | 17 (100.0%) | 15 (100.0%) | 49 (100.0%) |
|  | Worsened (MH) | 0 (0.0%) | 0 (0.0%) | 0 (0.0%) | 0 (0.0%) | 0 (0.0%) |
|  | Worsened (AE) | 0 (0.0%) | 0 (0.0%) | 0 (0.0%) | 0 (0.0%) | 0 (0.0%) |
| **Week 36** | N (Missing) | 6 (0) | 12 (0) | 13 (0) | 14 (0) | 39 (0) |
|  | Mean (SD) | 4.57 (0.895) | 4.65 (0.390) | 4.46 (0.514) | 4.50 (0.421) | 4.53 (0.441) |
|  | Normal | 4 (66.7%) | 11 (91.7%) | 9 (69.2%) | 11 (78.6%) | 31 (79.5%) |
|  | NCS | 2 (33.3%) | 1 (8.3%) | 4 (30.8%) | 3 (21.4%) | 8 (20.5%) |
|  | CS (MH) | 0 (0.0%) | 0 (0.0%) | 0 (0.0%) | 0 (0.0%) | 0 (0.0%) |
|  | CS (AE) | 0 (0.0%) | 0 (0.0%) | 0 (0.0%) | 0 (0.0%) | 0 (0.0%) |
| Transition from baseline | Relieved | 0 (0.0%) | 0 (0.0%) | 0 (0.0%) | 0 (0.0%) | 0 (0.0%) |
|  | Unchanged | 6 (100.0%) | 12 (100.0%) | 13 (100.0%) | 14 (100.0%) | 39 (100.0%) |
|  | Worsened (MH) | 0 (0.0%) | 0 (0.0%) | 0 (0.0%) | 0 (0.0%) | 0 (0.0%) |
|  | Worsened (AE) | 0 (0.0%) | 0 (0.0%) | 0 (0.0%) | 0 (0.0%) | 0 (0.0%) |
| **Week 48** | N (Missing) | 6 (0) | 12 (0) | 13 (0) | 12 (0) | 37 (0) |
|  | Mean (SD) | 4.56 (0.931) | 4.66 (0.441) | 4.45 (0.478) | 4.53 (0.423) | 4.55 (0.445) |
|  | Normal | 3 (50.0%) | 11 (91.7%) | 9 (69.2%) | 9 (75.0%) | 29 (78.4%) |
|  | NCS | 3 (50.0%) | 1 (8.3%) | 4 (30.8%) | 3 (25.0%) | 8 (21.6%) |
|  | CS (MH) | 0 (0.0%) | 0 (0.0%) | 0 (0.0%) | 0 (0.0%) | 0 (0.0%) |
|  | CS (AE) | 0 (0.0%) | 0 (0.0%) | 0 (0.0%) | 0 (0.0%) | 0 (0.0%) |
| Transition from baseline | Relieved | 0 (0.0%) | 0 (0.0%) | 0 (0.0%) | 0 (0.0%) | 0 (0.0%) |
|  | Unchanged | 6 (100.0%) | 12 (100.0%) | 13 (100.0%) | 12 (100.0%) | 37 (100.0%) |
|  | Worsened (MH) | 0 (0.0%) | 0 (0.0%) | 0 (0.0%) | 0 (0.0%) | 0 (0.0%) |
|  | Worsened (AE) | 0 (0.0%) | 0 (0.0%) | 0 (0.0%) | 0 (0.0%) | 0 (0.0%) |
| **Week 72** | N (Missing) | 3 (0) | 6 (0) | 7 (0) | 5 (0) | 18 (0) |
|  | Mean (SD) | 5.08 (0.906) | 4.58 (0.382) | 4.22 (0.554) | 4.27 (0.484) | 4.35 (0.482) |
|  | Normal | 2 (66.7%) | 5 (83.3%) | 3 (42.9%) | 3 (60.0%) | 11 (61.1%) |
|  | NCS | 1 (33.3%) | 1 (16.7%) | 4 (57.1%) | 2 (40.0%) | 7 (38.9%) |
|  | CS (MH) | 0 (0.0%) | 0 (0.0%) | 0 (0.0%) | 0 (0.0%) | 0 (0.0%) |
|  | CS (AE) | 0 (0.0%) | 0 (0.0%) | 0 (0.0%) | 0 (0.0%) | 0 (0.0%) |
| Transition from baseline | Relieved | 0 (0.0%) | 0 (0.0%) | 0 (0.0%) | 0 (0.0%) | 0 (0.0%) |
|  | Unchanged | 3 (100.0%) | 6 (100.0%) | 7 (100.0%) | 5 (100.0%) | 18 (100.0%) |
|  | Worsened (MH) | 0 (0.0%) | 0 (0.0%) | 0 (0.0%) | 0 (0.0%) | 0 (0.0%) |
|  | Worsened (AE) | 0 (0.0%) | 0 (0.0%) | 0 (0.0%) | 0 (0.0%) | 0 (0.0%) |
| **Week 96** | N (Missing) | 3 (0) | 7 (0) | 10 (0) | 7 (0) | 24 (0) |
|  | Mean (SD) | 5.07 (0.791) | 4.63 (0.322) | 4.35 (0.545) | 4.32 (0.532) | 4.42 (0.485) |
|  | Normal | 2 (66.7%) | 7 (100.0%) | 7 (70.0%) | 4 (57.1%) | 18 (75.0%) |
|  | NCS | 1 (33.3%) | 0 (0.0%) | 3 (30.0%) | 3 (42.9%) | 6 (25.0%) |
|  | CS (MH) | 0 (0.0%) | 0 (0.0%) | 0 (0.0%) | 0 (0.0%) | 0 (0.0%) |
|  | CS (AE) | 0 (0.0%) | 0 (0.0%) | 0 (0.0%) | 0 (0.0%) | 0 (0.0%) |
| Transition from baseline | Relieved | 0 (0.0%) | 0 (0.0%) | 0 (0.0%) | 0 (0.0%) | 0 (0.0%) |
|  | Unchanged | 3 (100.0%) | 7 (100.0%) | 10 (100.0%) | 7 (100.0%) | 24 (100.0%) |
|  | Worsened (MH) | 0 (0.0%) | 0 (0.0%) | 0 (0.0%) | 0 (0.0%) | 0 (0.0%) |
|  | Worsened (AE) | 0 (0.0%) | 0 (0.0%) | 0 (0.0%) | 0 (0.0%) | 0 (0.0%) |

*NCS: abnormal but not clinically significant, MH: Medical history, AE: Adverse event. Relieved: CS (Medical History or Adverse Event) at baseline to Normal / NCS at visit*

*Unchanged: including Normal at baseline to NCS at visit, NCS at baseline to Normal at visit. Worsened (Medical History): Normal / NCS at baseline to CS (Medical History) at visit. Worsened (Adverse Event): Normal / NCS / CS (Medical History) at baseline to CS (Adverse Event) at visit.*

**Table 11 Summary of Aspartate Aminotransferase [U/L] - Safety Population**

| **Aspartate Aminotransferase** | | **HA** | **16M** | **32M** | **64M** | **Pooled** |
| --- | --- | --- | --- | --- | --- | --- |
| **Screening** | N (Missing) | 8 (0) | 17 (0) | 17 (0) | 15 (0) | 49 (0) |
|  | Mean (SD) | 22.13 (6.446) | 25.71 (7.928) | 34.18 (20.169) | 29.07 (8.293) | 29.67 (13.765) |
|  | Groups Diff.  P-value (Wilcox_t) | -- | 0.1415 | 0.0464***** | 0.0574 | 0.0348***** |
|  | Normal | 8 (100.0%) | 15 (88.2%) | 13 (76.5%) | 12 (80.0%) | 40 (81.6%) |
|  | NCS | 0 (0.0%) | 2 (11.8%) | 4 (23.5%) | 3 (20.0%) | 9 (18.4%) |
|  | CS (MH) | 0 (0.0%) | 0 (0.0%) | 0 (0.0%) | 0 (0.0%) | 0 (0.0%) |
|  | CS (AE) | 0 (0.0%) | 0 (0.0%) | 0 (0.0%) | 0 (0.0%) | 0 (0.0%) |
| **Week 24** | N (Missing) | 8 (0) | 17 (0) | 17 (0) | 15 (0) | 49 (0) |
|  | Mean (SD) | 23.13 (4.704) | 26.24 (8.828) | 35.76 (24.173) | 29.73 (9.625) | 30.61 (16.254) |
|  | Normal | 8 (100.0%) | 15 (88.2%) | 12 (70.6%) | 12 (80.0%) | 39 (79.6%) |
|  | NCS | 0 (0.0%) | 2 (11.8%) | 5 (29.4%) | 3 (20.0%) | 10 (20.4%) |
|  | CS (MH) | 0 (0.0%) | 0 (0.0%) | 0 (0.0%) | 0 (0.0%) | 0 (0.0%) |
|  | CS (AE) | 0 (0.0%) | 0 (0.0%) | 0 (0.0%) | 0 (0.0%) | 0 (0.0%) |
| Transition from baseline | Relieved | 0 (0.0%) | 0 (0.0%) | 0 (0.0%) | 0 (0.0%) | 0 (0.0%) |
|  | Unchanged | 8 (100.0%) | 17 (100.0%) | 17 (100.0%) | 15 (100.0%) | 49 (100.0%) |
|  | Worsened (MH) | 0 (0.0%) | 0 (0.0%) | 0 (0.0%) | 0 (0.0%) | 0 (0.0%) |
|  | Worsened (AE) | 0 (0.0%) | 0 (0.0%) | 0 (0.0%) | 0 (0.0%) | 0 (0.0%) |
| **Week 36** | N (Missing) | 6 (0) | 12 (0) | 13 (0) | 12 (2) | 37 (2) |
|  | Mean (SD) | 23.83 (7.985) | 26.33 (4.250) | 36.54 (23.132) | 29.92 (9.278) | 31.08 (15.130) |
|  | Normal | 6 (100.0%) | 11 (91.7%) | 9 (69.2%) | 8 (66.7%) | 28 (75.7%) |
|  | NCS | 0 (0.0%) | 1 (8.3%) | 4 (30.8%) | 4 (33.3%) | 9 (24.3%) |
|  | CS (MH) | 0 (0.0%) | 0 (0.0%) | 0 (0.0%) | 0 (0.0%) | 0 (0.0%) |
|  | CS (AE) | 0 (0.0%) | 0 (0.0%) | 0 (0.0%) | 0 (0.0%) | 0 (0.0%) |
| Transition from baseline | Relieved | 0 (0.0%) | 0 (0.0%) | 0 (0.0%) | 0 (0.0%) | 0 (0.0%) |
|  | Unchanged | 6 (100.0%) | 12 (100.0%) | 13 (100.0%) | 12 (100.0%) | 37 (100.0%) |
|  | Worsened (MH) | 0 (0.0%) | 0 (0.0%) | 0 (0.0%) | 0 (0.0%) | 0 (0.0%) |
|  | Worsened (AE) | 0 (0.0%) | 0 (0.0%) | 0 (0.0%) | 0 (0.0%) | 0 (0.0%) |
| **Week 48** | N (Missing) | 6 (0) | 12 (0) | 13 (0) | 12 (0) | 37 (0) |
|  | Mean (SD) | 26.67 (15.552) | 24.33 (5.836) | 32.00 (10.416) | 30.92 (13.372) | 29.16 (10.626) |
|  | Normal | 5 (83.3%) | 12 (100.0%) | 9 (69.2%) | 8 (66.7%) | 29 (78.4%) |
|  | NCS | 1 (16.7%) | 0 (0.0%) | 3 (23.1%) | 3 (25.0%) | 6 (16.2%) |
|  | CS (MH) | 0 (0.0%) | 0 (0.0%) | 1 (7.7%) | 0 (0.0%) | 1 (2.7%) |
|  | CS (AE) | 0 (0.0%) | 0 (0.0%) | 0 (0.0%) | 1 (8.3%) | 1 (2.7%) |
| Transition from baseline | Relieved | 0 (0.0%) | 0 (0.0%) | 0 (0.0%) | 0 (0.0%) | 0 (0.0%) |
|  | Unchanged | 6 (100.0%) | 12 (100.0%) | 12 (92.3%) | 11 (91.7%) | 35 (94.6%) |
|  | Worsened (MH) | 0 (0.0%) | 0 (0.0%) | 1 (7.7%) | 0 (0.0%) | 1 (2.7%) |
|  | Worsened (AE) | 0 (0.0%) | 0 (0.0%) | 0 (0.0%) | 1 (8.3%) | 1 (2.7%) |
| **Week 72** | N (Missing) | 3 (0) | 6 (0) | 7 (0) | 5 (0) | 18 (0) |
|  | Mean (SD) | 21.00 (1.000) | 29.00 (6.132) | 26.29 (6.775) | 27.00 (10.025) | 27.39 (7.237) |
|  | Normal | 3 (100.0%) | 6 (100.0%) | 6 (85.7%) | 4 (80.0%) | 16 (88.9%) |
|  | NCS | 0 (0.0%) | 0 (0.0%) | 1 (14.3%) | 1 (20.0%) | 2 (11.1%) |
|  | CS (MH) | 0 (0.0%) | 0 (0.0%) | 0 (0.0%) | 0 (0.0%) | 0 (0.0%) |
|  | CS (AE) | 0 (0.0%) | 0 (0.0%) | 0 (0.0%) | 0 (0.0%) | 0 (0.0%) |
| Transition from baseline | Relieved | 0 (0.0%) | 0 (0.0%) | 0 (0.0%) | 0 (0.0%) | 0 (0.0%) |
|  | Unchanged | 3 (100.0%) | 6 (100.0%) | 7 (100.0%) | 5 (100.0%) | 18 (100.0%) |
|  | Worsened (MH) | 0 (0.0%) | 0 (0.0%) | 0 (0.0%) | 0 (0.0%) | 0 (0.0%) |
|  | Worsened (AE) | 0 (0.0%) | 0 (0.0%) | 0 (0.0%) | 0 (0.0%) | 0 (0.0%) |
| **Week 96** | N (Missing) | 3 (0) | 7 (0) | 10 (0) | 7 (0) | 24 (0) |
|  | Mean (SD) | 18.67 (3.215) | 26.86 (7.010) | 29.10 (7.666) | 27.00 (7.767) | 27.83 (7.263) |
|  | Normal | 3 (100.0%) | 7 (100.0%) | 8 (80.0%) | 7 (100.0%) | 22 (91.7%) |
|  | NCS | 0 (0.0%) | 0 (0.0%) | 2 (20.0%) | 0 (0.0%) | 2 (8.3%) |
|  | CS (MH) | 0 (0.0%) | 0 (0.0%) | 0 (0.0%) | 0 (0.0%) | 0 (0.0%) |
|  | CS (AE) | 0 (0.0%) | 0 (0.0%) | 0 (0.0%) | 0 (0.0%) | 0 (0.0%) |
| Transition from baseline | Relieved | 0 (0.0%) | 0 (0.0%) | 0 (0.0%) | 0 (0.0%) | 0 (0.0%) |
|  | Unchanged | 3 (100.0%) | 7 (100.0%) | 10 (100.0%) | 7 (100.0%) | 24 (100.0%) |
|  | Worsened (MH) | 0 (0.0%) | 0 (0.0%) | 0 (0.0%) | 0 (0.0%) | 0 (0.0%) |
|  | Worsened (AE) | 0 (0.0%) | 0 (0.0%) | 0 (0.0%) | 0 (0.0%) | 0 (0.0%) |

*NCS: abnormal but not clinically significant, MH: Medical history, AE: Adverse event. Relieved: CS (Medical History or Adverse Event) at baseline to Normal / NCS at visit*

*Unchanged: including Normal at baseline to NCS at visit, NCS at baseline to Normal at visit. Worsened (Medical History): Normal / NCS at baseline to CS (Medical History) at visit. Worsened (Adverse Event): Normal / NCS / CS (Medical History) at baseline to CS (Adverse Event) at visit.*

**Table 12 Summary of Alanine Aminotransferase [U/L] - Safety Population**

| **Alanine Aminotransferase** | | **HA** | **16M** | **32M** | **64M** | **Pooled** |
| --- | --- | --- | --- | --- | --- | --- |
| **Screening** | N (Missing) | 8 (0) | 17 (0) | 17 (0) | 15 (0) | 49 (0) |
|  | Mean (SD) | 16.63 (4.138) | 24.00 (10.192) | 33.47 (27.398) | 25.93 (12.418) | 27.88 (18.639) |
|  | Groups Diff.  P-value (Wilcox_t) | -- | 0.0402***** | 0.0463***** | 0.0695 | 0.0206***** |
|  | Normal | 8 (100.0%) | 15 (88.2%) | 12 (70.6%) | 12 (80.0%) | 39 (79.6%) |
|  | NCS | 0 (0.0%) | 2 (11.8%) | 5 (29.4%) | 3 (20.0%) | 10 (20.4%) |
|  | CS (MH) | 0 (0.0%) | 0 (0.0%) | 0 (0.0%) | 0 (0.0%) | 0 (0.0%) |
|  | CS (AE) | 0 (0.0%) | 0 (0.0%) | 0 (0.0%) | 0 (0.0%) | 0 (0.0%) |
| **Week 24** | N (Missing) | 8 (0) | 17 (0) | 17 (0) | 15 (0) | 49 (0) |
|  | Mean (SD) | 17.25 (5.651) | 24.59 (12.435) | 33.82 (21.302) | 28.47 (16.331) | 28.98 (17.199) |
|  | Normal | 8 (100.0%) | 15 (88.2%) | 12 (70.6%) | 12 (80.0%) | 39 (79.6%) |
|  | NCS | 0 (0.0%) | 2 (11.8%) | 5 (29.4%) | 3 (20.0%) | 10 (20.4%) |
|  | CS (MH) | 0 (0.0%) | 0 (0.0%) | 0 (0.0%) | 0 (0.0%) | 0 (0.0%) |
|  | CS (AE) | 0 (0.0%) | 0 (0.0%) | 0 (0.0%) | 0 (0.0%) | 0 (0.0%) |
| Transition from baseline | Relieved | 0 (0.0%) | 0 (0.0%) | 0 (0.0%) | 0 (0.0%) | 0 (0.0%) |
|  | Unchanged | 8 (100.0%) | 17 (100.0%) | 17 (100.0%) | 15 (100.0%) | 49 (100.0%) |
|  | Worsened (MH) | 0 (0.0%) | 0 (0.0%) | 0 (0.0%) | 0 (0.0%) | 0 (0.0%) |
|  | Worsened (AE) | 0 (0.0%) | 0 (0.0%) | 0 (0.0%) | 0 (0.0%) | 0 (0.0%) |
| **Week 36** | N (Missing) | 6 (0) | 12 (0) | 13 (0) | 13 (1) | 38 (1) |
|  | Mean (SD) | 17.17 (7.223) | 24.33 (9.149) | 29.00 (19.222) | 25.23 (9.347) | 26.24 (13.314) |
|  | Normal | 6 (100.0%) | 11 (91.7%) | 11 (84.6%) | 12 (92.3%) | 34 (89.5%) |
|  | NCS | 0 (0.0%) | 1 (8.3%) | 2 (15.4%) | 1 (7.7%) | 4 (10.5%) |
|  | CS (MH) | 0 (0.0%) | 0 (0.0%) | 0 (0.0%) | 0 (0.0%) | 0 (0.0%) |
|  | CS (AE) | 0 (0.0%) | 0 (0.0%) | 0 (0.0%) | 0 (0.0%) | 0 (0.0%) |
| Transition from baseline | Relieved | 0 (0.0%) | 0 (0.0%) | 0 (0.0%) | 0 (0.0%) | 0 (0.0%) |
|  | Unchanged | 6 (100.0%) | 12 (100.0%) | 13 (100.0%) | 13 (100.0%) | 38 (100.0%) |
|  | Worsened (MH) | 0 (0.0%) | 0 (0.0%) | 0 (0.0%) | 0 (0.0%) | 0 (0.0%) |
|  | Worsened (AE) | 0 (0.0%) | 0 (0.0%) | 0 (0.0%) | 0 (0.0%) | 0 (0.0%) |
| **Week 48** | N (Missing) | 6 (0) | 12 (0) | 13 (0) | 12 (0) | 37 (0) |
|  | Mean (SD) | 21.83 (16.762) | 22.17 (11.938) | 33.92 (26.292) | 29.50 (21.437) | 28.68 (20.944) |
|  | Normal | 5 (83.3%) | 11 (91.7%) | 9 (69.2%) | 10 (83.3%) | 30 (81.1%) |
|  | NCS | 1 (16.7%) | 1 (8.3%) | 3 (23.1%) | 1 (8.3%) | 5 (13.5%) |
|  | CS (MH) | 0 (0.0%) | 0 (0.0%) | 1 (7.7%) | 0 (0.0%) | 1 (2.7%) |
|  | CS (AE) | 0 (0.0%) | 0 (0.0%) | 0 (0.0%) | 1 (8.3%) | 1 (2.7%) |
| Transition from baseline | Relieved | 0 (0.0%) | 0 (0.0%) | 0 (0.0%) | 0 (0.0%) | 0 (0.0%) |
|  | Unchanged | 6 (100.0%) | 12 (100.0%) | 12 (92.3%) | 11 (91.7%) | 35 (94.6%) |
|  | Worsened (MH) | 0 (0.0%) | 0 (0.0%) | 1 (7.7%) | 0 (0.0%) | 1 (2.7%) |
|  | Worsened (AE) | 0 (0.0%) | 0 (0.0%) | 0 (0.0%) | 1 (8.3%) | 1 (2.7%) |
| **Week 72** | N (Missing) | 3 (0) | 6 (0) | 7 (0) | 5 (0) | 18 (0) |
|  | Mean (SD) | 12.00 (6.000) | 27.83 (16.952) | 21.00 (12.410) | 21.80 (10.756) | 23.50 (13.272) |
|  | Normal | 3 (100.0%) | 4 (66.7%) | 6 (85.7%) | 5 (100.0%) | 15 (83.3%) |
|  | NCS | 0 (0.0%) | 2 (33.3%) | 1 (14.3%) | 0 (0.0%) | 3 (16.7%) |
|  | CS (MH) | 0 (0.0%) | 0 (0.0%) | 0 (0.0%) | 0 (0.0%) | 0 (0.0%) |
|  | CS (AE) | 0 (0.0%) | 0 (0.0%) | 0 (0.0%) | 0 (0.0%) | 0 (0.0%) |
| Transition from baseline | Relieved | 0 (0.0%) | 0 (0.0%) | 0 (0.0%) | 0 (0.0%) | 0 (0.0%) |
|  | Unchanged | 3 (100.0%) | 6 (100.0%) | 7 (100.0%) | 5 (100.0%) | 18 (100.0%) |
|  | Worsened (MH) | 0 (0.0%) | 0 (0.0%) | 0 (0.0%) | 0 (0.0%) | 0 (0.0%) |
|  | Worsened (AE) | 0 (0.0%) | 0 (0.0%) | 0 (0.0%) | 0 (0.0%) | 0 (0.0%) |
| **Week 96** | N (Missing) | 3 (0) | 7 (0) | 10 (0) | 7 (0) | 24 (0) |
|  | Mean (SD) | 14.67 (5.774) | 23.43 (12.205) | 25.50 (9.419) | 23.00 (9.504) | 24.17 (9.924) |
|  | Normal | 3 (100.0%) | 6 (85.7%) | 8 (80.0%) | 7 (100.0%) | 21 (87.5%) |
|  | NCS | 0 (0.0%) | 1 (14.3%) | 2 (20.0%) | 0 (0.0%) | 3 (12.5%) |
|  | CS (MH) | 0 (0.0%) | 0 (0.0%) | 0 (0.0%) | 0 (0.0%) | 0 (0.0%) |
|  | CS (AE) | 0 (0.0%) | 0 (0.0%) | 0 (0.0%) | 0 (0.0%) | 0 (0.0%) |
| Transition from baseline | Relieved | 0 (0.0%) | 0 (0.0%) | 0 (0.0%) | 0 (0.0%) | 0 (0.0%) |
|  | Unchanged | 3 (100.0%) | 7 (100.0%) | 10 (100.0%) | 7 (100.0%) | 24 (100.0%) |
|  | Worsened (MH) | 0 (0.0%) | 0 (0.0%) | 0 (0.0%) | 0 (0.0%) | 0 (0.0%) |
|  | Worsened (AE) | 0 (0.0%) | 0 (0.0%) | 0 (0.0%) | 0 (0.0%) | 0 (0.0%) |

*NCS: abnormal but not clinically significant, MH: Medical history, AE: Adverse event. Relieved: CS (Medical History or Adverse Event) at baseline to Normal / NCS at visit*

*Unchanged: including Normal at baseline to NCS at visit, NCS at baseline to Normal at visit. Worsened (Medical History): Normal / NCS at baseline to CS (Medical History) at visit. Worsened (Adverse Event): Normal / NCS / CS (Medical History) at baseline to CS (Adverse Event) at visit.*

**Table 13 Summary of Creatinine [μmol/L] - Safety Population**

| **Creatinine** | | **HA** | **16M** | **32M** | **64M** | **Pooled** |
| --- | --- | --- | --- | --- | --- | --- |
| **Screening** | N (Missing) | 8 (0) | 17 (0) | 17 (0) | 15 (0) | 49 (0) |
|  | Mean (SD) | 78.46 (9.111) | 65.63 (14.294) | 75.30 (27.409) | 66.12 (11.950) | 69.13 (19.514) |
|  | Groups Diff.  P-value (Wilcox_t) | -- | 0.0139***** | 0.1925 | 0.0365***** | 0.0202***** |
|  | Normal | 8 (100.0%) | 17 (100.0%) | 16 (94.1%) | 15 (100.0%) | 48 (98.0%) |
|  | NCS | 0 (0.0%) | 0 (0.0%) | 1 (5.9%) | 0 (0.0%) | 1 (2.0%) |
|  | CS (MH) | 0 (0.0%) | 0 (0.0%) | 0 (0.0%) | 0 (0.0%) | 0 (0.0%) |
|  | CS (AE) | 0 (0.0%) | 0 (0.0%) | 0 (0.0%) | 0 (0.0%) | 0 (0.0%) |
| **Week 24** | N (Missing) | 8 (0) | 17 (0) | 17 (0) | 15 (0) | 49 (0) |
|  | Mean (SD) | 73.59 (10.425) | 65.31 (16.387) | 77.79 (34.898) | 66.30 (13.740) | 69.95 (24.168) |
|  | Normal | 8 (100.0%) | 17 (100.0%) | 16 (94.1%) | 15 (100.0%) | 48 (98.0%) |
|  | NCS | 0 (0.0%) | 0 (0.0%) | 1 (5.9%) | 0 (0.0%) | 1 (2.0%) |
|  | CS (MH) | 0 (0.0%) | 0 (0.0%) | 0 (0.0%) | 0 (0.0%) | 0 (0.0%) |
|  | CS (AE) | 0 (0.0%) | 0 (0.0%) | 0 (0.0%) | 0 (0.0%) | 0 (0.0%) |
| Transition from baseline | Relieved | 0 (0.0%) | 0 (0.0%) | 0 (0.0%) | 0 (0.0%) | 0 (0.0%) |
|  | Unchanged | 8 (100.0%) | 17 (100.0%) | 17 (100.0%) | 15 (100.0%) | 49 (100.0%) |
|  | Worsened (MH) | 0 (0.0%) | 0 (0.0%) | 0 (0.0%) | 0 (0.0%) | 0 (0.0%) |
|  | Worsened (AE) | 0 (0.0%) | 0 (0.0%) | 0 (0.0%) | 0 (0.0%) | 0 (0.0%) |
| **Week 36** | N (Missing) | 6 (0) | 12 (0) | 13 (0) | 12 (2) | 37 (2) |
|  | Mean (SD) | 73.67 (9.775) | 62.54 (15.724) | 69.09 (20.621) | 66.30 (15.162) | 66.06 (17.175) |
|  | Normal | 6 (100.0%) | 12 (100.0%) | 12 (92.3%) | 11 (91.7%) | 35 (94.6%) |
|  | NCS | 0 (0.0%) | 0 (0.0%) | 1 (7.7%) | 1 (8.3%) | 2 (5.4%) |
|  | CS (MH) | 0 (0.0%) | 0 (0.0%) | 0 (0.0%) | 0 (0.0%) | 0 (0.0%) |
|  | CS (AE) | 0 (0.0%) | 0 (0.0%) | 0 (0.0%) | 0 (0.0%) | 0 (0.0%) |
| Transition from baseline | Relieved | 0 (0.0%) | 0 (0.0%) | 0 (0.0%) | 0 (0.0%) | 0 (0.0%) |
|  | Unchanged | 6 (100.0%) | 12 (100.0%) | 13 (100.0%) | 12 (100.0%) | 37 (100.0%) |
|  | Worsened (MH) | 0 (0.0%) | 0 (0.0%) | 0 (0.0%) | 0 (0.0%) | 0 (0.0%) |
|  | Worsened (AE) | 0 (0.0%) | 0 (0.0%) | 0 (0.0%) | 0 (0.0%) | 0 (0.0%) |
| **Week 48** | N (Missing) | 6 (0) | 12 (0) | 13 (0) | 12 (0) | 37 (0) |
|  | Mean (SD) | 72.19 (11.935) | 61.00 (14.979) | 72.35 (21.124) | 64.83 (15.748) | 66.23 (17.787) |
|  | Normal | 6 (100.0%) | 12 (100.0%) | 12 (92.3%) | 11 (91.7%) | 35 (94.6%) |
|  | NCS | 0 (0.0%) | 0 (0.0%) | 1 (7.7%) | 1 (8.3%) | 2 (5.4%) |
|  | CS (MH) | 0 (0.0%) | 0 (0.0%) | 0 (0.0%) | 0 (0.0%) | 0 (0.0%) |
|  | CS (AE) | 0 (0.0%) | 0 (0.0%) | 0 (0.0%) | 0 (0.0%) | 0 (0.0%) |
| Transition from baseline | Relieved | 0 (0.0%) | 0 (0.0%) | 0 (0.0%) | 0 (0.0%) | 0 (0.0%) |
|  | Unchanged | 6 (100.0%) | 12 (100.0%) | 13 (100.0%) | 12 (100.0%) | 37 (100.0%) |
|  | Worsened (MH) | 0 (0.0%) | 0 (0.0%) | 0 (0.0%) | 0 (0.0%) | 0 (0.0%) |
|  | Worsened (AE) | 0 (0.0%) | 0 (0.0%) | 0 (0.0%) | 0 (0.0%) | 0 (0.0%) |
| **Week 72** | N (Missing) | 3 (0) | 6 (0) | 7 (0) | 5 (0) | 18 (0) |
|  | Mean (SD) | 76.03 (4.922) | 66.15 (17.480) | 83.86 (31.073) | 61.17 (13.310) | 71.65 (24.012) |
|  | Normal | 3 (100.0%) | 6 (100.0%) | 6 (85.7%) | 5 (100.0%) | 17 (94.4%) |
|  | NCS | 0 (0.0%) | 0 (0.0%) | 1 (14.3%) | 0 (0.0%) | 1 (5.6%) |
|  | CS (MH) | 0 (0.0%) | 0 (0.0%) | 0 (0.0%) | 0 (0.0%) | 0 (0.0%) |
|  | CS (AE) | 0 (0.0%) | 0 (0.0%) | 0 (0.0%) | 0 (0.0%) | 0 (0.0%) |
| Transition from baseline | Relieved | 0 (0.0%) | 0 (0.0%) | 0 (0.0%) | 0 (0.0%) | 0 (0.0%) |
|  | Unchanged | 3 (100.0%) | 6 (100.0%) | 7 (100.0%) | 5 (100.0%) | 18 (100.0%) |
|  | Worsened (MH) | 0 (0.0%) | 0 (0.0%) | 0 (0.0%) | 0 (0.0%) | 0 (0.0%) |
|  | Worsened (AE) | 0 (0.0%) | 0 (0.0%) | 0 (0.0%) | 0 (0.0%) | 0 (0.0%) |
| **Week 96** | N (Missing) | 3 (0) | 7 (0) | 10 (0) | 7 (0) | 24 (0) |
|  | Mean (SD) | 72.49 (14.713) | 64.91 (20.656) | 73.55 (23.104) | 63.65 (10.958) | 68.14 (19.327) |
|  | Normal | 3 (100.0%) | 7 (100.0%) | 8 (80.0%) | 7 (100.0%) | 22 (91.7%) |
|  | NCS | 0 (0.0%) | 0 (0.0%) | 2 (20.0%) | 0 (0.0%) | 2 (8.3%) |
|  | CS (MH) | 0 (0.0%) | 0 (0.0%) | 0 (0.0%) | 0 (0.0%) | 0 (0.0%) |
|  | CS (AE) | 0 (0.0%) | 0 (0.0%) | 0 (0.0%) | 0 (0.0%) | 0 (0.0%) |
| Transition from baseline | Relieved | 0 (0.0%) | 0 (0.0%) | 0 (0.0%) | 0 (0.0%) | 0 (0.0%) |
|  | Unchanged | 3 (100.0%) | 7 (100.0%) | 10 (100.0%) | 7 (100.0%) | 24 (100.0%) |
|  | Worsened (MH) | 0 (0.0%) | 0 (0.0%) | 0 (0.0%) | 0 (0.0%) | 0 (0.0%) |
|  | Worsened (AE) | 0 (0.0%) | 0 (0.0%) | 0 (0.0%) | 0 (0.0%) | 0 (0.0%) |

*NCS: abnormal but not clinically significant, MH: Medical history, AE: Adverse event. Relieved: CS (Medical History or Adverse Event) at baseline to Normal / NCS at visit*

*Unchanged: including Normal at baseline to NCS at visit, NCS at baseline to Normal at visit. Worsened (Medical History): Normal / NCS at baseline to CS (Medical History) at visit. Worsened (Adverse Event): Normal / NCS / CS (Medical History) at baseline to CS (Adverse Event) at visit.*

**Table 14 Summary of Blood Urea Nitrogen [mg/dL] - Safety Population**

| **Blood Urea Nitrogen** | | **HA** | **16M** | **32M** | **64M** | **Pooled** |
| --- | --- | --- | --- | --- | --- | --- |
| **Screening** | N (Missing) | 8 (0) | 17 (0) | 17 (0) | 15 (0) | 49 (0) |
|  | Mean (SD) | 18.54 (5.481) | 15.71 (3.751) | 18.93 (5.768) | 16.21 (3.882) | 16.98 (4.721) |
|  | Groups Diff.  P-value (Wilcox_t) | -- | 0.2327 | 0.9310 | 0.3279 | 0.3739 |
|  | Normal | 6 (75.0%) | 15 (88.2%) | 12 (70.6%) | 13 (86.7%) | 40 (81.6%) |
|  | NCS | 2 (25.0%) | 2 (11.8%) | 5 (29.4%) | 2 (13.3%) | 9 (18.4%) |
|  | CS (MH) | 0 (0.0%) | 0 (0.0%) | 0 (0.0%) | 0 (0.0%) | 0 (0.0%) |
|  | CS (AE) | 0 (0.0%) | 0 (0.0%) | 0 (0.0%) | 0 (0.0%) | 0 (0.0%) |
| **Week 24** | N (Missing) | 8 (0) | 17 (0) | 17 (0) | 15 (0) | 49 (0) |
|  | Mean (SD) | 17.36 (4.084) | 15.20 (4.666) | 18.32 (7.685) | 16.09 (5.066) | 16.56 (6.021) |
|  | Normal | 7 (87.5%) | 16 (94.1%) | 13 (76.5%) | 10 (66.7%) | 39 (79.6%) |
|  | NCS | 1 (12.5%) | 1 (5.9%) | 4 (23.5%) | 5 (33.3%) | 10 (20.4%) |
|  | CS (MH) | 0 (0.0%) | 0 (0.0%) | 0 (0.0%) | 0 (0.0%) | 0 (0.0%) |
|  | CS (AE) | 0 (0.0%) | 0 (0.0%) | 0 (0.0%) | 0 (0.0%) | 0 (0.0%) |
| Transition from baseline | Relieved | 0 (0.0%) | 0 (0.0%) | 0 (0.0%) | 0 (0.0%) | 0 (0.0%) |
|  | Unchanged | 8 (100.0%) | 17 (100.0%) | 17 (100.0%) | 15 (100.0%) | 49 (100.0%) |
|  | Worsened (MH) | 0 (0.0%) | 0 (0.0%) | 0 (0.0%) | 0 (0.0%) | 0 (0.0%) |
|  | Worsened (AE) | 0 (0.0%) | 0 (0.0%) | 0 (0.0%) | 0 (0.0%) | 0 (0.0%) |
| **Week 36** | N (Missing) | 6 (0) | 12 (0) | 13 (0) | 12 (2) | 37 (2) |
|  | Mean (SD) | 16.27 (4.027) | 14.37 (2.579) | 17.09 (5.577) | 15.04 (4.773) | 15.54 (4.558) |
|  | Normal | 5 (83.3%) | 12 (100.0%) | 11 (84.6%) | 11 (91.7%) | 34 (91.9%) |
|  | NCS | 1 (16.7%) | 0 (0.0%) | 2 (15.4%) | 1 (8.3%) | 3 (8.1%) |
|  | CS (MH) | 0 (0.0%) | 0 (0.0%) | 0 (0.0%) | 0 (0.0%) | 0 (0.0%) |
|  | CS (AE) | 0 (0.0%) | 0 (0.0%) | 0 (0.0%) | 0 (0.0%) | 0 (0.0%) |
| Transition from baseline | Relieved | 0 (0.0%) | 0 (0.0%) | 0 (0.0%) | 0 (0.0%) | 0 (0.0%) |
|  | Unchanged | 6 (100.0%) | 12 (100.0%) | 13 (100.0%) | 12 (100.0%) | 37 (100.0%) |
|  | Worsened (MH) | 0 (0.0%) | 0 (0.0%) | 0 (0.0%) | 0 (0.0%) | 0 (0.0%) |
|  | Worsened (AE) | 0 (0.0%) | 0 (0.0%) | 0 (0.0%) | 0 (0.0%) | 0 (0.0%) |
| **Week 48** | N (Missing) | 6 (0) | 12 (0) | 13 (0) | 12 (0) | 37 (0) |
|  | Mean (SD) | 15.55 (4.248) | 15.62 (3.401) | 16.36 (3.331) | 16.02 (4.971) | 16.01 (3.857) |
|  | Normal | 5 (83.3%) | 10 (83.3%) | 11 (84.6%) | 9 (75.0%) | 30 (81.1%) |
|  | NCS | 1 (16.7%) | 2 (16.7%) | 2 (15.4%) | 3 (25.0%) | 7 (18.9%) |
|  | CS (MH) | 0 (0.0%) | 0 (0.0%) | 0 (0.0%) | 0 (0.0%) | 0 (0.0%) |
|  | CS (AE) | 0 (0.0%) | 0 (0.0%) | 0 (0.0%) | 0 (0.0%) | 0 (0.0%) |
| Transition from baseline | Relieved | 0 (0.0%) | 0 (0.0%) | 0 (0.0%) | 0 (0.0%) | 0 (0.0%) |
|  | Unchanged | 6 (100.0%) | 12 (100.0%) | 13 (100.0%) | 12 (100.0%) | 37 (100.0%) |
|  | Worsened (MH) | 0 (0.0%) | 0 (0.0%) | 0 (0.0%) | 0 (0.0%) | 0 (0.0%) |
|  | Worsened (AE) | 0 (0.0%) | 0 (0.0%) | 0 (0.0%) | 0 (0.0%) | 0 (0.0%) |
| **Week 72** | N (Missing) | 3 (0) | 6 (0) | 7 (0) | 5 (0) | 18 (0) |
|  | Mean (SD) | 14.50 (3.041) | 15.12 (2.807) | 18.07 (4.219) | 14.22 (4.104) | 16.02 (3.942) |
|  | Normal | 3 (100.0%) | 6 (100.0%) | 5 (71.4%) | 5 (100.0%) | 16 (88.9%) |
|  | NCS | 0 (0.0%) | 0 (0.0%) | 2 (28.6%) | 0 (0.0%) | 2 (11.1%) |
|  | CS (MH) | 0 (0.0%) | 0 (0.0%) | 0 (0.0%) | 0 (0.0%) | 0 (0.0%) |
|  | CS (AE) | 0 (0.0%) | 0 (0.0%) | 0 (0.0%) | 0 (0.0%) | 0 (0.0%) |
| Transition from baseline | Relieved | 0 (0.0%) | 0 (0.0%) | 0 (0.0%) | 0 (0.0%) | 0 (0.0%) |
|  | Unchanged | 3 (100.0%) | 6 (100.0%) | 7 (100.0%) | 5 (100.0%) | 18 (100.0%) |
|  | Worsened (MH) | 0 (0.0%) | 0 (0.0%) | 0 (0.0%) | 0 (0.0%) | 0 (0.0%) |
|  | Worsened (AE) | 0 (0.0%) | 0 (0.0%) | 0 (0.0%) | 0 (0.0%) | 0 (0.0%) |
| **Week 96** | N (Missing) | 3 (0) | 7 (0) | 10 (0) | 7 (0) | 24 (0) |
|  | Mean (SD) | 17.67 (2.309) | 14.70 (4.097) | 17.36 (5.093) | 14.93 (5.675) | 15.88 (4.958) |
|  | Normal | 3 (100.0%) | 7 (100.0%) | 8 (80.0%) | 6 (85.7%) | 21 (87.5%) |
|  | NCS | 0 (0.0%) | 0 (0.0%) | 2 (20.0%) | 1 (14.3%) | 3 (12.5%) |
|  | CS (MH) | 0 (0.0%) | 0 (0.0%) | 0 (0.0%) | 0 (0.0%) | 0 (0.0%) |
|  | CS (AE) | 0 (0.0%) | 0 (0.0%) | 0 (0.0%) | 0 (0.0%) | 0 (0.0%) |
| Transition from baseline | Relieved | 0 (0.0%) | 0 (0.0%) | 0 (0.0%) | 0 (0.0%) | 0 (0.0%) |
|  | Unchanged | 3 (100.0%) | 7 (100.0%) | 10 (100.0%) | 7 (100.0%) | 24 (100.0%) |
|  | Worsened (MH) | 0 (0.0%) | 0 (0.0%) | 0 (0.0%) | 0 (0.0%) | 0 (0.0%) |
|  | Worsened (AE) | 0 (0.0%) | 0 (0.0%) | 0 (0.0%) | 0 (0.0%) | 0 (0.0%) |

*NCS: abnormal but not clinically significant, MH: Medical history, AE: Adverse event. Relieved: CS (Medical History or Adverse Event) at baseline to Normal / NCS at visit*

*Unchanged: including Normal at baseline to NCS at visit, NCS at baseline to Normal at visit. Worsened (Medical History): Normal / NCS at baseline to CS (Medical History) at visit. Worsened (Adverse Event): Normal / NCS / CS (Medical History) at baseline to CS (Adverse Event) at visit.*

**Table 15 Summary of Albumin [g/dL] - Safety Population**

| **Albumin** | | **HA** | **16M** | **32M** | **64M** | **Pooled** |
| --- | --- | --- | --- | --- | --- | --- |
| **Screening** | N (Missing) | 8 (0) | 17 (0) | 17 (0) | 15 (0) | 49 (0) |
|  | Mean (SD) | 4.22 (0.260) | 4.39 (0.243) | 4.29 (0.324) | 4.53 (0.244) | 4.40 (0.286) |
|  | Groups Diff.  P-value (Wilcox_t) | -- | 0.1575 | 0.4554 | 0.0317 | 0.0922 |
|  | Normal | 8 (100.0%) | 17 (100.0%) | 16 (94.1%) | 15 (100.0%) | 48 (98.0%) |
|  | NCS | 0 (0.0%) | 0 (0.0%) | 1 (5.9%) | 0 (0.0%) | 1 (2.0%) |
|  | CS (MH) | 0 (0.0%) | 0 (0.0%) | 0 (0.0%) | 0 (0.0%) | 0 (0.0%) |
|  | CS (AE) | 0 (0.0%) | 0 (0.0%) | 0 (0.0%) | 0 (0.0%) | 0 (0.0%) |
| **Week 24** | N (Missing) | 8 (0) | 17 (0) | 17 (0) | 15 (0) | 49 (0) |
|  | Mean (SD) | 4.26 (0.143) | 4.30 (0.220) | 4.27 (0.302) | 4.41 (0.208) | 4.32 (0.250) |
|  | Normal | 8 (100.0%) | 17 (100.0%) | 16 (94.1%) | 15 (100.0%) | 48 (98.0%) |
|  | NCS | 0 (0.0%) | 0 (0.0%) | 1 (5.9%) | 0 (0.0%) | 1 (2.0%) |
|  | CS (MH) | 0 (0.0%) | 0 (0.0%) | 0 (0.0%) | 0 (0.0%) | 0 (0.0%) |
|  | CS (AE) | 0 (0.0%) | 0 (0.0%) | 0 (0.0%) | 0 (0.0%) | 0 (0.0%) |
| Transition from baseline | Relieved | 0 (0.0%) | 0 (0.0%) | 0 (0.0%) | 0 (0.0%) | 0 (0.0%) |
|  | Unchanged | 8 (100.0%) | 17 (100.0%) | 17 (100.0%) | 15 (100.0%) | 49 (100.0%) |
|  | Worsened (MH) | 0 (0.0%) | 0 (0.0%) | 0 (0.0%) | 0 (0.0%) | 0 (0.0%) |
|  | Worsened (AE) | 0 (0.0%) | 0 (0.0%) | 0 (0.0%) | 0 (0.0%) | 0 (0.0%) |
| **Week 36** | N (Missing) | 6 (0) | 12 (0) | 13 (0) | 12 (2) | 37 (2) |
|  | Mean (SD) | 4.24 (0.176) | 4.24 (0.202) | 4.29 (0.282) | 4.36 (0.259) | 4.29 (0.249) |
|  | Normal | 6 (100.0%) | 12 (100.0%) | 13 (100.0%) | 12 (100.0%) | 37 (100.0%) |
|  | NCS | 0 (0.0%) | 0 (0.0%) | 0 (0.0%) | 0 (0.0%) | 0 (0.0%) |
|  | CS (MH) | 0 (0.0%) | 0 (0.0%) | 0 (0.0%) | 0 (0.0%) | 0 (0.0%) |
|  | CS (AE) | 0 (0.0%) | 0 (0.0%) | 0 (0.0%) | 0 (0.0%) | 0 (0.0%) |
| Transition from baseline | Relieved | 0 (0.0%) | 0 (0.0%) | 0 (0.0%) | 0 (0.0%) | 0 (0.0%) |
|  | Unchanged | 6 (100.0%) | 12 (100.0%) | 13 (100.0%) | 12 (100.0%) | 37 (100.0%) |
|  | Worsened (MH) | 0 (0.0%) | 0 (0.0%) | 0 (0.0%) | 0 (0.0%) | 0 (0.0%) |
|  | Worsened (AE) | 0 (0.0%) | 0 (0.0%) | 0 (0.0%) | 0 (0.0%) | 0 (0.0%) |
| **Week 48** | N (Missing) | 6 (0) | 12 (0) | 13 (0) | 12 (0) | 37 (0) |
|  | Mean (SD) | 4.23 (0.188) | 4.27 (0.184) | 4.32 (0.187) | 4.38 (0.141) | 4.32 (0.174) |
|  | Normal | 6 (100.0%) | 12 (100.0%) | 13 (100.0%) | 12 (100.0%) | 37 (100.0%) |
|  | NCS | 0 (0.0%) | 0 (0.0%) | 0 (0.0%) | 0 (0.0%) | 0 (0.0%) |
|  | CS (MH) | 0 (0.0%) | 0 (0.0%) | 0 (0.0%) | 0 (0.0%) | 0 (0.0%) |
|  | CS (AE) | 0 (0.0%) | 0 (0.0%) | 0 (0.0%) | 0 (0.0%) | 0 (0.0%) |
| Transition from baseline | Relieved | 0 (0.0%) | 0 (0.0%) | 0 (0.0%) | 0 (0.0%) | 0 (0.0%) |
|  | Unchanged | 6 (100.0%) | 12 (100.0%) | 13 (100.0%) | 12 (100.0%) | 37 (100.0%) |
|  | Worsened (MH) | 0 (0.0%) | 0 (0.0%) | 0 (0.0%) | 0 (0.0%) | 0 (0.0%) |
|  | Worsened (AE) | 0 (0.0%) | 0 (0.0%) | 0 (0.0%) | 0 (0.0%) | 0 (0.0%) |
| **Week 72** | N (Missing) | 3 (0) | 6 (0) | 7 (0) | 5 (0) | 18 (0) |
|  | Mean (SD) | 4.09 (0.110) | 4.48 (0.210) | 4.26 (0.194) | 4.31 (0.316) | 4.35 (0.245) |
|  | Normal | 3 (100.0%) | 6 (100.0%) | 7 (100.0%) | 5 (100.0%) | 18 (100.0%) |
|  | NCS | 0 (0.0%) | 0 (0.0%) | 0 (0.0%) | 0 (0.0%) | 0 (0.0%) |
|  | CS (MH) | 0 (0.0%) | 0 (0.0%) | 0 (0.0%) | 0 (0.0%) | 0 (0.0%) |
|  | CS (AE) | 0 (0.0%) | 0 (0.0%) | 0 (0.0%) | 0 (0.0%) | 0 (0.0%) |
| Transition from baseline | Relieved | 0 (0.0%) | 0 (0.0%) | 0 (0.0%) | 0 (0.0%) | 0 (0.0%) |
|  | Unchanged | 3 (100.0%) | 6 (100.0%) | 7 (100.0%) | 5 (100.0%) | 18 (100.0%) |
|  | Worsened (MH) | 0 (0.0%) | 0 (0.0%) | 0 (0.0%) | 0 (0.0%) | 0 (0.0%) |
|  | Worsened (AE) | 0 (0.0%) | 0 (0.0%) | 0 (0.0%) | 0 (0.0%) | 0 (0.0%) |
| **Week 96** | N (Missing) | 3 (0) | 7 (0) | 10 (0) | 7 (0) | 24 (0) |
|  | Mean (SD) | 4.16 (0.051) | 4.39 (0.313) | 4.32 (0.224) | 4.35 (0.273) | 4.35 (0.256) |
|  | Normal | 3 (100.0%) | 7 (100.0%) | 10 (100.0%) | 7 (100.0%) | 24 (100.0%) |
|  | NCS | 0 (0.0%) | 0 (0.0%) | 0 (0.0%) | 0 (0.0%) | 0 (0.0%) |
|  | CS (MH) | 0 (0.0%) | 0 (0.0%) | 0 (0.0%) | 0 (0.0%) | 0 (0.0%) |
|  | CS (AE) | 0 (0.0%) | 0 (0.0%) | 0 (0.0%) | 0 (0.0%) | 0 (0.0%) |
| Transition from baseline | Relieved | 0 (0.0%) | 0 (0.0%) | 0 (0.0%) | 0 (0.0%) | 0 (0.0%) |
|  | Unchanged | 3 (100.0%) | 7 (100.0%) | 10 (100.0%) | 7 (100.0%) | 24 (100.0%) |
|  | Worsened (MH) | 0 (0.0%) | 0 (0.0%) | 0 (0.0%) | 0 (0.0%) | 0 (0.0%) |
|  | Worsened (AE) | 0 (0.0%) | 0 (0.0%) | 0 (0.0%) | 0 (0.0%) | 0 (0.0%) |

*NCS: abnormal but not clinically significant, MH: Medical history, AE: Adverse event. Relieved: CS (Medical History or Adverse Event) at baseline to Normal / NCS at visit*

*Unchanged: including Normal at baseline to NCS at visit, NCS at baseline to Normal at visit. Worsened (Medical History): Normal / NCS at baseline to CS (Medical History) at visit. Worsened (Adverse Event): Normal / NCS / CS (Medical History) at baseline to CS (Adverse Event) at visit.*

**Table 16 Summary of Immunogenicity – CD4/Lymphocyte [%]**

| **CD4/ lymphocyte [%]** | | **HA** | **16M** | **32M** | **64M** | **Pooled** |
| --- | --- | --- | --- | --- | --- | --- |
| **Screening** | N (Missing) | 6 (0) | 17 (0) | 17 (0) | 11 (0) | 45 (0) |
|  | Mean (SD) | 39.68 (11.514) | 39.75 (13.508) | 41.64 (9.393) | 39.32 (6.708) | 40.36 (10.474) |
|  | Groups diff.  P-value (T test) | -- | 0.9903 | 0.6822 | 0.9361 | 0.8828 |
|  | Normal | 5 (83.3%) | 13 (76.5%) | 15 (88.2%) | 11 (100.0%) | 39 (86.7%) |
|  | NCS | 1 (16.7%) | 4 (23.5%) | 2 (11.8%) | 0 (0.0%) | 6 (13.3%) |
|  | CS (MH) | 0 (0.0%) | 0 (0.0%) | 0 (0.0%) | 0 (0.0%) | 0 (0.0%) |
|  | CS (AE) | 0 (0.0%) | 0 (0.0%) | 0 (0.0%) | 0 (0.0%) | 0 (0.0%) |
| **Week 4** | N (Missing) | 8 (0) | 16 (0) | 17 (0) | 14 (0) | 47 (0) |
|  | Mean (SD) | 38.91 (10.404) | 43.12 (9.462) | 39.41 (10.336) | 41.11 (6.678) | 41.18 (9.024) |
|  | Normal | 7 (87.5%) | 13 (81.3%) | 14 (82.4%) | 13 (92.9%) | 40 (85.1%) |
|  | NCS | 1 (12.5%) | 3 (18.8%) | 3 (17.6%) | 1 (7.1%) | 7 (14.9%) |
|  | CS (MH) | 0 (0.0%) | 0 (0.0%) | 0 (0.0%) | 0 (0.0%) | 0 (0.0%) |
|  | CS (AE) | 0 (0.0%) | 0 (0.0%) | 0 (0.0%) | 0 (0.0%) | 0 (0.0%) |
| Transition from baseline | Relieved | 0 (0.0%) | 0 (0.0%) | 0 (0.0%) | 0 (0.0%) | 0 (0.0%) |
|  | Unchanged | 6 (100.0%) | 16 (100.0%) | 17 (100.0%) | 10 (100.0%) | 43 (100.0%) |
|  | Worsened (MH) | 0 (0.0%) | 0 (0.0%) | 0 (0.0%) | 0 (0.0%) | 0 (0.0%) |
|  | Worsened (AE) | 0 (0.0%) | 0 (0.0%) | 0 (0.0%) | 0 (0.0%) | 0 (0.0%) |
| **Week 24** | N (Missing) | 8 (0) | 17 (0) | 17 (0) | 15 (0) | 49 (0) |
|  | Mean (SD) | 37.53 (13.218) | 42.58  (8.633) | 39.23 (10.065) | 41.00  (6.775) | 40.94  (8.602) |
|  | Normal | 6 (75.0%) | 15 (88.2%) | 13 (76.5%) | 15 (100.0%) | 43 (87.8%) |
|  | NCS | 2 (25.0%) | 2 (11.8%) | 4 (23.5%) | 0 (0.0%) | 6 (12.2%) |
|  | CS (MH) | 0 (0.0%) | 0 (0.0%) | 0 (0.0%) | 0 (0.0%) | 0 (0.0%) |
|  | CS (AE) | 0 (0.0%) | 0 (0.0%) | 0 (0.0%) | 0 (0.0%) | 0 (0.0%) |
| Transition from baseline | Relieved | 0 (0.0%) | 0 (0.0%) | 0 (0.0%) | 0 (0.0%) | 0 (0.0%) |
|  | Unchanged | 6 (100.0%) | 17 (100.0%) | 17 (100.0%) | 11 (100.0%) | 45 (100.0%) |
|  | Worsened (MH) | 0 (0.0%) | 0 (0.0%) | 0 (0.0%) | 0 (0.0%) | 0 (0.0%) |
|  | Worsened (AE) | 0 (0.0%) | 0 (0.0%) | 0 (0.0%) | 0 (0.0%) | 0 (0.0%) |

*NCS: abnormal but not clinically significant, MH: Medical history, AE: Adverse event. Relieved: CS (Medical History or Adverse Event) at baseline to Normal / NCS at visit*

*Unchanged: including Normal at baseline to NCS at visit, NCS at baseline to Normal at visit. Worsened (Medical History): Normal / NCS at baseline to CS (Medical History) at visit. Worsened (Adverse Event): Normal / NCS / CS (Medical History) at baseline to CS (Adverse Event) at visit.*

**Table 17 Summary of Immunogenicity – CD8/Lymphocyte [%]**

| **CD8/Lymphocyte [%]** | | **HA** | **16M** | **32M** | **64M** | **Pooled** |
| --- | --- | --- | --- | --- | --- | --- |
| **Screening** | N (Missing) | 6 (0) | 17 (0) | 17 (0) | 11 (0) | 45 (0) |
|  | Mean (SD) | 22.74 (5.840) | 21.53 (5.822) | 24.27 (10.077) | 23.14 (8.750) | 22.96 (8.253) |
|  | Groups diff.  P-value (Wilcox_t) | -- | 0.7039 | 1.0000 | 1.0000 | 0.8729 |
|  | Normal | 5 (83.3%) | 12 (70.6%) | 10 (58.8%) | 8 (72.7%) | 30 (66.7%) |
|  | NCS | 1 (16.7%) | 5 (29.4%) | 7 (41.2%) | 3 (27.3%) | 15 (33.3%) |
|  | CS (MH) | 0 (0.0%) | 0 (0.0%) | 0 (0.0%) | 0 (0.0%) | 0 (0.0%) |
|  | CS (AE) | 0 (0.0%) | 0 (0.0%) | 0 (0.0%) | 0 (0.0%) | 0 (0.0%) |
| **Week 4** | N (Missing) | 8 (0) | 16 (0) | 17 (0) | 14 (0) | 47 (0) |
|  | Mean (SD) | 25.22 (6.620) | 22.10 (6.785) | 25.32 (10.831) | 22.66 (9.118) | 23.43 (9.023) |
|  | Normal | 7 (87.5%) | 11 (68.8%) | 11 (64.7%) | 11 (78.6%) | 33 (70.2%) |
|  | NCS | 1 (12.5%) | 5 (31.3%) | 6 (35.3%) | 3 (21.4%) | 14 (29.8%) |
|  | CS (MH) | 0 (0.0%) | 0 (0.0%) | 0 (0.0%) | 0 (0.0%) | 0 (0.0%) |
|  | CS (AE) | 0 (0.0%) | 0 (0.0%) | 0 (0.0%) | 0 (0.0%) | 0 (0.0%) |
| Transition from baseline | Relieved | 0 (0.0%) | 0 (0.0%) | 0 (0.0%) | 0 (0.0%) | 0 (0.0%) |
|  | Unchanged | 6 (100.0%) | 16 (100.0%) | 17 (100.0%) | 10 (100.0%) | 43 (100.0%) |
|  | Worsened (MH) | 0 (0.0%) | 0 (0.0%) | 0 (0.0%) | 0 (0.0%) | 0 (0.0%) |
|  | Worsened (AE) | 0 (0.0%) | 0 (0.0%) | 0 (0.0%) | 0 (0.0%) | 0 (0.0%) |
| **Week 24** | N (Missing) | 8 (0) | 17 (0) | 17 (0) | 15 (0) | 49 (0) |
|  | Mean (SD) | 23.47 (7.098) | 23.89 (7.068) | 24.98 (10.701) | 22.97 (7.366) | 23.99 (8.445) |
|  | Normal | 6 (75.0%) | 14 (82.4%) | 11 (64.7%) | 12 (80.0%) | 37 (75.5%) |
|  | NCS | 2 (25.0%) | 3 (17.6%) | 6 (35.3%) | 3 (20.0%) | 12 (24.5%) |
|  | CS (MH) | 0 (0.0%) | 0 (0.0%) | 0 (0.0%) | 0 (0.0%) | 0 (0.0%) |
|  | CS (AE) | 0 (0.0%) | 0 (0.0%) | 0 (0.0%) | 0 (0.0%) | 0 (0.0%) |
| Transition from baseline | Relieved | 0 (0.0%) | 0 (0.0%) | 0 (0.0%) | 0 (0.0%) | 0 (0.0%) |
|  | Unchanged | 6 (100.0%) | 17 (100.0%) | 17 (100.0%) | 11 (100.0%) | 45 (100.0%) |
|  | Worsened (MH) | 0 (0.0%) | 0 (0.0%) | 0 (0.0%) | 0 (0.0%) | 0 (0.0%) |
|  | Worsened (AE) | 0 (0.0%) | 0 (0.0%) | 0 (0.0%) | 0 (0.0%) | 0 (0.0%) |

*NCS: abnormal but not clinically significant, MH: Medical history, AE: Adverse event. Relieved: CS (Medical History or Adverse Event) at baseline to Normal / NCS at visit*

*Unchanged: including Normal at baseline to NCS at visit, NCS at baseline to Normal at visit. Worsened (Medical History): Normal / NCS at baseline to CS (Medical History) at visit. Worsened (Adverse Event): Normal / NCS / CS (Medical History) at baseline to CS (Adverse Event) at visit.*

**Table 18 Summary of Immunogenicity – Tumor Necrosis Factor Alpha [ng/L]**

| **Tumor Necrosis Factor [ng/L]** | | **HA** | **16M** | **32M** | **64M** | **Pooled** |
| --- | --- | --- | --- | --- | --- | --- |
| **Screening** | N (Missing) | 6 (0) | 17 (0) | 17 (0) | 11 (0) | 45 (0) |
|  | Mean (SD) | 7.39 (2.757) | 7.84 (3.604) | 8.73 (2.808) | 8.46 (2.573) | 8.33 (3.042) |
|  | Groups diff.  P-value (Wilcox_t) | -- | 0.9448 | 0.4293 | 0.3539 | 0.5613 |
|  | Normal | 4 (66.7%) | 10 (58.8%) | 7 (41.2%) | 5 (45.5%) | 22 (48.9%) |
|  | NCS | 2 (33.3%) | 7 (41.2%) | 10 (58.8%) | 6 (54.5%) | 23 (51.1%) |
|  | CS (MH) | 0 (0.0%) | 0 (0.0%) | 0 (0.0%) | 0 (0.0%) | 0 (0.0%) |
|  | CS (AE) | 0 (0.0%) | 0 (0.0%) | 0 (0.0%) | 0 (0.0%) | 0 (0.0%) |
| **Week 4** | N (Missing) | 8 (0) | 16 (0) | 17 (0) | 14 (0) | 47 (0) |
|  | Mean (SD) | 7.22  (1.983) | 7.50  (3.157) | 10.32 (6.294) | 7.43  (3.079) | 8.50  (4.650) |
|  | Normal | 4 (50.0%) | 12 (75.0%) | 7 (41.2%) | 8 (57.1%) | 27 (57.4%) |
|  | NCS | 4 (50.0%) | 4 (25.0%) | 9 (52.9%) | 6 (42.9%) | 19 (40.4%) |
|  | CS (MH) | 0 (0.0%) | 0 (0.0%) | 0 (0.0%) | 0 (0.0%) | 0 (0.0%) |
|  | CS (AE) | 0 (0.0%) | 0 (0.0%) | 1 (5.9%) | 0 (0.0%) | 1 (2.1%) |
| Transition from baseline | Relieved | 0 (0.0%) | 0 (0.0%) | 0 (0.0%) | 0 (0.0%) | 0 (0.0%) |
|  | Unchanged | 6 (100.0%) | 16 (100.0%) | 16 (94.1%) | 10 (100.0%) | 42 (97.7%) |
|  | Worsened (MH) | 0 (0.0%) | 0 (0.0%) | 0 (0.0%) | 0 (0.0%) | 0 (0.0%) |
|  | Worsened (AE) | 0 (0.0%) | 0 (0.0%) | 1 (5.9%) | 0 (0.0%) | 1 (2.3%) |
| **Week 24** | N (Missing) | 8 (0) | 17 (0) | 17 (0) | 15 (0) | 49 (0) |
|  | Mean (SD) | 7.38 (2.360) | 7.42 (3.307) | 7.45 (2.470) | 7.11 (2.990) | 7.34 (2.883) |
|  | Normal | 6 (75.0%) | 14 (82.4%) | 12 (70.6%) | 11 (73.3%) | 37 (75.5%) |
|  | NCS | 2 (25.0%) | 3 (17.6%) | 5 (29.4%) | 4 (26.7%) | 12 (24.5%) |
|  | CS (MH) | 0 (0.0%) | 0 (0.0%) | 0 (0.0%) | 0 (0.0%) | 0 (0.0%) |
|  | CS (AE) | 0 (0.0%) | 0 (0.0%) | 0 (0.0%) | 0 (0.0%) | 0 (0.0%) |
| Transition from baseline | Relieved | 0 (0.0%) | 0 (0.0%) | 0 (0.0%) | 0 (0.0%) | 0 (0.0%) |
|  | Unchanged | 6 (100.0%) | 17 (100.0%) | 17 (100.0%) | 11 (100.0%) | 45 (100.0%) |
|  | Worsened (MH) | 0 (0.0%) | 0 (0.0%) | 0 (0.0%) | 0 (0.0%) | 0 (0.0%) |
|  | Worsened (AE) | 0 (0.0%) | 0 (0.0%) | 0 (0.0%) | 0 (0.0%) | 0 (0.0%) |

*NCS: abnormal but not clinically significant, MH: Medical history, AE: Adverse event. Relieved: CS (Medical History or Adverse Event) at baseline to Normal / NCS at visit*

*Unchanged: including Normal at baseline to NCS at visit, NCS at baseline to Normal at visit. Worsened (Medical History): Normal / NCS at baseline to CS (Medical History) at visit. Worsened (Adverse Event): Normal / NCS / CS (Medical History) at baseline to CS (Adverse Event) at visit.*
