# Supplementary material for: Treatment of knee osteoarthritis with intra-articular injection of allogeneic adipose-derived stem cells (ADSCs) ELIXCYTE®: a phase I/II, randomized, active-control, single-blind, multiple-center clinical trial
Source: Stem Cell Res Ther. 2021 Oct 30;12:562. doi: 10.1186/s13287-021-02631-z (PMC8557559; doi:10.1186/s13287-021-02631-z)
Supplement: Supplementary file 2 — Additional file 2: Supplementary safety and efficacy data of ELIXCYTE® including Table S1-S12. [file 13287_2021_2631_MOESM2_ESM.docx]

**Supplementary safety and efficacy data of ELIXCYTE^®^**

**Table S1**

**Summary of Adverse Events during Week 24 – 96 (Long-term TEAEs)**

| **Long-Term AE (Week 24 - 96)** | **HA**  **N=8** | | **16M**  **N=17** | | **32M**  **N=17** | | **64M**  **N=15** | | **Total**  **N=57** | |
| --- | --- | --- | --- | --- | --- | --- | --- | --- | --- | --- |
| **[Event # (E)/Subject # (S) (%)]** | **E** | **S (%)** | **E** | **S (%)** | **E** | **S (%)** | **E** | **S (%)** | **E** | **S (%)** |
| **At least one below [Event#:Subj#]** | **16** | **7 (87.5%)** | **19** | **7 (41.2%)** | **23** | **8 (47.1%)** | **25** | **9 (60.0%)** | **83** | **31 (54.4%)** |
| Ear and labyrinth disorders | 0 | 0 (0.0%) | 3 | 1 (5.9%) | 1 | 1 (5.9%) | 0 | 0 (0.0%) | 4 | 2 (3.5%) |
| Eye disorders | 2 | 2 (25.0%) | 0 | 0 (0.0%) | 0 | 0 (0.0%) | 0 | 0 (0.0%) | 2 | 2 (3.5%) |
| Gastrointestinal disorders | 2 | 1 (12.5%) | 1 | 1 (5.9%) | 3 | 2 (11.8%) | 1 | 1 (6.7%) | 7 | 5 (8.8%) |
| General disorders and administration site conditions | 0 | 0 (0.0%) | 0 | 0 (0.0%) | 1 | 1 (5.9%) | 0 | 0 (0.0%) | 1 | 1 (1.8%) |
| Infections and infestations | 1 | 1 (12.5%) | 6 | 4 (23.5%) | 3 | 2 (11.8%) | 9 | 5 (33.3%) | 19 | 12 (21.1%) |
| Injury, poisoning and procedural complications | 3 | 2 (25.0%) | 1 | 1 (5.9%) | 2 | 2 (11.8%) | 2 | 1 (6.7%) | 8 | 6 (10.5%) |
| Investigations | 0 | 0 (0.0%) | 0 | 0 (0.0%) | 0 | 0 (0.0%) | 3 | 2 (13.3%) | 3 | 2 (3.5%) |
| Metabolism and nutrition disorders | 0 | 0 (0.0%) | 1 | 1 (5.9%) | 1 | 1 (5.9%) | 0 | 0 (0.0%) | 2 | 2 (3.5%) |
| Musculoskeletal and connective tissue disorders | 4 | 3 (37.5%) | 4 | 3 (17.6%) | 4 | 3 (17.6%) | 4 | 4 (26.7%) | 16 | 13 (22.8%) |
| Neoplasms benign, malignant and unspecified (including cysts and polyps) | 0 | 0 (0.0%) | 0 | 0 (0.0%) | 0 | 0 (0.0%) | 2 | 2 (13.3%) | 2 | 2 (3.5%) |
| Nervous system disorders | 1 | 1 (12.5%) | 0 | 0 (0.0%) | 3 | 3 (17.6%) | 0 | 0 (0.0%) | 4 | 4 (7.0%) |
| Psychiatric disorders | 1 | 1 (12.5%) | 0 | 0 (0.0%) | 1 | 1 (5.9%) | 0 | 0 (0.0%) | 2 | 2 (3.5%) |
| Renal and urinary disorders | 0 | 0 (0.0%) | 1 | 1 (5.9%) | 2 | 2 (11.8%) | 0 | 0 (0.0%) | 3 | 3 (5.3%) |
| Reproductive system and breast disorders | 1 | 1 (12.5%) | 0 | 0 (0.0%) | 2 | 1 (5.9%) | 0 | 0 (0.0%) | 3 | 2 (3.5%) |
| Respiratory, thoracic and mediastinal disorders | 1 | 1 (12.5%) | 1 | 1 (5.9%) | 0 | 0 (0.0%) | 2 | 2 (13.3%) | 4 | 4 (7.0%) |
| Skin and subcutaneous tissue disorders | 0 | 0 (0.0%) | 0 | 0 (0.0%) | 0 | 0 (0.0%) | 2 | 2 (13.3%) | 2 | 2 (3.5%) |
| Vascular disorders | 0 | 0 (0.0%) | 1 | 1 (5.9%) | 0 | 0 (0.0%) | 0 | 0 (0.0%) | 1 | 1 (1.8%) |

*Dictionary: MedDRA version 19.0*

**Table S2**

**Summary of WOMAC Pain Score**

| **WOMAC_Pain Score** | | | | | |
| --- | --- | --- | --- | --- | --- |
| **Groups** | **HA** | **16M** | **32M** | **64M** | **Pooled** |
| **Baseline** | | | | | |
| N (Missing) | 8 (0) | 17 (0) | 17 (0) | 15 (0) | 49 (0) |
| Mean (SD) | 8.88 | 9.29 | 10.24 (3.192) | 8.67 | 9.43 |
|  | -1.959 | -2.953 |  | -2.743 | -2.986 |
| Median (IQR) | 8.50 (3.50) | 8.00 (5.00) | 8.00 (4.00) | 7.00 (3.00) | 8.00 (5.00) |
| Groups Diff. | -- | 1 | 0.3355 | 0.4929 | 0.8772 |
| *p*-value (Wilcox_t) |  |  |  |  |  |
| **Week 2 – Baseline** | | | | | |
| N (Missing) | 8 (0) | 17 (0) | 16 (0) | 15 (0) | 48 (0) |
| Mean (SD) | -0.88 | -3.35 | -3.06 | -2.27 | -2.92 |
|  | -2.167 | -4.015 | -3.605 | -5.391 | -4.302 |
| Median (IQR) | -0.50 (3.00) | -4.00 (3.00) | -4.00 (3.50) | -4.00 (8.00) | -4.00 (4.50) |
| 95% CI (WI) | -2.687 | -5 | -4.983 | -5.252 | -5 |
|  | ~ 0.937t | ~ -2.000w | ~ -1.142t | ~ 0.719t | ~ -2.000w |
| *p*-value (WI) | 0.2910t | 0.0024w* | 0.0040t* | 0.1257t | <.0001w* |
| Groups Diff. (Student t) | -- | -5.533 | -4.946 | -4.888 | -4.688 |
| 95% CI of LsMean |  | ~ 1.077 | ~ 1.766 | ~ 1.856 | ~ 1.103 |
| *p*-value (LsMean) | -- | 0.182 | 0.3459 | 0.3709 | 0.2199 |
|  | ANCOVA1 (type 3 *p*-value): Model (0.0241*) with effect Treatment (0.6107), Baseline (0.0030*) | | | | |
|  | ANCOVA2 (type 3 *p*-value): Model (0.0039*) with effect Treatment (0.2199), Baseline (0.0022*) | | | | |
| **Week 4 – Baseline** | | | | | |
| N (Missing) | 8 (0) | 17 (0) | 17 (0) | 15 (0) | 49 (0) |
| Mean (SD) | -2 | -3.35 | -4.06 | -4.93 | -4.08 |
|  | -2.39 | -2.262 | -2.512 | -2.89 | -2.581 |
| Median (IQR) | -2.00 (3.50) | -3.00 (3.00) | -4.00 (2.00) | -6.00 (5.00) | -4.00 (4.00) |
| 95% CI (WI) | -3.998 | -4.516 | -5.35 | -6.534 | -4.823 |
|  | ~ -0.002t | ~ -2.190t | ~ -2.767t | ~ -3.333t | ~ -3.340t |
| *p*-value (WI) | 0.0499t* | <.0001t* | <.0001t* | <.0001t* | <.0001t* |
| Groups Diff. (Student t) | -- | -3.046 | -3.342 | -4.951 | -3.595 |
| 95% CI of LsMean |  | ~ 0.723 | ~ 0.467 | ~ -1.106 | ~ -0.105 |
| *p*-value (LsMean) | -- | 0.2218 | 0.1359 | 0.0026***** | 0.0381***** |
|  | ANCOVA1 (type 3 *p*-value): Model (<.0001*) with effect Treatment (0.0155*), Baseline (<.0001*) | | | | |
|  | ANCOVA2 (type 3 *p*-value): Model (0.0001*) with effect Treatment (0.0381*), Baseline (0.0003*) | | | | |
| **Week 12 – Baseline** | | | | | |
| N (Missing) | 8 (0) | 16 (0) | 15 (0) | 14 (0) | 45 (0) |
| Mean (SD) | -3.75 | -5.5 | -5.07 | -5.29 | -5.29 |
|  | -1.581 | -2.944 | -2.963 | -1.816 | -2.599 |
| Median (IQR) | -3.50 (2.00) | -5.00 (5.00) | -5.00 (3.00) | -6.00 (3.00) | -5.00 (4.00) |
| 95% CI (WI) | -5.072 | -7.069 | -6.708 | -7 | -6.07 |
|  | ~ -2.428t | ~ -3.931t | ~ -3.426t | ~ -4.000w | ~ -4.508t |
| *p*-value (WI) | 0.0003t* | <.0001t* | <.0001t* | 0.0001w* | <.0001t* |
| Groups Diff. (Student t) | -- | -3.555 | -3.066 | -3.573 | -3.047 |
| 95% CI of LsMean |  | ~0.153 | ~0.696 | ~0.238 | ~0.360 |
| *p*-value (LsMean) | -- | 0.0711 | 0.2111 | 0.0848 | 0.1195 |
|  | ANCOVA1 (type 3 *p*-value): Model (0.0009*) with effect Treatment (0.0555), Baseline (0.0362*), Treatment*Baseline (0.0304*) | | | | |
|  | ANCOVA2 (type 3 *p*-value): Model (0.0005*) with effect Treatment (0.1195), Baseline (0.0004*) | | | | |
| **Week 24 (LOCF) – Baseline** | | | | | |
| N (Missing) | 8 (0) | 17 (0) | 17 (0) | 15 (0) | 49 (0) |
| Mean (SD) | -4.75 | -4.94 | -6.65 | -5.67 | -5.76 |
|  | -3.105 | -3.473 | -2.827 | -3.619 | -3.32 |
| Median (IQR) | -4.00 (3.00) | -5.00 (4.00) | -7.00 (5.00) | -5.00 (4.00) | -5.00 (4.00) |
| 95% CI (WI) | -7.346 | -6.727 | -8.101 | -7 | -7 |
|  | ~ -2.154t | ~ -3.156t | ~ -5.193t | ~ -3.000w | ~ -4.000w |
| *p*-value (WI) | 0.0035t* | <.0001t* | <.0001t* | <.0001w* | <.0001w* |
| Groups Diff. (Student t) | -- | -1.855 | -2.814 | -3.134 | -2.348 |
| 95% CI of LsMean |  | ~ 2.157 | ~ 1.240 | ~ 0.960 | ~ 1.238 |
| *p*-value (LsMean) | -- | 0.8807 | 0.4394 | 0.2917 | 0.5376 |
|  | ANCOVA1 (type 3 *p*-value): Model (<.0001*) with effect Treatment (0.4157), Baseline (<.0001*) | | | | |
|  | ANCOVA2 (type 3 *p*-value): Model (<.0001*) with effect Treatment (0.5376), Baseline (<.0001*) | | | | |
| **Week 36 – Baseline** | | | | | |
| N (Missing) | 6 (0) | 12 (0) | 13 (0) | 14 (0) | 39 (0) |
| Mean (SD) | -6.67 | -5.83 | -6.46 | -5.57 | -5.95 |
|  | -2.944 | -4.108 | -2.57 | -3.817 | -3.479 |
| Median (IQR) | -7.00 (4.00) | -6.00 (5.50) | -6.00 (2.00) | -6.00 (5.00) | -6.00 (4.00) |
| 95% CI (WI) | -9.756 | -8.444 | -8.014 | -7.775 | -7.076 |
|  | ~ -3.577t | ~ -3.223t | ~ -4.909t | ~ -3.367t | ~ -4.821t |
| *p*-value (WI) | 0.0026t* | 0.0005t* | <.0001t* | 0.0001t* | <.0001t* |
| Groups Diff. (Student t) | -- | -1.361 | -1.413 | -1.569 | -1.116 |
| 95% CI of LsMean |  | ~ 3.732 | ~ 3.643 | ~ 3.397 | ~ 3.241 |
| *p*-value (LsMean) | -- | 0.3524 | 0.3781 | 0.4612 | 0.3307 |
|  | ANCOVA1 (type 3 *p*-value): Model (<.0001*) with effect Treatment (0.7999), Baseline (<.0001*) | | | | |
|  | ANCOVA2 (type 3 *p*-value): Model (<.0001*) with effect Treatment (0.3307), Baseline (<.0001*) | | | | |
| **Week 48 (LOCF) – Baseline** | | | | | |
| N (Missing) | 8 (0) | 17 (0) | 17 (0) | 15 (0) | 49 (0) |
| Mean (SD) | -4.25 (2.765) | -4.53 | -6.88 | -5.13 | -5.53 |
|  |  | -4.474 | -3.018 | -3.944 | -3.911 |
| Median (IQR) | -3.50 (3.50) | -5.00 (7.00) | -6.00 (1.00) | -5.00 (5.00) | -6.00 (4.00) |
| 95% CI (WI) | -6.561 | -6.83 | -8.434 | -7.317 | -6.654 |
|  | ~ -1.939t | ~ -2.229t | ~ -5.330t | ~ -2.949t | ~ -4.407t |
| *p*-value (WI) | 0.0034t* | 0.0007t* | <.0001t* | 0.0002t* | <.0001t* |
| Groups Diff. (Student t) | -- | -2.191 | -3.702 | -3.418 | -2.824 |
| 95% CI of LsMean |  | ~ 2.403 | ~ 0.939 | ~ 1.268 | ~ 1.297 |
| *p*-value (LsMean) | -- | 0.9266 | 0.2376 | 0.3616 | 0.4609 |
|  | ANCOVA1 (type 3 *p*-value): Model (<.0001*) with effect Treatment (0.3350), Baseline (<.0001*) | | | | |
|  | ANCOVA2 (type 3 *p* -value): Model (<.0001*) with effect Treatment (0.4609), Baseline (<.0001*) | | | | |

**Table S3**

**Summary of WOMAC Total Score**

| **WOMAC-Total Score** | | | | | |
| --- | --- | --- | --- | --- | --- |
|  | **HA** | **16M** | **32M** | **64M** | **Pooled** |
| **Baseline** | | | | | |
| N (Missing) | 8 (0) | 17 (0) | 17 (0) | 15 (0) | 49 (0) |
| Mean (SD) | 41.50 (15.024) | 42.88 (18.378) | 46.41 (17.969) | 35.27 (18.183) | 41.78 (18.383) |
| Median (IQR) | 41.50 (19.50) | 34.00 (25.00) | 44.00 (29.00) | 32.00 (22.00) | 35.00 (28.00) |
| Groups Diff. | -- | 0.8626 | 0.6248 | 0.2567 | 0.7749 |
| *p*-value (Wilcox_t) |  |  |  |  |  |
| **Week 2 – Baseline** | | | | | |
| N (Missing) | 8 (0) | 17 (0) | 16 (0) | 15 (0) | 48 (0) |
| Mean (SD) | -1.75 (10.278) | -13.41 (19.758) | -12.06 (16.039) | -6.47 (27.790) | -10.79 (21.308) |
| Median (IQR) | -5.00 (10.50) | -11.00 (18.00) | -11.50 (12.50) | -9.00 (28.00) | -10.50 (18.50) |
| 95% CI (WI) | -10.343 | -23.57 | -20.609 | -21.856 | -16 |
|  | ~ 6.843t | ~ -3.253t | ~ -3.516t | ~8.923t | ~ -8.000w |
| *p*-value (WI) | 0.6448t | 0.0129t* | 0.0088t* | 0.3827t | <.0001w* |
| Groups Diff. (Student t) | -- | -26.478 | -24.495 | -24.243 | -22.385 |
| 95% CI of LsMean |  | ~ 4.709 | ~ 7.034 | ~ 7.798 | ~ 4.361 |
| *p*-value (LsMean) | -- | 0.1672 | 0.2714 | 0.3077 | 0.1821 |
|  | ANCOVA1 (type 3 *p*-value): Model (0.0034*) with effect Treatment (0.5725), Baseline (0.0003*) | | | | |
|  | ANCOVA2 (type 3 *p*-value): Model (0.0003*) with effect Treatment (0.2263), Baseline (0.3089), Treatment*Baseline (0.0802) | | | | |
| **Week 4 – Baseline** | | | | | |
| N (Missing) | 8 (0) | 17 (0) | 17 (0) | 15 (0) | 49 (0) |
| Mean (SD) | -6.25 (11.585) | -15.59 (14.305) | -17.65 (11.258) | -18.33 (13.462) | -17.14 (12.834) |
| Median (IQR) | -7.50 (17.50) | -14.00 (14.00) | -18.00 (19.00) | -16.00 (23.00) | -15.00 (19.00) |
| 95% CI (WI) | -15.935 | -22.943 | -26 | -25.789 | -20.829 |
|  | ~ 3.435t | ~ -8.233t | ~ -7.000w | ~ -10.878t | ~ -13.457t |
| *p*-value (WI) | 0.1709t | 0.0004t* | <.0001w* | 0.0001t* | <.0001t* |
| Groups Diff. (Student t) | -- | -17.328 | -17.793 | -23.811 | -18.55 |
| 95% CI of LsMean |  | ~ -0.076 | ~ -0.479 | ~ -6.094 | ~ -2.999 |
| *p*-value (LsMean) | -- | 0.0481***** | 0.0390***** | 0.0014***** | 0.0075***** |
|  | ANCOVA1 (type 3 *p*-value): Model (<.0001*) with effect Treatment (0.0146*), Baseline (<.0001*) | | | | |
|  | ANCOVA2 (type 3 *p*-value): Model (<.0001*) with effect Treatment (0.0075*), Baseline (<.0001*) | | | | |
| **Week 12 – Baseline** | | | | | |
| N (Missing) | 8 (0) | 16 (0) | 15 (0) | 14 (0) | 45 (0) |
| Mean (SD) | -15.38 (11.904) | -23.00 (16.669) | -20.60 (15.099) | -18.71 (8.783) | -20.87 (13.900) |
| Median (IQR) | -16.50 (19.50) | -18.00 (23.50) | -17.00 (24.00) | -20.00 (11.00) | -19.00 (17.00) |
| 95% CI (WI) | -25.327 | -31.882 | -28.961 | -23.785 | -25.043 |
|  | ~ -5.423t | ~ -14.118t | ~ -12.239t | ~ -13.643t | ~ -16.691t |
| *p*-value (WI) | 0.0081t* | <.0001t* | 0.0001t* | <.0001t* | <.0001t* |
| Groups Diff. (Student t) | -- | -14.646 | -12.932 | -13.881 | -13.66 |
| 95% CI of LsMean |  | ~ 2.849 | ~ 4.753 | ~ 4.221 | ~ 2.272 |
| *p*-value (LsMean) | -- | 0.1812 | 0.3566 | 0.2882 | 0.1573 |
|  | ANCOVA1 (type 3 *p*-value): Model (<.0001*) with effect Treatment (0.1515), Baseline (<.0001*), Treatment*Baseline (0.0585) | | | | |
|  | ANCOVA2 (type 3 *p*-value): Model (<.0001*) with effect Treatment (0.1573), Baseline (<.0001*) | | | | |
| **Week 24 (LOCF) – Baseline** | | | | | |
| N (Missing) | 8 (0) | 17 (0) | 17 (0) | 15 (0) | 49 (0) |
| Mean (SD) | -16.63 (16.741) | -21.18 (17.569) | -26.65 (14.326) | -19.93 (20.041) | -22.69 (17.239) |
| Median (IQR) | -17.00 (20.00) | -18.00 (28.00) | -26.00 (24.00) | -19.00 (30.00) | -21.00 (28.00) |
| 95% CI (WI) | -30.621 | -30.209 | -34.013 | -31.032 | -27.645 |
|  | ~ -2.629t | ~ -12.144t | ~ -19.281t | ~ -8.835t | ~ -17.742t |
| *p*-value (WI) | 0.0262t* | 0.0001t* | <.0001t* | 0.0018t* | <.0001t* |
| Groups Diff. (Student t) | -- | -13.54 | -16.491 | -18.082 | -14.675 |
| 95% CI of LsMean |  | ~ 6.440 | ~ 3.561 | ~ 2.436 | ~ 2.931 |
| *p*-value (LsMean) | -- | 0.4789 | 0.2014 | 0.1321 | 0.1867 |
|  | ANCOVA1 (type 3 *p*-value): Model (<.0001*) with effect Treatment (0.4166), Baseline (<.0001*) | | | | |
|  | ANCOVA2 (type 3 *p*-value): Model (<.0001*) with effect Treatment (0.1867), Baseline (<.0001*) | | | | |
| **Week 36 – Baseline** | | | | | |
| N (Missing) | 6 (0) | 12 (0) | 13 (0) | 14 (0) | 39 (0) |
| Mean (SD) | -23.00 (16.888) | -25.50 (24.251) | -23.92 (11.857) | -21.14 (20.784) | -23.41 (19.125) |
| Median (IQR) | -28.50 (23.00) | -22.50 (39.50) | -23.00 (18.00) | -21.50 (24.00) | -22.00 (20.00) |
| 95% CI (WI) | -40.723 | -40.908 | -31.088 | -33.143 | -29.61 |
|  | ~ -5.277t | ~ -10.092t | ~ -16.758t | ~ -9.142t | ~ -17.211t |
| *p*-value (WI) | 0.0206t* | 0.0039t* | <.0001t* | 0.0022t* | <.0001t* |
| Groups Diff. (Student t) | -- | -13.325 | -12.104 | -15.126 | -12.03 |
| 95% CI of LsMean |  | ~ 10.134 | ~ 11.049 | ~ 7.910 | ~ 8.148 |
| *p*-value (LsMean) | -- | 0.7848 | 0.9271 | 0.5303 | 0.6998 |
|  | ANCOVA1 (type 3 *p*-value): Model (<.0001*) with effect Treatment (0.8904), Baseline (<.0001*) | | | | |
|  | ANCOVA2 (type 3 *p*-value): Model (<.0001*) with effect Treatment (0.6998), Baseline (<.0001*) | | | | |
| **Week 48 (LOCF) – Baseline** | | | | | |
| N (Missing) | 8 (0) | 17 (0) | 17 (0) | 15 (0) | 49 (0) |
| Mean (SD) | -15.75 (13.339) | -20.35 (23.553) | -27.76 (14.699) | -21.87 (20.770) | -23.39 (19.838) |
| Median (IQR) | -15.50 (18.00) | -10.00 (37.00) | -24.00 (21.00) | -22.00 (27.00) | -22.00 (29.00) |
| 95% CI (WI) | -26.902 | -40 | -35.322 | -33.369 | -29.086 |
|  | ~ -4.598t | ~ -3.000w | ~ -20.207t | ~ -10.364t | ~ -17.690t |
| *p*-value (WI) | 0.0124t* | 0.0009w* | <.0001t* | 0.0011t* | <.0001t* |
| Groups Diff. (Student t) | -- | -12.174 | -18.158 | -21.808 | -15.999 |
| 95% CI of LsMean |  | ~ 5.906 | ~ 0.117 | ~ -2.928 | ~ 1.200 |
| *p*-value (LsMean) | -- | 0.4893 | 0.0529 | 0.0113***** | 0.0902 |
|  | ANCOVA1 (type 3 *p*-value): Model (<.0001*) with effect Treatment (0.0455*), Baseline (<.0001*), Treatment*Baseline (0.0524) | | | | |
|  | ANCOVA2 (type 3 *p*-value): Model (<.0001*) with effect Treatment (0.0902), Baseline (<.0001*) | | | | |

**Table S4**

**Summary of WOMAC Stiffness Score**

| **WOMAC-Stiffness Score** | | | | | |
| --- | --- | --- | --- | --- | --- |
| **Groups** | **HA** | **16M** | **32M** | **64M** | **Pooled** |
| **Baseline** | | | | | |
| N (Missing) | 8 (0) | 17 (0) | 17 (0) | 15 (0) | 49 (0) |
| Mean (SD) | 3.13 (1.458) | 3.35 (1.766) | 3.53 (1.807) | 2.73 (1.792) | 3.22 (1.783) |
| Median (IQR) | 3.00 (1.00) | 3.00 (3.00) | 3.00 (2.00) | 2.00 (1.00) | 3.00 (3.00) |
| Groups Diff. | -- | 0.9531 | 0.6276 | 0.2693 | 0.8147 |
| *p*-value (Wilcox_t) |  |  |  |  |  |
| **Week 2 – Baseline** | | | | | |
| N (Missing) | 8 (0) | 17 (0) | 16 (0) | 15 (0) | 48 (0) |
| Mean (SD) | 0.38 | -0.94 (2.164) | -0.88 (1.258) | -0.40 (2.694) | -0.75 (2.078) |
|  | -1.598 |  |  |  |  |
| Median (IQR) | 0.50 (3.00) | -1.00 (2.00) | -1.00 (2.00) | -1.00 (4.00) | -1.00 (2.00) |
| 95% CI (WI) | -0.961 | -2.054 | -1.546 | -1.892 | -1 |
|  | ~ 1.711t | ~ 0.172t | ~ -0.204t | ~ 1.092t | ~ 0.000w |
| *p*-value (WI) | 0.5281t | 0.0919t | 0.0140t* | 0.5744t | 0.0080w* |
| Groups Diff. (Student t) | -- | -2.617 | -2.551 | -2.532 | -2.367 |
| 95% CI of LsMean |  | ~ 0.296 | ~ 0.392 | ~ 0.447 | ~ 0.174 |
| *p*-value (LsMean) | -- | 0.1158 | 0.1469 | 0.1662 | 0.0894 |
|  | ANCOVA1 (type 3 *p*-value): Model (<.0001*) with effect Treatment (0.4119), Baseline (<.0001*) | | | | |
|  | ANCOVA2 (type 3 *p*-value): Model (<.0001*) with effect Treatment (0.0894), Baseline (<.0001*) | | | | |
| **Week 4 – Baseline** | | | | | |
| N (Missing) | 8 (0) | 17 (0) | 17 (0) | 15 (0) | 49 (0) |
| Mean (SD) | 0 | -1.24 (1.786) | -1.35 (1.169) | -1.40 (1.724) | -1.33 (1.546) |
|  | -1.773 |  |  |  |  |
| Median (IQR) | -0.50 (3.50) | 0.00 (3.00) | -1.00 (1.00) | -2.00 (3.00) | -1.00 (3.00) |
| 95% CI (WI) | -1.482 | -3 | -1.954 | -2.355 | -2 |
|  | ~ 1.482t | ~ 0.000w | ~ -0.752t | ~ -0.445t | ~ 0.000w |
| *p*-value (WI) | 1.0000t | 0.0195w* | 0.0002t* | 0.0072t* | <.0001w* |
| Groups Diff. (Student t) | -- | -2.117 | -2.127 | -2.692 | -2.177 |
| 95% CI of LsMean |  | ~ -0.069 | ~ -0.075 | ~ -0.597 | ~ -0.356 |
| *p*-value (LsMean) | -- | 0.0369***** | 0.0360***** | 0.0027***** | 0.0073***** |
|  | ANCOVA1 (type 3 *p*-value): Model (<.0001*) with effect Treatment (0.0270*), Baseline (<.0001*) | | | | |
|  | ANCOVA2 (type 3 *p*-value): Model (<.0001*) with effect Treatment (0.0073*), Baseline (<.0001*) | | | | |
| **Week 12 – Baseline** | | | | | |
| N (Missing) | 8 (0) | 16 (0) | 15 (0) | 14 (0) | 45 (0) |
| Mean (SD) | -1.00 (1.852) | -1.81 (2.040) | -1.40 (1.298) | -1.64 (1.499) | -1.62 (1.628) |
| Median (IQR) | -1.50 (3.00) | -1.50 (2.50) | -1.00 (3.00) | -2.00 (1.00) | -2.00 (2.00) |
| 95% CI (WI) | -2.548 | -2.9 | -2.119 | -2.508 | -2.111 |
|  | ~ 0.548t | ~ -0.725t | ~ -0.681t | ~ -0.777t | ~ -1.133t |
| *p*-value (WI) | 0.1705t | 0.0029t* | 0.0009t* | 0.0013t* | <.0001t* |
| Groups Diff. (Student t) | -- | -1.636 | -1.354 | -1.848 | -1.469 |
| 95% CI of LsMean |  | ~ 0.451 | ~ 0.753 | ~ 0.286 | ~ 0.360 |
| *p*-value (LsMean) | -- | 0.259 | 0.569 | 0.1474 | 0.229 |
|  | ANCOVA1 (type 3 *p*-value): Model (<.0001*) with effect Treatment (0.4564), Baseline (<.0001*) | | | | |
|  | ANCOVA2 (type 3 *p*-value): Model (<.0001*) with effect Treatment (0.2290), Baseline (<.0001*) | | | | |
| **Week 24 (LOCF) – Baseline** | | | | | |
| N (Missing) | 8 (0) | 17 (0) | 17 (0) | 15 (0) | 49 (0) |
| Mean (SD) | -1.13 (2.167) | -1.41 (1.906) | -2.35 (1.618) | -1.53 (2.386) | -1.78 (1.982) |
| Median (IQR) | -1.00 (3.50) | -1.00 (2.00) | -3.00 (2.00) | -2.00 (2.00) | -2.00 (3.00) |
| 95% CI (WI) | -2.937 | -2.392 | -3.185 | -2.855 | -2.345 |
|  | ~ 0.687t | ~ -0.432t | ~ -1.521t | ~ -0.212t | ~ -1.206t |
| *p*-value (WI) | 0.1855t | 0.0076t* | <.0001t* | 0.0260t* | <.0001t* |
| Groups Diff. (Student t) | -- | -1.056 | -1.928 | -1.913 | -1.496 |
| 95% CI of LsMean |  | ~ 0.942 | ~ 0.078 | ~ 0.152 | ~ 0.377 |
| *p*-value (LsMean) | -- | 0.9094 | 0.0699 | 0.0928 | 0.2362 |
|  | ANCOVA1 (type 3 *p*-value): Model (<.0001*) with effect Treatment (0.0637), Baseline (<.0001*), Treatment*Baseline (0.0870) | | | | |
|  | ANCOVA2 (type 3 *p*-value): Model (<.0001*) with effect Treatment (0.2362), Baseline (<.0001*) | | | | |
| **Week 36 – Baseline** | | | | | |
| N (Missing) | 6 (0) | 12 (0) | 13 (0) | 14 (0) | 39 (0) |
| Mean (SD) | -1.00 (1.897) | -2.25 (2.454) | -2.08 (1.441) | -1.57 (2.138) | -1.95 (2.012) |
| Median (IQR) | -1.00 (3.00) | -2.00 (3.50) | -2.00 (2.00) | -2.00 (3.00) | -2.00 (3.00) |
| 95% CI (WI) | -2.991 | -4 | -2.948 | -2.806 | -2.601 |
|  | ~0.991t | ~0.000w | ~ -1.206t | ~ -0.337t | ~ -1.296t |
| *p*-value (WI) | 0.2532t | 0.0156w* | 0.0002t* | 0.0165t* | <.0001t* |
| Groups Diff. (Student t) | -- | -1.857 | -1.924 | -1.76 | -1.692 |
| 95% CI of LsMean |  | ~ 0.452 | ~ 0.348 | ~ 0.483 | ~ 0.277 |
| *p*-value (LsMean) | -- | 0.2262 | 0.1685 | 0.2566 | 0.1545 |
|  | ANCOVA1 (type 3 *p*-value): Model (<.0001*) with effect Treatment (0.5529), Baseline (<.0001*) | | | | |
|  | ANCOVA2 (type 3 *p*-value): Model (<.0001*) with effect Treatment (0.1545), Baseline (<.0001*) | | | | |
| **Week 48 (LOCF) – Baseline** | | | | | |
| N (Missing) | 8 (0) | 17 (0) | 17 (0) | 15 (0) | 49 (0) |
| Mean (SD) | -1.00 (1.852) | -1.59 (2.399) | -2.12 (1.453) | -1.60 (2.384) | -1.78 (2.084) |
| Median (IQR) | -1.50 (3.00) | -1.00 (3.00) | -2.00 (2.00) | -2.00 (3.00) | -1.00 (3.00) |
| 95% CI (WI) | -2.548 | -3 | -2.865 | -2.92 | -2.374 |
|  | ~0.548t | ~0.000w | ~ -1.371t | ~ -0.280t | ~ -1.177t |
| *p*-value (WI) | 0.1705t | 0.0166w* | <.0001t* | 0.0210t* | <.0001t* |
| Groups Diff. (Student t) | -- | -1.369 | -1.924 | -2.166 | -1.671 |
| 95% CI of LsMean |  | ~ 0.676 | ~ 0.131 | ~ -0.052 | ~ 0.303 |
| *p*-value (LsMean) | -- | 0.4991 | 0.0857 | 0.0402***** | 0.1707 |
|  | ANCOVA1 (type 3 *p*-value): Model (<.0001*) with effect Treatment (0.0169*), Baseline (<.0001*), Treatment*Baseline (0.0083*) | | | | |
|  | ANCOVA2 (type 3 *p*-value): Model (<.0001*) with effect Treatment (0.1707), Baseline (<.0001*) | | | | |

**Table S5**

**Summary of WOMAC Functional Limitation Score**

| **WOMAC-Functional Limitation Score** | | | | | |
| --- | --- | --- | --- | --- | --- |
| **Groups** | **HA** | **16M** | **32M** | **64M** | **Pooled** |
| **Baseline** | | | | | |
| N (Missing) | 8 (0) | 17 (0) | 17 (0) | 15 (0) | 49 (0) |
| Mean (SD) | 29.50 (12.513) | 30.24 (14.459) | 32.65 (13.684) | 23.87 (14.060) | 29.12 (14.256) |
| Median (IQR) | 29.00 (16.00) | 24.00 (18.00) | 31.00 (23.00) | 22.00 (16.00) | 24.00 (19.00) |
| Groups Diff. | -- | 0.7512 | 0.6866 | 0.2328 | 0.689 |
| *p*-value (Wilcox_t) |  |  |  |  |  |
| **Week 2 – Baseline** | | | | | |
| N (Missing) | 8 (0) | 17 (0) | 16 (0) | 15 (0) | 48 (0) |
| Mean (SD) | -1.25 | -9.12 (14.164) | -8.13 (11.904) | -3.80 (20.323) | -7.13 (15.552) |
|  | -7.888 |  |  |  |  |
| Median (IQR) | -4.00 (10.50) | -8 | -8.5 | -5 | -8 |
|  |  | -13 | -11 | -20 | -13 |
| 95% CI (WI) | -7.844 | -16.4 | -14.468 | -15.055 | -9 |
|  | ~ 5.344t | ~ -1.835t | ~ -1.782t | ~7.455t | ~ -3.000w |
| *p*-value (WI) | 0.6675t | 0.0173t* | 0.0155t* | 0.4809t | <.0001w* |
| Groups Diff. (Student t) | -- | -17.678 | -16.941 | -18.324 | -15.415 |
| 95% CI of LsMean |  | ~ 4.022 | ~ 5.075 | ~ 4.260 | ~ 3.699 |
| *p*-value (LsMean) | -- | 0.2119 | 0.2839 | 0.2166 | 0.2242 |
|  | ANCOVA1 (type 3 *p*-value): Model (0.0014*) with effect Treatment (0.2533), Baseline (0.0028*), Treatment*Baseline (0.0941) | | | | |
|  | ANCOVA2 (type 3 *p*-value): Model (<.0001*) with effect Treatment (0.2012), Baseline (0.1978), Treatment*Baseline (0.0571) | | | | |
| **Week 4 – Baseline** | | | | | |
| N (Missing) | 8 (0) | 17 (0) | 17 (0) | 15 (0) | 49 (0) |
| Mean (SD) | -4.25 | -11.00 (11.242) | -12.24 (8.693) | -12.00 (9.907) | -11.73 (9.810) |
|  | -9.161 |  |  |  |  |
| Median (IQR) | -3.50 (14.00) | -10.00 (12.00) | -11.00 (13.00) | -12.00 (15.00) | -11.00 (13.00) |
| 95% CI (WI) | -11.909 | -16.78 | -16.705 | -17.486 | -14.553 |
|  | ~ 3.409t | ~ -5.220t | ~ -7.766t | ~ -6.514t | ~ -8.917t |
| *p*-value (WI) | 0.2309t | 0.0010t* | <.0001t* | 0.0003t* | <.0001t* |
| Groups Diff. (Student t) | -- | -12.718 | -12.804 | -16.981 | -13.326 |
| 95% CI of LsMean |  | ~ -0.071 | ~ -0.125 | ~ -3.964 | ~ -1.987 |
| *p*-value (LsMean) | -- | 0.0476***** | 0.0458***** | 0.0022***** | 0.0091***** |
|  | ANCOVA1 (type 3 *p*-value): Model (<.0001*) with effect Treatment (0.0222*), Baseline (<.0001*) | | | | |
|  | ANCOVA2 (type 3 *p*-value): Model (<.0001*) with effect Treatment (0.0091*), Baseline (<.0001*) | | | | |
| **Week 12 – Baseline** | | | | | |
| N (Missing) | 8 (0) | 16 (0) | 15 (0) | 14 (0) | 45 (0) |
| Mean (SD) | -10.63 (9.680) | -15.69 (12.584) | -14.13 (11.495) | -11.79 (7.138) | -13.96 (10.662) |
| Median (IQR) | -10.00 (16.00) | -14.50 (18.00) | -12.00 (20.00) | -12.50 (9.00) | -13.00 (14.00) |
| 95% CI (WI) | -18.717 | -22.393 | -20.499 | -15.907 | -17.159 |
|  | ~ -2.533t | ~ -8.982t | ~ -7.768t | ~ -7.664t | ~ -10.752t |
| *p*-value (WI) | 0.0172t* | 0.0002t* | 0.0003t* | <.0001t* | <.0001t* |
| Groups Diff. (Student t) | -- | -10.321 | -9.32 | -9.455 | -9.725 |
| 95% CI of LsMean |  | ~ 2.721 | ~ 3.858 | ~ 4.068 | ~ 2.047 |
| *p*-value (LsMean) | -- | 0.2467 | 0.4082 | 0.4266 | 0.1962 |
|  | ANCOVA1 (type 3 *p*-value): Model (<.0001*) with effect Treatment (0.2174), Baseline (<.0001*), Treatment*Baseline (0.0746) | | | | |
|  | ANCOVA2 (type 3 *p*-value): Model (<.0001*) with effect Treatment (0.1962), Baseline (<.0001*) | | | | |
| **Week 24 (LOCF) – Baseline** | | | | | |
| N (Missing) | 8 (0) | 17 (0) | 17 (0) | 15 (0) | 49 (0) |
| Mean (SD) | -10.75 (12.116) | -14.82 (12.866) | -17.65 (10.931) | -12.73 (14.704) | -15.16 (12.733) |
| Median (IQR) | -10.50 (14.50) | -12.00 (20.00) | -18.00 (21.00) | -11.00 (22.00) | -13.00 (20.00) |
| 95% CI (WI) | -20.879 | -21.439 | -23.267 | -20.876 | -18.821 |
|  | ~ -0.621t | ~ -8.209t | ~ -12.027t | ~ -4.590t | ~ -11.506t |
| *p*-value (WI) | 0.0404t* | 0.0002t* | <.0001t* | 0.0047t* | <.0001t* |
| Groups Diff. (Student t) | -- | -11.061 | -12.271 | -13.502 | -11.224 |
| 95% CI of LsMean |  | ~ 3.910 | ~ 2.738 | ~ 1.907 | ~ 1.894 |
| *p*-value (LsMean) | -- | 0.3423 | 0.2082 | 0.1371 | 0.1596 |
|  | ANCOVA1 (type 3 *p*-value): Model (<.0001*) with effect Treatment (0.4838), Baseline (<.0001*) | | | | |
|  | ANCOVA2 (type 3 *p*-value): Model (<.0001*) with effect Treatment (0.1596), Baseline (<.0001*) | | | | |
| **Week 36 – Baseline** | | | | | |
| N (Missing) | 6 (0) | 12 (0) | 13 (0) | 14 (0) | 39 (0) |
| Mean (SD) | -15.33 (13.337) | -17.42 (18.382) | -15.38 (9.042) | -14.00 (15.452) | -15.51 (14.398) |
| Median (IQR) | -20.50 (17.00) | -15.50 (31.00) | -17.00 (14.00) | -14.50 (17.00) | -16.00 (18.00) |
| 95% CI (WI) | -29.329 | -29.096 | -20.849 | -22.922 | -20.18 |
|  | ~ -1.337t | ~ -5.737t | ~ -9.921t | ~ -5.078t | ~ -10.845t |
| *p*-value (WI) | 0.0373t* | 0.0073t* | <.0001t* | 0.0048t* | <.0001t* |
| Groups Diff. (Student t) | -- | -10.67 | -9.338 | -12.298 | -9.69 |
| 95% CI of LsMean |  | ~ 6.639 | ~ 7.751 | ~ 4.754 | ~ 5.279 |
| *p*-value (LsMean) | -- | 0.6405 | 0.8521 | 0.3765 | 0.5552 |
|  | ANCOVA1 (type 3 *p*-value): Model (<.0001*) with effect Treatment (0.7656), Baseline (<.0001*) | | | | |
|  | ANCOVA2 (type 3 *p*-value): Model (<.0001*) with effect Treatment (0.5552), Baseline (<.0001*) | | | | |
| **Week 48 (LOCF) – Baseline** | | | | | |
| N (Missing) | 8 (0) | 17 (0) | 17 (0) | 15 (0) | 49 (0) |
| Mean (SD) | -10.50 (11.123) | -14.24 (17.181) | -18.76 (11.245) | -15.13 (15.175) | -16.08 (14.551) |
| Median (IQR) | -11.50 (17.50) | -6 | -17.00 (15.00) | -13.00 (19.00) | -15.00 (22.00) |
|  |  | -25 |  |  |  |
| 95% CI (WI) | -19.799 | -26 | -24.546 | -23.537 | -20.261 |
|  | ~ -1.201t | ~ -1.000w | ~ -12.983t | ~ -6.730t | ~ -11.902t |
| *p*-value (WI) | 0.0320t* | 0.0022w* | <.0001t* | 0.0017t* | <.0001t* |
| Groups Diff. (Student t) | -- | -9.725 | -12.17 | -16.366 | -11.974 |
| 95% CI of LsMean |  | ~ 3.536 | ~ 1.125 | ~ -2.718 | ~ 0.178 |
| *p*-value (LsMean) | -- | 0.3533 | 0.1015 | 0.0070***** | 0.0569 |
|  | ANCOVA1 (type 3 *p*-value): Model (<.0001*) with effect Treatment (0.0324*), Baseline (<.0001*) | | | | |
|  | ANCOVA2 (type 3 *p*-value): Model (<.0001*) with effect Treatment (0.0569), Baseline (<.0001*) | | | | |

**Table S6**

**Summary of Visual Analogue Scale for Pain [mm]**

| **VAS for Pain** [**mm**] | | | | | |
| --- | --- | --- | --- | --- | --- |
| **Groups** | **HA** | **16M** | **32M** | **64M** | **Pooled** |
| **Baseline (Day 1)** | | | | | |
| N (Missing) | 8 (0) | 17 (0) | 17 (0) | 15 (0) | 49 (0) |
| Mean (SD) | 53.25 (18.805) | 52.00 (19.442) | 58.88 (22.039) | 55.27 (24.291) | 55.39 (21.643) |
| Median (IQR) | 52.50 (28.50) | 53.00 (25.00) | 64.00 (42.00) | 54.00 (36.00) | 54.00 (34.00) |
| Groups Diff. | -- | 0.8809 | 0.5398 | 0.8405 | 0.7934 |
| *p*-value (T test) |  |  |  |  |  |
| **Week 2 – Baseline** | | | | | |
| N (Missing) | 8 (0) | 17 (0) | 16 (0) | 15 (0) | 48 (0) |
| Mean (SD) | -5.25 (17.653) | -19.94 (29.057) | -10.44 (29.212) | -20.20 (35.489) | -16.85 (30.919) |
| Median (IQR) | -9.50 (17.50) | -16.00 (37.00) | -11.00 (37.00) | -23.00 (51.00) | -17.00 (40.00) |
| 95% CI (WI) | -20.009 | -34.881 | -26.003 | -39.853 | -30 |
|  | ~ 9.509t | ~ -5.001t | ~ 5.128t | ~ -0.547t | ~ -8.000w |
| *p*-value (WI) | 0.4281t | 0.0121t* | 0.1734t | 0.0447t* | 0.0002w* |
| Groups Diff. (Student t) | -- | -38.112 | -25.143 | -36.637 | -30.716 |
| 95% CI of LsMean |  | ~ 7.034 | ~ 20.534 | ~ 9.473 | ~ 9.609 |
| *p*-value (LsMean) | -- | 0.173 | 0.8403 | 0.2424 | 0.2985 |
|  | ANCOVA1 (type 3 *p*-value): Model (0.0026*) with effect Treatment (0.3330), Baseline (0.0002*) | | | | |
|  | ANCOVA2 (type 3 *p*-value): Model (0.0008*) with effect Treatment (0.2985), Baseline (0.0003*) | | | | |
| **Week 4 – Baseline** | | | | | |
| N (Missing) | 8 (0) | 17 (0) | 17 (0) | 15 (0) | 49 (0) |
| Mean (SD) | -8.38 (14.725) | -20.59 (18.228) | -25.06 (20.762) | -33.27 (31.880) | -26.02 (24.043) |
| Median (IQR) | -13.00 (20.00) | -22.00 (35.00) | -23.00 (26.00) | -32.00 (37.00) | -25.00 (35.00) |
| 95% CI (WI) | -19 | -29.96 | -35.734 | -50.921 | -32.926 |
|  | ~ 16.000w | ~ -11.216t | ~ -14.384t | ~ -15.612t | ~ -19.114t |
| *p*-value (WI) | 0.1953w | 0.0003t* | 0.0001t* | 0.0012t* | <.0001t* |
| Groups Diff. (Student t) | -- | -30.064 | -30.637 | -40.779 | -31.846 |
| 95% CI of LsMean |  | ~ 0.578 | ~ 0.029 | ~ -9.626 | ~ -3.986 |
| *p*-value (LsMean) | -- | 0.0589 | 0.0504 | 0.0021***** | 0.0127***** |
|  | ANCOVA1 (type 3 *p*-value): Model (<.0001*) with effect Treatment (0.2848), Baseline (0.0005*), Treatment*Baseline (0.0249*) | | | | |
|  | ANCOVA2 (type 3 *p*-value): Model (<.0001*) with effect Treatment (0.0990), Baseline (0.2405), Treatment*Baseline (0.0131*) | | | | |
| **Week 12 – Baseline** | | | | | |
| N (Missing) | 8 (0) | 16 (0) | 15 (0) | 14 (0) | 45 (0) |
| Mean (SD) | -13.25 (13.285) | -30.94 (20.680) | -28.00 (24.281) | -38.86 (23.091) | -32.42 (22.618) |
| Median (IQR) | -10.00 (16.50) | -27.50 (22.00) | -21.00 (47.00) | -36.00 (27.00) | -28.00 (31.00) |
| 95% CI (WI) | -24.357 | -41.957 | -41.446 | -52.19 | -39.217 |
|  | ~ -2.143t | ~ -19.918t | ~ -14.554t | ~ -25.525t | ~ -25.627t |
| *p*-value (WI) | 0.0257t* | <.0001t* | 0.0005t* | <.0001t* | <.0001t* |
| Groups Diff. (Student t) | -- | -28.876 | -22.996 | -33.932 | -27.389 |
| 95% CI of LsMean |  | ~ -6.499 | ~ -0.335 | ~ -10.990 | ~ -6.948 |
| *p*-value (LsMean) | -- | 0.0026***** | 0.0439***** | 0.0003***** | 0.0014***** |
|  | ANCOVA1 (type 3 *p*-value): Model (<.0001*) with effect Treatment (0.0019*), Baseline (<.0001*) | | | | |
|  | ANCOVA2 (type 3 *p*-value): Model (<.0001*) with effect Treatment (0.0014*), Baseline (<.0001*) | | | | |
| **Week 24 (LOCF) – Baseline** | | | | | |
| N (Missing) | 8 (0) | 17 (0) | 17 (0) | 15 (0) | 49 (0) |
| Mean (SD) | -22.50 (21.501) | -28.53 (23.752) | -38.65 (19.525) | -33.47 (31.284) | -33.55 (24.874) |
| Median (IQR) | -21.50 (31.50) | -21.00 (37.00) | -35.00 (30.00) | -34.00 (40.00) | -31.00 (39.00) |
| 95% CI (WI) | -40.475 | -40.741 | -48.686 | -50.791 | -40.696 |
|  | ~ -4.525t | ~ -16.317t | ~ -28.608t | ~ -16.142t | ~ -26.406t |
| *p*-value (WI) | 0.0211t* | 0.0001t* | <.0001t* | 0.0010t* | <.0001t* |
| Groups Diff. (Student t) | -- | -20.299 | -24.26 | -22.574 | -20.632 |
| 95% CI of LsMean |  | ~ 5.988 | ~ 2.115 | ~ 4.275 | ~ 2.421 |
| *p*-value (LsMean) | -- | 0.2797 | 0.098 | 0.1773 | 0.1191 |
|  | ANCOVA1 (type 3 *p*-value): Model (<.0001*) with effect Treatment (0.4017), Baseline (<.0001*) | | | | |
|  | ANCOVA2 (type 3 *p*-value): Model (<.0001*) with effect Treatment (0.1191), Baseline (<.0001*) | | | | |
| **Week 36 – Baseline** | | | | | |
| N (Missing) | 6 (0) | 12 (0) | 13 (0) | 14 (0) | 39 (0) |
| Mean (SD) | -32.67 (16.908) | -34.83 (26.474) | -39.85 (24.909) | -35.36 (26.011) | -36.69 (25.208) |
| Median (IQR) | -31.50 (34.00) | -37.00 (31.00) | -39.00 (41.00) | -35.50 (19.00) | -36.00 (33.00) |
| 95% CI (WI) | -50.41 | -51.654 | -54.899 | -50.375 | -44.864 |
|  | ~ -14.923t | ~ -18.012t | ~ -24.794t | ~ -20.339t | ~ -28.521t |
| *p*-value (WI) | 0.0052t* | 0.0008t* | <.0001t* | 0.0002t* | <.0001t* |
| Groups Diff. (Student t) | -- | -24.597 | -21.218 | -19.572 | -19.548 |
| 95% CI of LsMean |  | ~ 8.320 | ~ 11.139 | ~ 12.402 | ~ 8.640 |
| *p*-value (LsMean) | -- | 0.3236 | 0.5326 | 0.6528 | 0.4392 |
|  | ANCOVA1 (type 3 *p*-value): Model (<.0001*) with effect Treatment (0.7765), Baseline (<.0001*) | | | | |
|  | ANCOVA2 (type 3 *p*-value): Model (<.0001*) with effect Treatment (0.4392), Baseline (<.0001*) | | | | |
| **Week 48 (LOCF) – Baseline** | | | | | |
| N (Missing) | 8 (0) | 17 (0) | 17 (0) | 15 (0) | 49 (0) |
| Mean (SD) | -25.00 (25.873) | -27.06 (27.123) | -35.47 (20.932) | -38.53 (34.965) | -33.49 (27.782) |
| Median (IQR) | -18.00 (39.00) | -30.00 (39.00) | -35.00 (22.00) | -40.00 (45.00) | -35.00 (33.00) |
| 95% CI (WI) | -46.631 | -41.004 | -46.233 | -57.896 | -41.47 |
|  | ~ -3.369t | ~ -13.113t | ~ -24.708t | ~ -19.170t | ~ -25.510t |
| *p*-value (WI) | 0.0292t* | 0.0008t* | <.0001t* | 0.0008t* | <.0001t* |
| Groups Diff. (Student t) | -- | -17.841 | -20.41 | -25.968 | -19.291 |
| 95% CI of LsMean |  | ~ 10.629 | ~ 8.081 | ~ 2.976 | ~ 6.689 |
| *p*-value (LsMean) | -- | 0.613 | 0.3888 | 0.1169 | 0.3351 |
|  | ANCOVA1 (type 3 *p*-value): Model (<.0001*) with effect Treatment (0.4017), Baseline (<.0001*) | | | | |
|  | ANCOVA2 (type 3 *p*-value): Model (<.0001*) with effect Treatment (0.1191), Baseline (<.0001*) | | | | |

**Table S7**

**Summary of KSCRS Score - Objective Knee Indicators**

| **KSCRS – Objective Knee Indicators** | | | | | |
| --- | --- | --- | --- | --- | --- |
| **Groups** | **HA** | **16M** | **32M** | **64M** | **Pooled** |
| **Baseline (Day 1)** | | | | | |
| N (Missing) | 8 (0) | 17 (0) | 17 (0) | 15 (0) | 49 (0) |
| Mean (SD) | 70.13 (12.299) | 72.24 (6.379) | 70.47 (8.125) | 73.73 (4.920) | 72.08 (6.664) |
| Median (IQR) | 74.5 | 75.00 (10.00) | 74.00 (12.00) | 76 | 75.00 (10.00) |
|  | -7.5 |  |  | -8 |  |
| Groups Diff. | -- | 0.8843 | 0.7285 | 0.724 | 0.9633 |
| *p*-value (Wilcox_t) |  |  |  |  |  |
| **Week 12 – Baseline** | | | | | |
| N (Missing) | 8 (0) | 16 (0) | 15 (0) | 14 (0) | 45 (0) |
| Mean (SD) | -1.88 | 2.88 | 0.93 | 1.5 | 1.8 |
|  | -5.436 | -4.097 | -6.724 | -3.995 | -5.052 |
| Median (IQR) | 0.00 (4.00) | 1.00 (5.00) | 2.00 (4.00) | 0.00 (2.00) | 0.00 (3.00) |
| 95% CI (WI) | -7 | 0 | 0 | 0 | 0 |
|  | ~ 4.000w | ~ 5.000w | ~ 4.000w | ~ 3.000w | ~ 2.000w |
| *p*-value (WI) | 0.6250w | 0.0078w* | 0.2598w | 0.2500w | 0.0021w* |
| Groups Diff. (Student t) | -- | 1.26 | -0.565 | 0.281 | 0.602 |
| 95% CI of LsMean |  | ~ 9.374 | ~ 7.656 | ~ 8.655 | ~ 7.626 |
| *p*-value (LsMean) | -- | 0.0113***** | 0.0893 | 0.0370***** | 0.0226***** |
|  | ANCOVA1 (type 3 *p*-value): Model (0.0037*) with effect Treatment (0.0759), Baseline (0.0010*) | | | | |
|  | ANCOVA2 (type 3 *p*-value): Model (0.0004*) with effect Treatment (0.0326*), Baseline (0.0018*), Treatment*Baseline (0.0672) | | | | |
| **Week 24 – Baseline** | | | | | |
| N (Missing) | 8 (0) | 17 (0) | 17 (0) | 15 (0) | 49 (0) |
| Mean (SD) | -2.63 | 3.06 | 4.18 | -0.13 | 2.47 |
|  | -3.998 | -4.993 | -9.567 | -3.815 | -6.807 |
| Median (IQR) | -1.50 (6.50) | 0.00 (7.00) | 1.00 (5.00) | 0.00 (0.00) | 0.00 (3.00) |
| 95% CI (WI) | -5.967 | 0 | 0 | 0 | 0 |
|  | ~ 0.717t | ~ 7.000w | ~5.000w | ~ 0.000w | ~ 1.000w |
| *p*-value (WI) | 0.1056t | 0.0264w* | 0.0800w | 1.0000w | 0.0105w* |
| Groups Diff. (Student t) | -- | 2.361 | 1.851 | -1.054 | 1.791 |
| 95% CI of LsMean |  | ~9.600 | ~9.117 | ~6.536 | ~8.896 |
| *p*-value (LsMean) | -- | 0.0017***** | 0.0039***** | 0.153 | 0.0039***** |
|  | ANCOVA1 (type 3 *p*-value): Model (<.0001*) with effect Treatment (<.0001*), Baseline (<.0001*), Treatment*Baseline (<.0001*) | | | | |
|  | ANCOVA2 (type 3 *p*-value): Model (<.0001*) with effect Treatment (<.0001*), Baseline (<.0001*), Treatment*Baseline (<.0001*) | | | | |
| **Week 48 – Baseline** | | | | | |
| N (Missing) | 6 (0) | 12 (0) | 13 (0) | 12 (0) | 37 (0) |
| Mean (SD) | -3.17 (11.618) | 3.25 | 1.46 | 0.92 | 1.86 |
|  |  | -4.202 | -3.455 | -2.392 | -3.481 |
| Median (IQR) | 0.00 (7.00) | 2.00 (6.00) | 2.00 (3.00) | 0.00 (1.00) | 0.00 (3.00) |
| 95% CI (WI) | -15.359 | 0 | -0.626 | 0 | 0 |
|  | ~ 9.025t | ~ 8.000w | ~ 3.549t | ~ 2.000w | ~ 2.000w |
| *p*-value (WI) | 0.5339t | 0.0234w* | 0.1531t | 0.2500w | 0.0002w* |
| Groups Diff. (Student t) | -- | 0.276 | -1.141 | -1.112 | -0.051 |
| 95% CI of LsMean |  | ~ 10.286 | ~ 8.680 | ~ 8.759 | ~ 8.550 |
| *p*-value (LsMean) | -- | 0.0392***** | 0.1285 | 0.1251 | 0.0527 |
|  | ANCOVA1 (type 3 *p*-value): Model (0.0143*) with effect Treatment (0.2210), Baseline (0.0103*) | | | | |
|  | ANCOVA2 (type 3 *p*-value): Model (0.0025*) with effect Treatment (0.0527), Baseline (0.0063*) | | | | |

**Table S8**

**Summary of KSCRS Score – Symptoms**

| **KSCRS Score-Symptoms** | | | | | |
| --- | --- | --- | --- | --- | --- |
| **Groups** | **HA** | **16M** | **32M** | **64M** | **Pooled** |
| **Baseline (Day 1)** | | | | | |
| N (Missing) | 8 (0) | 17 (0) | 17 (0) | 15 (0) | 49 (0) |
| Mean (SD) | 9.5 | 10.47 (5.245) | 8.76 | 12.27 (4.949) | 10.43 (5.208) |
|  | -4.472 |  | -5.13 |  |  |
| Median (IQR) | 9 | 11 | 10.00 (10.00) | 13 | 11 |
|  | -6 | -9 |  | -5 | -8 |
| Groups Diff. | -- | 0.6443 | 0.7948 | 0.1968 | 0.5824 |
| *p*-value (Wilcox_t) |  |  |  |  |  |
| **Week 2 – Baseline** | | | | | |
| N (Missing) | 8 (0) | 17 (0) | 16 (0) | 15 (0) | 48 (0) |
| Mean (SD) | 1.50 (3.162) | 4.29 (5.059) | 3.88 (4.856) | 2.40 (6.599) | 3.56 (5.465) |
| Median (IQR) | 1.50 (5.00) | 3.00 (6.00) | 3.00 (5.50) | 3.00 (9.00) | 3.00 (6.00) |
| 95% CI (WI) | -1.144 | 1.693 | 1.287 | -1.254 | 1.976 |
|  | ~ 4.144t | ~ 6.895t | ~ 6.463t | ~ 6.054t | ~ 5.149t |
| *p*-value (WI) | 0.2216t | 0.0030t* | 0.0061t* | 0.1808t | <.0001t* |
| Groups Diff. (Student t) | -- | -0.909 | -1.917 | -2.13 | -1.666 |
| 95% CI of LsMean |  | ~ 7.381 | ~ 6.440 | ~ 6.448 | ~ 5.631 |
| *p*-value (LsMean) | -- | 0.1232 | 0.2824 | 0.3171 | 0.2806 |
|  | ANCOVA1 (type 3 *p*-value): Model (0.0130*) with effect Treatment (0.4884), Baseline (0.0014*) | | | | |
|  | ANCOVA2 (type 3 *p*-value): Model (0.0017*) with effect Treatment (0.0376*), Baseline (0.3902), Treatment*Baseline (0.0968) | | | | |
| **Week 4 – Baseline** | | | | | |
| N (Missing) | 8 (0) | 17 (0) | 17 (0) | 15 (0) | 49 (0) |
| Mean (SD) | 5.00 (2.878) | 5.35 (5.255) | 6.18 (4.864) | 5.67 (4.791) | 5.73 (4.890) |
| Median (IQR) | 5.00 (5.00) | 3.00 (8.00) | 7.00 (7.00) | 7.00 (9.00) | 6.00 (8.00) |
| 95% CI (WI) | 2.594 | 1 | 3.676 | 3.014 | 3 |
|  | ~ 7.406t | ~ 9.000w | ~ 8.677t | ~ 8.320t | ~ 8.000w |
| *p*-value (WI) | 0.0017t* | 0.0002w* | <.0001t* | 0.0004t* | <.0001w* |
| Groups Diff. (Student t) | -- | -1.692 | -2.077 | -0.197 | -1.098 |
| 95% CI of LsMean |  | ~ 3.775 | ~ 3.386 | ~ 5.457 | ~ 3.815 |
| *p*-value (LsMean) | -- | 0.4479 | 0.6325 | 0.0676 | 0.2725 |
|  | ANCOVA1 (type 3 *p*-value): Model (<.0001*) with effect Treatment (0.2288), Baseline (<.0001*) | | | | |
|  | ANCOVA2 (type 3 *p*-value): Model (<.0001*) with effect Treatment (0.2725), Baseline (<.0001*) | | | | |
| **Week 12 – Baseline** | | | | | |
| N (Missing) | 8 (0) | 16 (0) | 15 (0) | 14 (0) | 45 (0) |
| Mean (SD) | 4.63 (3.503) | 6.50 (4.412) | 5.87 (4.357) | 6.29 (4.250) | 6.22 (4.253) |
| Median (IQR) | 4.00 (5.50) | 6.50 (7.00) | 6.00 (6.00) | 6.50 (5.00) | 6.00 (5.00) |
| 95% CI (WI) | 1.697 | 4.149 | 3.454 | 3.832 | 4.945 |
|  | ~ 7.553t | ~ 8.851t | ~ 8.279t | ~ 8.740t | ~ 7.500t |
| *p*-value (WI) | 0.0073t* | <.0001t* | 0.0001t* | <.0001t* | <.0001t* |
| Groups Diff. (Student t) | -- | 0.195 | -1.017 | 0.895 | 0.233 |
| 95% CI of LsMean |  | ~ 4.803 | ~ 3.633 | ~ 5.660 | ~ 4.398 |
| *p*-value (LsMean) | -- | 0.0341***** | 0.2635 | 0.0080***** | 0.0300***** |
|  | ANCOVA1 (type 3 *p*-value): Model (<.0001*) with effect Treatment (0.0370*), Baseline (<.0001*) | | | | |
|  | ANCOVA2 (type 3 *p*-value): Model (<.0001*) with effect Treatment (0.0300*), Baseline (<.0001*) | | | | |
| **Week 24 (LOCF) – Baseline** | | | | | |
| N (Missing) | 8 (0) | 17 (0) | 17 (0) | 15 (0) | 49 (0) |
| Mean (SD) | 5.63 (4.596) | 6.53 (5.746) | 9.00 (5.327) | 6.07 (5.837) | 7.24 (5.666) |
| Median (IQR) | 6.50 (4.00) | 6.00 (6.00) | 9.00 (7.00) | 7.00 (8.00) | 8.00 (9.00) |
| 95% CI (WI) | 1.782 | 3.575 | 6.261 | 2.834 | 5.617 |
|  | ~ 9.468t | ~ 9.484t | ~ 11.739t | ~ 9.299t | ~ 8.872t |
| *p*-value (WI) | 0.0105t* | 0.0002t* | <.0001t* | 0.0013t* | <.0001t* |
| Groups Diff. (Student t) | -- | -1.044 | -0.082 | -0.011 | -0.036 |
| 95% CI of LsMean |  | ~ 4.572 | ~ 5.529 | ~ 5.797 | ~ 4.916 |
| *p*-value (LsMean) | -- | 0.213 | 0.0568 | 0.0508 | 0.0533 |
|  | ANCOVA1 (type 3 *p*-value): Model (<.0001*) with effect Treatment (0.1861), Baseline (<.0001*) | | | | |
|  | ANCOVA2 (type 3 *p*-value): Model (<.0001*) with effect Treatment (0.0533), Baseline (<.0001*) | | | | |
| **Week 36 – Baseline** | | | | | |
| N (Missing) | 6 (0) | 12 (0) | 13 (0) | 14 (0) | 39 (0) |
| Mean (SD) | 7.67 (5.203) | 7.33 (6.972) | 8.85 (5.178) | 6.57 (6.260) | 7.56 (6.073) |
| Median (IQR) | 7.50 (3.00) | 7.50 (12.00) | 9.00 (5.00) | 7.50 (8.00) | 8.00 (9.00) |
| 95% CI (WI) | 2.207 | 2.904 | 5.717 | 2.957 | 5.595 |
|  | ~ 13.126t | ~ 11.763t | ~ 11.975t | ~ 10.186t | ~ 9.533t |
| *p*-value (WI) | 0.0154t* | 0.0039t* | <.0001t* | 0.0017t* | <.0001t* |
| Groups Diff. (Student t) | -- | -1.537 | -2.095 | -1.326 | -1.238 |
| 95% CI of LsMean |  | ~ 5.182 | ~ 4.479 | ~ 5.295 | ~ 4.524 |
| *p*-value (LsMean) | -- | 0.2794 | 0.4678 | 0.2327 | 0.2562 |
|  | ANCOVA1 (type 3 *p*-value): Model (<.0001*) with effect Treatment (0.6453), Baseline (<.0001*) | | | | |
|  | ANCOVA2 (type 3 *p*-value): Model (<.0001*) with effect Treatment (0.2562), Baseline (<.0001*) | | | | |
| **Week 48 (LOCF) – Baseline** | | | | | |
| N (Missing) | 8 (0) | 17 (0) | 17 (0) | 15 (0) | 49 (0) |
| Mean (SD) | 5.13 (4.518) | 6.24 (5.847) | 7.53 (5.800) | 6.27 (6.943) | 6.69 (6.087) |
| Median (IQR) | 5.50 (8.50) | 6.00 (8.00) | 7.00 (8.00) | 8.00 (11.00) | 7.00 (8.00) |
| 95% CI (WI) | 1.348 | 1 | 4.547 | 2.422 | 4.946 |
|  | ~ 8.902t | ~ 9.000w | ~ 10.511t | ~ 10.112t | ~ 8.442t |
| *p*-value (WI) | 0.0149t* | 0.0001w* | <.0001t* | 0.0036t* | <.0001t* |
| Groups Diff. (Student t) | -- | -1.258 | -1.543 | 0.327 | -0.52 |
| 95% CI of LsMean |  | ~ 5.278 | ~ 4.988 | ~ 7.087 | ~ 5.309 |
| *p*-value (LsMean) | -- | 0.2227 | 0.2947 | 0.0322***** | 0.1053 |
|  | ANCOVA1 (type 3 *p*-value): Model (<.0001*) with effect Treatment (0.1793), Baseline (<.0001*) | | | | |
|  | ANCOVA2 (type 3 *p*-value): Model (<.0001*) with effect Treatment (0.1053), Baseline (<.0001*) | | | | |

**Table S9**

**Summary of KSCRS Score - Patient Satisfaction**

| **KSCRS Score-Patient Satisfaction** | | | | | |
| --- | --- | --- | --- | --- | --- |
| **Groups** | **HA** | **16M** | **32M** | **64M** | **Pooled** |
| **Baseline (Day 1)** | | | | | |
| N (Missing) | 8 (0) | 17 (0) | 17 (0) | 15 (0) | 49 (0) |
| Mean (SD) | 13.75 (4.713) | 19.18 (5.525) | 14.82 (7.971) | 17.73 (6.840) | 17.22 (6.962) |
| Median (IQR) | 15 | 20 | 16.00 (12.00) | 18 | 18.00 (10.00) |
|  | -7 | -8 |  | -8 |  |
| Groups Diff. | -- | 0.0253***** | 0.729 | 0.1578 | 0.1805 |
| *p*-value (T test) |  |  |  |  |  |
| **Week 2 – Baseline** | | | | | |
| N (Missing) | 8 (0) | 17 (0) | 16 (0) | 15 (0) | 48 (0) |
| Mean (SD) | 3.25 | 3.65 | 7.38 | 3.47 (12.293) | 4.83 |
|  | -4.892 | -9.226 | -8.024 |  | -9.892 |
| Median (IQR) | 2.00 (9.00) | 4.00 (8.00) | 6.00 (8.00) | 2.00 (18.00) | 4.00 (10.00) |
| 95% CI (WI) | -0.84 | 0 | 3.099 | -3.341 | 1.961 |
|  | ~ 7.340t | ~ 8.000w | ~ 11.651t | ~ 10.274t | ~ 7.706t |
| *p*-value (WI) | 0.1023t | 0.0359w* | 0.0022t* | 0.2932t | 0.0014t* |
| Groups Diff. (Student t) | -- | -2.238 | -1.252 | -3.621 | -5.674 |
| 95% CI of LsMean |  | ~ 11.876 | ~ 12.558 | ~ 10.546 | ~ 8.545 |
| *p*-value (LsMean) | -- | 0.1764 | 0.1064 | 0.3311 | 0.687 |
|  | ANCOVA1 (type 3 *p*-value): Model (0.0003*) with effect Treatment (0.4096), Baseline (<.0001*) | | | | |
|  | ANCOVA2 (type 3 *p*-value): Model (<.0001*) with effect Treatment (0.0370*), Baseline (0.2713), Treatment*Baseline (0.0899) | | | | |
| **Week 4 – Baseline** | | | | | |
| N (Missing) | 8 (0) | 17 (0) | 17 (0) | 15 (0) | 49 (0) |
| Mean (SD) | 7.50 (6.302) | 4.59 (7.238) | 9.76 (6.996) | 7.87 (9.606) | 7.39 (8.095) |
| Median (IQR) | 5.00 (8.00) | 4.00 (10.00) | 8.00 (12.00) | 8.00 (10.00) | 8.00 (10.00) |
| 95% CI (WI) | 2.231 | 0.867 | 6.168 | 2.547 | 5.063 |
|  | ~ 12.769t | ~ 8.309t | ~ 13.362t | ~ 13.186t | ~ 9.713t |
| *p*-value (WI) | 0.0120t* | 0.0188t* | <.0001t* | 0.0068t* | <.0001t* |
| Groups Diff. (Student t) | -- | -3.997 | -2.005 | -1.856 | -1.922 |
| 95% CI of LsMean |  | ~ 6.527 | ~ 8.187 | ~ 8.721 | ~ 7.208 |
| *p*-value (LsMean) | -- | 0.6316 | 0.229 | 0.1985 | 0.2508 |
|  | ANCOVA1 (type 3 *p*-value): Model (<.0001*) with effect Treatment (0.4698), Baseline (<.0001*) | | | | |
|  | ANCOVA2 (type 3 *p*-value): Model (<.0001*) with effect Treatment (0.2508), Baseline (<.0001*) | | | | |
| **Week 12 – Baseline** | | | | | |
| N (Missing) | 8 (0) | 16 (0) | 15 (0) | 14 (0) | 45 (0) |
| Mean (SD) | 8.50 (5.210) | 7.25 (6.017) | 8.80 (5.846) | 9.86 (7.941) | 8.58 (6.559) |
| Median (IQR) | 7 | 8 | 8 | 12.00 (10.00) | 8 |
|  | -5 | -10 | -8 |  | -8 |
| 95% CI (WI) | 4 | 4.044 | 5.563 | 5.272 | 6.607 |
|  | ~ 20.000w | ~ 10.456t | ~ 12.037t | ~ 14.442t | ~ 10.548t |
| *p*-value (WI) | 0.0078w* | 0.0002t* | <.0001t* | 0.0005t* | <.0001t* |
| Groups Diff. (Student t) | -- | -2.395 | -2.801 | -0.927 | -1.512 |
| 95% CI of LsMean |  | ~ 6.523 | ~ 5.965 | ~ 8.029 | ~ 6.217 |
| *p*-value (LsMean) | -- | 0.3567 | 0.4714 | 0.1174 | 0.2272 |
|  | ANCOVA1 (type 3 *p*-value): Model (<.0001*) with effect Treatment (0.4471), Baseline (<.0001*) | | | | |
|  | ANCOVA2 (type 3 *p*-value): Model (<.0001*) with effect Treatment (0.2272), Baseline (<.0001*) | | | | |
| **Week 24 (LOCF) – Baseline** | | | | | |
| N (Missing) | 8 (0) | 17 (0) | 17 (0) | 15 (0) | 49 (0) |
| Mean (SD) | 8.5 | 7.53 | 11.88 (5.808) | 10.00 (10.954) | 9.8 |
|  | -5.928 | -6.578 |  |  | -8.003 |
| Median (IQR) | 8 | 6 | 12 | 10.00 (14.00) | 10.00 (10.00) |
|  | -6 | -8 | -8 |  |  |
| 95% CI (WI) | 3.544 | 4.148 | 8.896 | 3.934 | 7.497 |
|  | ~ 13.456t | ~ 10.911t | ~ 14.869t | ~ 16.066t | ~ 12.095t |
| *p*-value (WI) | 0.0048t* | 0.0002t* | <.0001t* | 0.0033t* | <.0001t* |
| Groups Diff. (Student t) | -- | -1.072 | -0.285 | 0.143 | 0.21 |
| 95% CI of LsMean |  | ~ 8.393 | ~ 8.882 | ~ 9.656 | ~ 8.358 |
| *p*-value (LsMean) | -- | 0.1267 | 0.0654 | 0.0437***** | 0.0397***** |
|  | ANCOVA1 (type 3 *p*-value): Model (<.0001*) with effect Treatment (0.2049), Baseline (<.0001*) | | | | |
|  | ANCOVA2 (type 3 *p*-value): Model (<.0001*) with effect Treatment (0.0397*), Baseline (<.0001*) | | | | |
| **Week 36 – Baseline** | | | | | |
| N (Missing) | 6 (0) | 12 (0) | 13 (0) | 14 (0) | 39 (0) |
| Mean (SD) | 12.67 (9.004) | 8.67 | 15.38 (5.059) | 11.86 (9.906) | 12.05 (8.442) |
|  |  | -8.752 |  |  |  |
| Median (IQR) | 15.00 (16.00) | 9 | 14 | 13.00 (14.00) | 12.00 (10.00) |
|  |  | -13 | -6 |  |  |
| 95% CI (WI) | 3.218 | 3.106 | 12.328 | 6.138 | 9.315 |
|  | ~ 22.115t | ~ 14.228t | ~ 18.442t | ~ 17.577t | ~ 14.788t |
| *p*-value (WI) | 0.0183t* | 0.0056t* | <.0001t* | 0.0006t* | <.0001t* |
| Groups Diff. (Student t) | -- | -6.308 | -2.723 | -4.405 | -3.571 |
| 95% CI of LsMean |  | ~ 6.582 | ~ 9.686 | ~ 7.987 | ~ 7.518 |
| *p*-value (LsMean) | -- | 0.966 | 0.2635 | 0.5624 | 0.4765 |
|  | ANCOVA1 (type 3 *p*-value): Model (<.0001*) with effect Treatment (0.5332), Baseline (<.0001*) | | | | |
|  | ANCOVA2 (type 3 *p*-value): Model (<.0001*) with effect Treatment (0.4765), Baseline (<.0001*) | | | | |
| **Week 48 (LOCF) – Baseline** | | | | | |
| N (Missing) | 8 (0) | 17 (0) | 17 (0) | 15 (0) | 49 (0) |
| Mean (SD) | 5.75 | 6.71 | 11.18 (5.659) | 11.60 (12.147) | 9.76 |
|  | -5.701 | -7.174 |  |  | -8.714 |
| Median (IQR) | 7 | 6 | 10 | 14.00 (20.00) | 10.00 (10.00) |
|  | -11 | -8 | -4 |  |  |
| 95% CI (WI) | 0.984 | 2 | 8.267 | 4.873 | 7.252 |
|  | ~ 10.516t | ~ 10.000w | ~ 14.086t | ~ 18.327t | ~ 12.258t |
| *p*-value (WI) | 0.0246t* | 0.0005w* | <.0001t* | 0.0024t* | <.0001t* |
| Groups Diff. (Student t) | -- | -1.205 | 0.072 | 2.907 | 2.286 |
| 95% CI of LsMean |  | ~ 10.205 | ~ 11.287 | ~ 14.253 | ~ 11.754 |
| *p*-value (LsMean) | -- | 0.1194 | 0.0472***** | 0.0038***** | 0.0044***** |
|  | ANCOVA1 (type 3 *p*-value): Model (<.0001*) with effect Treatment (0.0038*), Baseline (<.0001*), Treatment*Baseline (0.0239*) | | | | |
|  | ANCOVA2 (type 3 *p*-value): Model (<.0001*) with effect Treatment (0.0044*), Baseline (<.0001*) | | | | |

**Table S10**

**Summary of KSCRS Score - Functional Activities**

| **KSCRS Score-Functional Activities** | | | | | |
| --- | --- | --- | --- | --- | --- |
| **Groups** | **HA** | **16M** | **32M** | **64M** | **Pooled** |
| **Baseline (Day 1)** | | | | | |
| N (Missing) | 8 (0) | 17 (0) | 17 (0) | 15 (0) | 49 (0) |
| Mean (SD) | 47.13 (18.435) | 54.35 (21.328) | 46.47 (16.148) | 60.53 (16.505) | 53.51 (18.741) |
| Median (IQR) | 50.50 (23.50) | 59.00 (34.00) | 43.00 (20.00) | 64.00 (16.00) | 55.00 (29.00) |
| Groups Diff. | -- | 0.4191 | 0.9287 | 0.089 | 0.3745 |
| *p*-value (T test) |  |  |  |  |  |
| **Week 2 – Baseline** | | | | | |
| N (Missing) | 8 (0) | 17 (0) | 16 (0) | 15 (0) | 48 (0) |
| Mean (SD) | 1.13 (9.342) | 5.12 (21.163) | 5.63 (11.159) | -3.93 (24.435) | 2.46 (19.725) |
| Median (IQR) | 1.00 (17.50) | 5.00 (18.00) | 6.50 (20.00) | 4.00 (28.00) | 5.00 (21.00) |
| 95% CI (WI) | -6.685 | -2 | -0.321 | -17.465 | 0 |
|  | ~ 8.935t | ~ 16.000w | ~ 11.571t | ~ 9.598t | ~ 9.000w |
| *p*-value (WI) | 0.7434t | 0.0733w | 0.0620t | 0.5430t | 0.0706w |
| Groups Diff. (Student t) | -- | -7.458 | -9.691 | -14.327 | -8.287 |
| 95% CI of LsMean |  | ~ 21.941 | ~ 19.758 | ~ 16.264 | ~ 17.681 |
| *p*-value (LsMean) | -- | 0.3273 | 0.4956 | 0.8993 | 0.4713 |
|  | ANCOVA1 (type 3 *p*-value): Model (0.0097*) with effect Treatment (0.6609), Baseline (0.0012*) | | | | |
|  | ANCOVA2 (type 3 *p*-value): Model (0.0019*) with effect Treatment (0.4713), Baseline (0.0004*) | | | | |
| **Week 4 – Baseline** | | | | | |
| N (Missing) | 8 (0) | 17 (0) | 17 (0) | 15 (0) | 49 (0) |
| Mean (SD) | 9.88 (13.400) | 11.24 (14.906) | 13.59 (6.634) | 9.67 (14.955) | 11.57 (12.513) |
| Median (IQR) | 6.50 (18.00) | 9.00 (11.00) | 14.00 (5.00) | 14.00 (25.00) | 13.00 (14.00) |
| 95% CI (WI) | -1.327 | 3.571 | 10.177 | 1.385 | 7.977 |
|  | ~ 21.077t | ~ 18.899t | ~ 16.999t | ~ 17.949t | ~ 15.166t |
| *p*-value (WI) | 0.0756t | 0.0068t* | <.0001t* | 0.0253t* | <.0001t* |
| Groups Diff. (Student t) | -- | -6.838 | -6.627 | -7.068 | -5.423 |
| 95% CI of LsMean |  | ~ 13.635 | ~ 13.684 | ~ 14.214 | ~ 12.416 |
| *p*-value (LsMean) | -- | 0.5082 | 0.4888 | 0.5035 | 0.4354 |
|  | ANCOVA1 (type 3 *p*-value): Model (0.0363*) with effect Treatment (0.8967), Baseline (0.0025*) | | | | |
|  | ANCOVA2 (type 3 *p*-value): Model (0.0054*) with effect Treatment (0.4354), Baseline (0.0014*) | | | | |
| **Week 12 – Baseline** | | | | | |
| N (Missing) | 8 (0) | 16 (0) | 15 (0) | 14 (0) | 45 (0) |
| Mean (SD) | 10.63 (13.049) | 15.50 (15.761) | 13.93 (8.556) | 12.14 (15.486) | 13.93 (13.444) |
| Median (IQR) | 9.5 | 12.50 (11.50) | 14.00 (14.00) | 12.50 (23.00) | 14.00 (15.00) |
|  | -16 |  |  |  |  |
| 95% CI (WI) | -0.284 | 7.102 | 9.195 | 3.201 | 9.894 |
|  | ~ 21.534t | ~ 23.898t | ~ 18.672t | ~ 21.084t | ~ 17.972t |
| *p*-value (WI) | 0.0547t | 0.0013t* | <.0001t* | 0.0116t* | <.0001t* |
| Groups Diff. (Student t) | -- | -2.758 | -5.989 | -3.743 | -2.807 |
| 95% CI of LsMean |  | ~ 17.420 | ~ 14.305 | ~ 17.319 | ~ 14.870 |
| *p*-value (LsMean) | -- | 0.1505 | 0.4141 | 0.2012 | 0.1766 |
|  | ANCOVA1 (type 3 *p*-value): Model (0.0012*) with effect Treatment (0.4870), Baseline (<.0001*) | | | | |
|  | ANCOVA2 (type 3 *p*-value): Model (0.0001*) with effect Treatment (0.1766), Baseline (<.0001*) | | | | |
| **Week 24 (LOCF) – Baseline** | | | | | |
| N (Missing) | 8 (0) | 17 (0) | 17 (0) | 15 (0) | 49 (0) |
| Mean (SD) | 15.13 (12.999) | 18.35 (19.468) | 19.00 (11.737) | 12.00 (15.390) | 16.63 (15.845) |
| Median (IQR) | 15.00 (18.00) | 13.00 (22.00) | 21.00 (13.00) | 15.00 (18.00) | 18.00 (20.00) |
| 95% CI (WI) | 4.257 | 8.344 | 12.966 | 3.477 | 12.081 |
|  | ~ 25.993t | ~ 28.362t | ~ 25.034t | ~ 20.523t | ~ 21.184t |
| *p*-value (WI) | 0.0133t* | 0.0013t* | <.0001t* | 0.0092t* | <.0001t* |
| Groups Diff. (Student t) | -- | -4.368 | -7.483 | -8.143 | -5.146 |
| 95% CI of LsMean |  | ~ 17.884 | ~ 14.594 | ~ 14.990 | ~ 14.377 |
| *p*-value (LsMean) | -- | 0.2284 | 0.5209 | 0.5552 | 0.3474 |
|  | ANCOVA1 (type 3 *p*-value): Model (0.0001*) with effect Treatment (0.6622), Baseline (<.0001*) | | | | |
|  | ANCOVA2 (type 3 *p*-value): Model (<.0001*) with effect Treatment (0.3474), Baseline (<.0001*) | | | | |
| **Week 36 – Baseline** | | | | | |
| N (Missing) | 6 (0) | 12 (0) | 13 (0) | 14 (0) | 39 (0) |
| Mean (SD) | 23.83 (10.265) | 21.92 (24.209) | 21.77 (13.027) | 15.57 (14.037) | 19.59 (17.319) |
| Median (IQR) | 24.50 (15.00) | 26.00 (34.00) | 19.00 (15.00) | 20.00 (18.00) | 20.00 (27.00) |
| 95% CI (WI) | 13.061 | 6.535 | 13.897 | 7.467 | 13.976 |
|  | ~ 34.606t | ~ 37.298t | ~ 29.641t | ~ 23.676t | ~ 25.204t |
| *p*-value (WI) | 0.0023t* | 0.0095t* | <.0001t* | 0.0011t* | <.0001t* |
| Groups Diff. (Student t) | -- | -8.942 | -11.371 | -10.894 | -13.223 |
| 95% CI of LsMean |  | ~ 15.334 | ~ 12.438 | ~ 13.330 | ~ 8.195 |
| *p*-value (LsMean) | -- | 0.5975 | 0.9283 | 0.84 | 0.638 |
|  | ANCOVA1 (type 3 *p*-value): Model (<.0001*) with effect Treatment (0.9346), Baseline (<.0001*) | | | | |
|  | ANCOVA2 (type 3 *p*-value): Model (<.0001*) with effect Treatment (0.0359*), Baseline (0.0046*), Treatment*Baseline (0.0331*) | | | | |
| **Week 48 (LOCF) – Baseline** | | | | | |
| N (Missing) | 8 (0) | 17 (0) | 17 (0) | 15 (0) | 49 (0) |
| Mean (SD) | 13.50 (13.470) | 17.76 (20.434) | 19.00 (13.024) | 13.73 (16.503) | 16.96 (16.737) |
| Median (IQR) | 13.50 (19.00) | 12.00 (26.00) | 20.00 (20.00) | 16.00 (20.00) | 16.00 (24.00) |
| 95% CI (WI) | 2.239 | 7.258 | 12.304 | 4.594 | 12.152 |
|  | ~ 24.761t | ~ 28.271t | ~ 25.696t | ~ 22.872t | ~ 21.767t |
| *p*-value (WI) | 0.0252t* | 0.0025t* | <.0001t* | 0.0061t* | <.0001t* |
| Groups Diff. (Student t) | -- | -5.428 | -8.151 | -6.017 | -5.995 |
| 95% CI of LsMean |  | ~ 16.220 | ~ 13.967 | ~ 16.906 | ~ 13.482 |
| *p*-value (LsMean) | -- | 0.3214 | 0.5996 | 0.3445 | 0.4441 |
|  | ANCOVA1 (type 3 *p*-value): Model (<.0001*) with effect Treatment (0.0080*), Baseline (<.0001*), Treatment*Baseline (0.0209*) | | | | |
|  | ANCOVA2 (type 3 *p*-value): Model (<.0001*) with effect Treatment (0.0051*), Baseline (0.0336*), Treatment*Baseline (0.0130*) | | | | |

**Table S11**

**Summary of Whole-Organ Magnetic Resonance Imaging Score (WORMS) in the total femorotibial joint.**

| **WORMS** | | | | | |
| --- | --- | --- | --- | --- | --- |
| **Groups** | **HA** | **16M** | **32M** | **64M** | **Pooled** |
| **Baseline (Screening)** | | | | | |
| N (Missing) | 8 (0) | 17 (0) | 17 (0) | 15 (0) | 49 (0) |
| Mean (SD) | 34.81 (20.530) | 26.44 (13.791) | 30.41 (12.425) | 34.27 (12.935) | 30.21 (13.185) |
| Median (IQR) | 42.25 (32.00) | 29.50 (17.50) | 28.50 (22.50) | 36.50 (18.00) | 30.50 (21.00) |
| Groups Diff | -- | 0.2387 | 0.5104 | 0.9381 | 0.4037 |
| *p*-value (T test) |  |  |  |  |  |
| **Week 24 – Baseline** | | | | | |
| N (Missing) | 8 (0) | 17 (0) | 17 (0) | 15 (0) | 49 (0) |
| Mean (SD) | -0.63 (10.763) | 5.50 (12.972) | 1.29 (10.668) | 4.43 (11.603) | 3.71 (11.690) |
| Median (IQR) | -1.75 (8.00) | 1.00 (8.00) | 3.50 (9.00) | 2.00 (12.50) | 2.00 (9.00) |
| 95% CI (WI) | -9.623 | 0 | -4.191 | -1.992 | 0 |
|  | ~ 8.373t | ~ 8.000w | ~ 6.779t | ~ 10.859t | ~ 5.500w |
| *p*-value (WI) | 0.8742t | 0.0916w | 0.6238t | 0.1611t | 0.0180w* |
| Groups Diff. (Student t) | -- | -5.977 | -8.869 | -4.722 | -5.386 |
| 95% CI of LsMean |  | ~ 13.186 | ~ 10.057 | ~ 14.510 | ~ 11.351 |
| *p*-value (LsMean) | -- | 0.4537 | 0.9002 | 0.3119 | 0.4779 |
|  | ANCOVA1 (type 3 *p*-value): Model (0.0434*) with effect Treatment (0.6132), Baseline (0.0061*) | | | | |
|  | ANCOVA2 (type 3 *p*-value): Model (0.0131*) with effect Treatment (0.4779), Baseline (0.0056*) | | | | |
| **Week 48 – Baseline** | | | | | |
| N (Missing) | 5 (1) | 12 (0) | 13 (0) | 12 (0) | 37 (0) |
| Mean (SD) | -3.00 (9.702) | 1.71 (6.576) | 3.46 (8.410) | 4.75 (16.351) | 3.31 (10.956) |
| Median (IQR) | -3.50 (4.50) | 0.75 (9.50) | 6.00 (7.00) | 7.50 (19.75) | 4.50 (11.50) |
| 95% CI (WI) | -15.046 | -2.47 | -1.621 | -5.639 | -0.342 |
|  | ~ 9.046t | ~ 5.887t | ~ 8.544t | ~ 15.139t | ~ 6.964t |
| *p*-value (WI) | 0.5273t | 0.3875t | 0.1636t | 0.3359t | 0.0743t |
| Groups Diff. (Student t) | -- | -6.94 | -3.355 | -2.063 | -2.932 |
| 95% CI of LsMean |  | ~ 15.729 | ~ 19.204 | ~ 20.784 | ~ 17.217 |
| *p*-value (LsMean) | -- | 0.4371 | 0.163 | 0.1053 | 0.1596 |
|  | ANCOVA1 (type 3 *p*-value): Model (0.1521) with effect Treatment (0.3505), Baseline (0.0306*) | | | | |
|  | ANCOVA2 (type 3 *p*-value): Model (0.0645) with effect Treatment (0.1596), Baseline (0.0455*) | | | | |
| **Week 72 – Baseline** | | | | | |
| N (Missing) | 3 (0) | 6 (0) | 7 (0) | 5 (0) | 18 (0) |
| Mean (SD) | 1.50 (6.384) | 1.58 (5.766) | 5.79 (10.610) | 7.90 (8.721) | 4.97 (8.617) |
| Median (IQR) | 0.00 (12.50) | 0.00 (2.50) | 5.50 (15.00) | 5.50 (11.00) | 3.00 (13.50) |
| 95% CI (WI) | -14.358 | -3 | -4.027 | -2.928 | 0.687 |
|  | ~ 17.358t | ~ 13.000w | ~ 15.598t | ~ 18.728t | ~ 9.257t |
| *p*-value (WI) | 0.7234t | 1.0000w | 0.1992t | 0.1128t | 0.0255t* |
| Groups Diff. (Student t) | -- | -12.593 | -8.085 | -6.692 | -7.504 |
| 95% CI of LsMean |  | ~ 12.759 | ~ 16.656 | ~ 19.492 | ~ 14.449 |
| *p*-value (LsMean) | -- | 0.9891 | 0.4748 | 0.3168 | 0.5159 |
|  | ANOVA1 (type 3 *p*-value): Model (0.5760) with effect Treatment (0.5760) | | | | |
|  | ANOVA2 (type 3 *p*-value): Model (0.5159) with effect Treatment (0.5159) | | | | |
| **Week 96 – Baseline** | | | | | |
| N (Missing) | 3 (0) | 7 (0) | 10 (0) | 7 (0) | 24 (0) |
| Mean (SD) | 0.17 (5.838) | 6.21 (8.553) | 3.05 (11.577) | 8.14 (9.915) | 5.46 (10.103) |
| Median (IQR) | -1.00 (11.50) | 2.00 (13.00) | 0.50 (10.50) | 6.50 (9.50) | 5.75 (11.00) |
| 95% CI (WI) | -14.336 | -1.696 | -5.232 | -1.027 | 1.192 |
|  | ~ 14.669t | ~ 14.125t | ~ 11.332t | ~ 17.313t | ~ 9.724t |
| *p*-value (WI) | 0.9651t | 0.1029t | 0.4263t | 0.0728t | 0.0144t* |
| Groups Diff. (Student t) | -- | -8.237 | -10.744 | -6.309 | -7.106 |
| 95% CI of LsMean |  | ~ 20.333 | ~ 16.510 | ~ 22.261 | ~ 17.689 |
| *p*-value (LsMean) | -- | 0.3902 | 0.6657 | 0.2599 | 0.3877 |
|  | ANOVA1 (type 3 *p*-value): Model (0.6074) with effect Treatment (0.6074) | | | | |
|  | ANOVA2 (type 3 *p*-value): Model (0.3877) with effect Treatment (0.3877) | | | | |

**Table S12**

**Summary of Mean of Cartilage Thickness [mm]**

| **MRI-Mean of Cartilage Thickness [mm]** | | | | | |
| --- | --- | --- | --- | --- | --- |
| **Groups** | **HA** | **16M** | **32M** | **64M** | **Pooled** |
| **Baseline (Screening)** | | | | | |
| N (Missing) | 8 (0) | 14 (3) | 17 (0) | 13 (2) | 44 (5) |
| Mean (SD) | 10.95 (3.475) | 11.30 (1.748) | 10.68 (2.568) | 10.23 (2.426) | 10.74 (2.281) |
| Median (IQR) | 10.28 (3.78) | 10.91 (2.19) | 11.65 (3.79) | 11.05 (3.04) | 11.25 (3.26) |
| Group Diff. | -- | 0.7944 | 0.8275 | 0.5807 | 0.8301 |
| *p*-value (T test) |  |  |  |  |  |
| **Week 24 – Baseline** | | | | | |
| N (Missing) | 8 (0) | 14 (3) | 16 (1) | 12 (3) | 42 (7) |
| Mean (SD) | 0.06 | -0.25 (0.649) | -0.15 (1.823) | -0.02 (1.812) | -0.14 (1.496) |
|  | -1.291 |  |  |  |  |
| Median (IQR) | 0.18 (1.94) | -0.10 (0.55) | -0.39 (1.63) | -0.24 (2.84) | -0.19 (1.46) |
| 95% CI (WI) | -1.018 | -0.624 | -0.71 | -1.167 | -0.68 |
|  | ~1.140t | ~0.125t | ~0.940w | ~1.135t | ~0.320w |
| *p*-value (WI) | 0.8970t | 0.1735t | 0.9799w | 0.9764t | 0.5261w |
| Groups Diff. (Student t) | -- | -1.517 | -1.524 | -1.529 | -1.332 |
| 95% CI of LsMean |  | ~1.033 | ~0.969 | ~1.105 | ~0.837 |
| *p*-value (LsMean) | -- | 0.7041 | 0.656 | 0.747 | 0.6488 |
|  | ANCOVA1 (type 3 *p*-value): Model (0.2254) with effect Treatment (0.9747), Baseline (0.0224*) | | | | |
|  | ANCOVA2 (type 3 *p*-value): Model (0.0556) with effect Treatment (0.6488), Baseline (0.0181*) | | | | |
| **Week 48 – Baseline** | | | | | |
| N (Missing) | 5 (1) | 9 (3) | 13 (0) | 11 (1) | 33 (4) |
| Mean (SD) | -1.20 (1.431) | -0.32 (1.152) | 0.02 | 0.16 | -0.03 (1.750) |
|  |  |  | -1.917 | -2.051 |  |
| Median (IQR) | -1.61 (1.15) | -0.98 (1.92) | -0.24 (2.41) | -0.17 (3.77) | -0.24 (2.02) |
| 95% CI (WI) | -2.979 | -1.185 | -1.142 | -1.218 | -0.646 |
|  | ~0.575t | ~0.890w | ~1.175t | ~1.539t | ~0.594t |
| *p*-value (WI) | 0.1336t | 0.2031w | 0.9763t | 0.8006t | 0.9323t |
| Groups Diff. (Student t) | -- | -1.296 | -0.807 | -0.809 | -0.371 |
| 95% CI of LsMean |  | ~2.492 | ~2.760 | ~2.869 | ~2.725 |
| p-value (LsMean) | -- | 0.5252 | 0.2734 | 0.2626 | 0.1314 |
|  | ANCOVA1 (type 3 *p*-value): Model (0.1181) with effect Treatment (0.6573), Baseline (0.0269*) | | | | |
|  | ANCOVA2 (type 3 *p*-value): Model (0.0105*) with effect Treatment (0.0247*), Baseline (0.1345), Treatment*Baseline (0.0437*) | | | | |
| **Week 72 – Baseline** | | | | | |
| N (Missing) | 3 (0) | 6 (0) | 7 (0) | 5 (0) | 18 (0) |
| Mean (SD) | -0.28 (1.301) | -0.42 (1.005) | -0.44 (2.096) | -0.45 (2.576) | -0.44 (1.846) |
| Median (IQR) | -0.55 (2.56) | -0.51 (0.79) | 0.29 (2.84) | 0.13 (2.47) | -0.12 (2.46) |
| 95% CI (WI) | -3.512 | -1.475 | -2.379 | -3.65 | -1.355 |
|  | ~2.952t | ~0.635t | ~1.498t | ~2.746t | ~0.481t |
| *p*-value (WI) | 0.7452t | 0.3532t | 0.5981t | 0.7148t | 0.3295t |
| Groups Diff. (Student t) | -- | -4.136 | -4.523 | -4.925 | -2.93 |
| 95% CI of LsMean |  | ~0.941 | ~0.646 | ~0.614 | ~2.259 |
| *p*-value (LsMean) | -- | 0.2008 | 0.1314 | 0.1185 | 0.7883 |
|  | ANCOVA1 (type 3 *p*-value): Model (0.0976) with effect Treatment (0.4152), Baseline (0.0074*) | | | | |
|  | ANCOVA2 (type 3 *p*-value): Model (0.0095*) with effect Treatment (0.1489), Baseline (0.0988), Treatment*Baseline (0.0598) | | | | |
| **Week 96 – Baseline** | | | | | |
| N (Missing) | 3 (0) | 7 (0) | 10 (0) | 7 (0) | 24 (0) |
| Mean (SD) | -0.91 (1.658) | -0.41 (1.278) | -0.28 (2.536) | -1.21 (1.073) | -0.59 (1.847) |
| Median (IQR) | -0.74 (3.30) | -0.25 (2.17) | -0.41 (2.73) | -0.96 (1.91) | -0.82 (2.58) |
| 95% CI (WI) | -5.026 | -1.588 | -2.096 | -2.203 | -1.91 |
|  | ~3.213t | ~0.776t | ~1.532t | ~ -0.218t | ~0.455w |
| *p*-value (WI) | 0.4436t | 0.4332t | 0.7332t | 0.0245t* | 0.0396w* |
| Groups Diff. (Student t) | -- | -3.142 | -3.58 | -3.999 | -3.529 |
| 95% CI of LsMean |  | ~1.866 | ~1.329 | ~1.010 | ~0.441 |
| *p*-value (LsMean) | -- | 0.6 | 0.3494 | 0.2269 | 0.1214 |
|  | ANCOVA1 (type 3 *p*-value): Model (0.0031*) with effect Treatment (0.0603), Baseline (0.0045*), Treatment*Baseline (0.0679) | | | | |
|  | ANCOVA2 (type 3 *p*-value): Model (0.0010*) with effect Treatment (0.1214), Baseline (0.0002*) | | | | |

**Supplementary information 1 of Table S2-S12**

- For CI and p-value (WI, within group), t denotes by using one sample t-test, w denotes CI of median and p-value using Wilcoxon signed rank test (determined by Shapiro-Wilk normality test).
- For CI and p-value (between groups), Wilcox_t denotes Wilcoxon rank-sum test (Mann-Whitney U Test) in t approximation.
- Treatment in ANOVA1/ANCOVA1 are 3 ELIXCYTE groups and HA, in ANOVA2/ANCOVA2 are ELIXCYTE Pooled and HA.
- Baseline was Visit 2 or alternatively as Visit 1 if Visit 2 data is not available.
- For signs of significance, * representing a p-value < 0.05.

**Supplementary information 2 of MRI changes**

- Change from baseline to post-treatment visit in cartilage thickness in the total femorotibial joint by centralized (independent) imaging interpretation.
- Knee MRI changes were analyzed by using ANCOVA incorporating treatment effect and baseline as covariate or by Wilcoxon rank-sum test. Categorical parameters of knee MRI changes were analyzed by using the Chi-square test or Fisher’s exact test. Chi-Square test was used when no cell has expected count < 1 and ≤ 20% of the cells have expected count < 5, otherwise Fisher's exact test would be applied.
- Pairwise treatment group comparisons were conducted with a significance level of 0.05 without alpha adjustment. For efficacy endpoints, a 95% two-sided confidence interval on the difference of each treatment was provided as appropriate.

**Supplementary information 3 of screen failures**

| Number  of subject | Subjects ID | Reasons for screen failure |
| --- | --- | --- |
| 1 | 01-003 | Not meeting “Western Ontario and McMaster Universities Osteoarthritis Index (WOMAC) pain score of 7-17 in the study knee even if treated with chronic doses of non-steroidal anti-inflammatory drugs”  With judged to be not applicable to this study by investigator such as the difficulty of follow-up observation |
| 3 | 01-005  01-012  01-024 | With any other serious diseases/medical history considered by the investigator not in the condition to enter the trial |
| 1 | 01-015 | With joint diseases except for knee osteoarthritis, and was considered by the investigator not eligible to enter the study |
| 1 | 01-022 | Not meeting “Kellgren-Lawrence grading I-III, as determined by American College of Rheumatology (ACR) criteria for osteoarthritis of the knee” |
| 1 | 02-003 | Not meeting “Having provided informed consent” |
